# Hepatic Lipid Stress Drives Metabolic Dysfunction‐Associated Steatohepatitis Progression through FTO *O*‐GlcNAcylation‐Induced m6A Demethylation of *HRAS*

**DOI:** 10.1002/advs.78218

**Published:** 2026-10-11

**Authors:** Jia‐Xin Yin, Yan‐Lai Zhang, Qun Liu, Rui Liu, Hai‐Yang Yuan, Sui‐Dan Chen, Ming‐Hua Zheng, Kai Wang, Ni Tang, Ai‐Long Huang

**Affiliations:** ^1^ Department of Infectious Diseases Key Laboratory of Molecular Biology for Infectious Diseases (Ministry of Education) Institute for Viral Hepatitis The Second Affiliated Hospital Chongqing Medical University Chongqing China; ^2^ MAFLD Research Center Department of Hepatology The First Affiliated Hospital of Wenzhou Medical University Wenzhou China; ^3^ Key Laboratory of Diagnosis and Treatment for the Development of Chronic Liver Disease in Zhejiang Province Wenzhou China; ^4^ Department of Pathology The First Affiliated Hospital of Wenzhou Medical University Wenzhou China

**Keywords:** epigenetic reprogramming, FTO, HRAS, lipid stress, MASH, *O*‐GlcNAcylation, PI3K/AKT

## Abstract

Metabolic dysfunction‐associated steatohepatitis (MASH) is driven by nutrient stress through incompletely defined epigenetic mechanisms. This study demonstrates that lipid stress activates the hexosamine biosynthesis pathway (HBP), elevating *O*‐linked β‐N‐acetylglucosamine (*O*‐GlcNAc) modification of the N6‐methyladenosine (m6A) demethylase fat mass and obesity‐associated protein (FTO) at Ser195. This post‐translational modification recruits the deubiquitinase ubiquitin‐specific peptidase 20 (USP20), displaces the E3 ligase F‐box and WD repeat domain containing 7 (FBXW7), and enhances FTO protein stability and m6A demethylase activity. *O*‐GlcNAc‐dependent FTO activation removes m6A from Harvey rat sarcoma viral oncogene homolog (*HRAS*) mRNA, evades YTH N6‑methyladenosine RNA binding protein 2 (YTHDF2)‑mediated degradation, and prolongs transcript half‐life. Subsequent HRAS upregulation activates the PI3K/AKT signaling pathway, driving hepatic steatosis and inflammation. Administration of a novel cell‐penetrating peptide (CPP) designed to block FTO *O*‐GlcNAcylation at Ser195 effectively ameliorates MASH progression in vitro and in vivo. These findings establish FTO *O*‐GlcNAcylation as a druggable metabolic‐epigenetic checkpoint in MASH pathogenesis, revealing a promising therapeutic strategy for this increasingly prevalent liver disease.

## Graphical abstract

Lipid stress drives *O*‐GlcNAcylation of the m6A demethylase FTO at Ser195, enhancing its stability and demethylase activity. This modification removes m6A from *HRAS* mRNA, preventing its degradation and activating PI3K/AKT signaling to promote hepatic steatosis and inflammation. Targeting this metabolic‐epigenetic checkpoint with a cell‐penetrating peptide ameliorates MASH, revealing a promising therapeutic strategy.

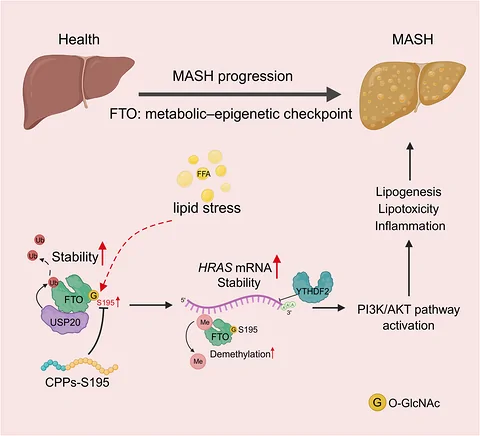

## 1 Introduction

Metabolic dysfunction‐associated steatotic liver disease (MASLD) has emerged as a substantial and growing global health burden, currently affecting approximately 30% of the worldwide population, with prevalence projected to reach 50% by 2040 [1, 2]. The disease spectrum ranges from simple steatosis to metabolic dysfunction‐associated steatohepatitis (MASH), fibrosis, cirrhosis, and hepatocellular carcinoma (HCC) [3]. MASH, a key progressive stage, is characterized by hepatic steatosis accompanied by lobular inflammation, hepatocellular ballooning, and fibrosis, which collectively elevate the risk of end‐stage liver disease and mortality [4]. The complex pathogenesis and considerable inter‐individual heterogeneity of MASH make therapeutic intervention particularly challenging. Although resmetirom and semaglutide have received FDA approval for MASH treatment [5, 6, 7], their limited efficacy and specificity underscore the urgent need to elucidate novel pathogenic mechanisms and identify actionable therapeutic targets.

Excessive hepatic lipid accumulation is recognized as a central driver of MASH pathogenesis; however, the underlying molecular mechanism converting nutrient excess into disease progression remains incompletely understood. Lipotoxic stress—characterized by lipid overload, oxidative injury, and mitochondrial impairment—activates multiple nutrient‐sensing and stress‐responsive pathways. Among these, *O*‐linked β‐N‐acetylglucosamine (*O*‐GlcNAc) modification has emerged as a principal nutrient‐sensitive mechanism. *O*‐GlcNAcylation is a dynamic post‐translational modification (PTM) regulated by *O*‐GlcNAc transferase (OGT), which uses uridine diphosphate N‐acetylglucosamine (UDP‐GlcNAc) from the hexosamine biosynthetic pathway (HBP), and is removed by *O*‐GlcNAcase (OGA). This modification serves as a critical nutrient sensor that modulates protein function in response to metabolic stress, regulating multiple aspects of MASH pathogenesis including insulin resistance [8], lipogenesis [9, 10], and inflammation [11, 12]. Although *O*‐GlcNAcylation is upregulated under lipid overload and correlates with MASH progression [9, 11, 12], the molecular mechanisms by which lipid stress activates *O*‐GlcNAc signaling—particularly its potential role in regulating epigenetic or epitranscriptomic reprogramming—remain poorly defined. Elucidating these mechanisms may reveal novel therapeutic opportunities for MASH.

The fat mass and obesity‐associated protein (FTO), initially identified as an obesity susceptibility gene through genome‐wide association studies (GWAS) [13], was later characterized as a novel demethylase for N6‐methyladenosine (m6A) modification [14]. Recent evidence indicates that m6A methylation is dysregulated in MASH, an alteration linked to the upregulation of FTO [15, 16, 17]. Although FTO has been shown to promote hepatic lipid deposition in an m6A‐dependent manner [18], the mechanism driving its aberrant expression in MASH pathogenesis remains poorly defined. Previous reports suggest that FTO can be modulated by several post‐translational modifications: ubiquitination and SUMOylation decrease its protein stability, while acetylation increases its demethylase activity [19, 20, 21]. However, whether FTO itself is regulated by *O*‐GlcNAcylation, a key nutrient‐sensitive modification elevated under lipid stress, has not been explored.

In this study, we explore the functional impact of lipid stress‐induced HBP activation and resultant *O*‐GlcNAcylation in MASH pathogenesis. We demonstrate that *O*‐GlcNAcylation of FTO at Ser195 enhances its protein stability and m6A demethylase activity, thereby exacerbating hepatic steatosis and inflammation. Our findings establish a critical link between nutrient‐sensing and epitranscriptomic regulation in MASH pathogenesis, and reveal FTO *O*‐GlcNAcylation as a potential therapeutic target for MASH.

## 2 Results

### 2.1 Hepatic HBP and *O*‐GlcNAcylation Are Upregulated in Murine MASH Models

To characterize metabolic alterations underlying MASH pathogenesis, we established two murine models: GAN‐diet C57BL/6 mice and liver‐specific *Pten* knockout mice (*Pten* L‐KO mice) that spontaneously develop steatohepatitis by 40 weeks (Figure 1A,B, Figure S1A–M and S2A–M) [22]. Untargeted metabolomics of GAN diet‐fed mice livers revealed distinct metabolic profiles, with lipids and lipid‐like molecules representing the predominant class (Figure S3A,B). Notably, differential metabolite analysis indicated activation of the hexosamine biosynthesis pathway (HBP), a key nutrient‐sensing pathway (Figure 1C). Consistent with this, LC–MS/MS confirmed elevated hepatic UDP‐GlcNAc, the end product of HBP, in both GAN‐fed and *Pten* L‐KO mice (Figure 1D,E). In vitro treatment with free fatty acids (FFAs) (oleic acid: palmitic acid = 2:1) also dose‐dependently increased UDP‐GlcNAc levels in both MIHA hepatocytes and primary murine hepatocytes (PMH; Figure S3C,D). Given that UDP‐GlcNAc serves as the substrate for *O*‐GlcNAcylation, we next evaluated this modification in MASH livers. Immunoblotting and immunohistochemistry showed markedly elevated global *O*‐GlcNAcylation in both models (Figure 1F–I; Figure S3E), despite unaltered *Ogt* and *Oga* mRNA expression (Figure S3F,G). Collectively, these results suggest that the hepatic HBP pathway and global protein *O*‐GlcNAcylation are upregulated in MASH mouse models and may contribute to MASH progression.

**FIGURE 1:**
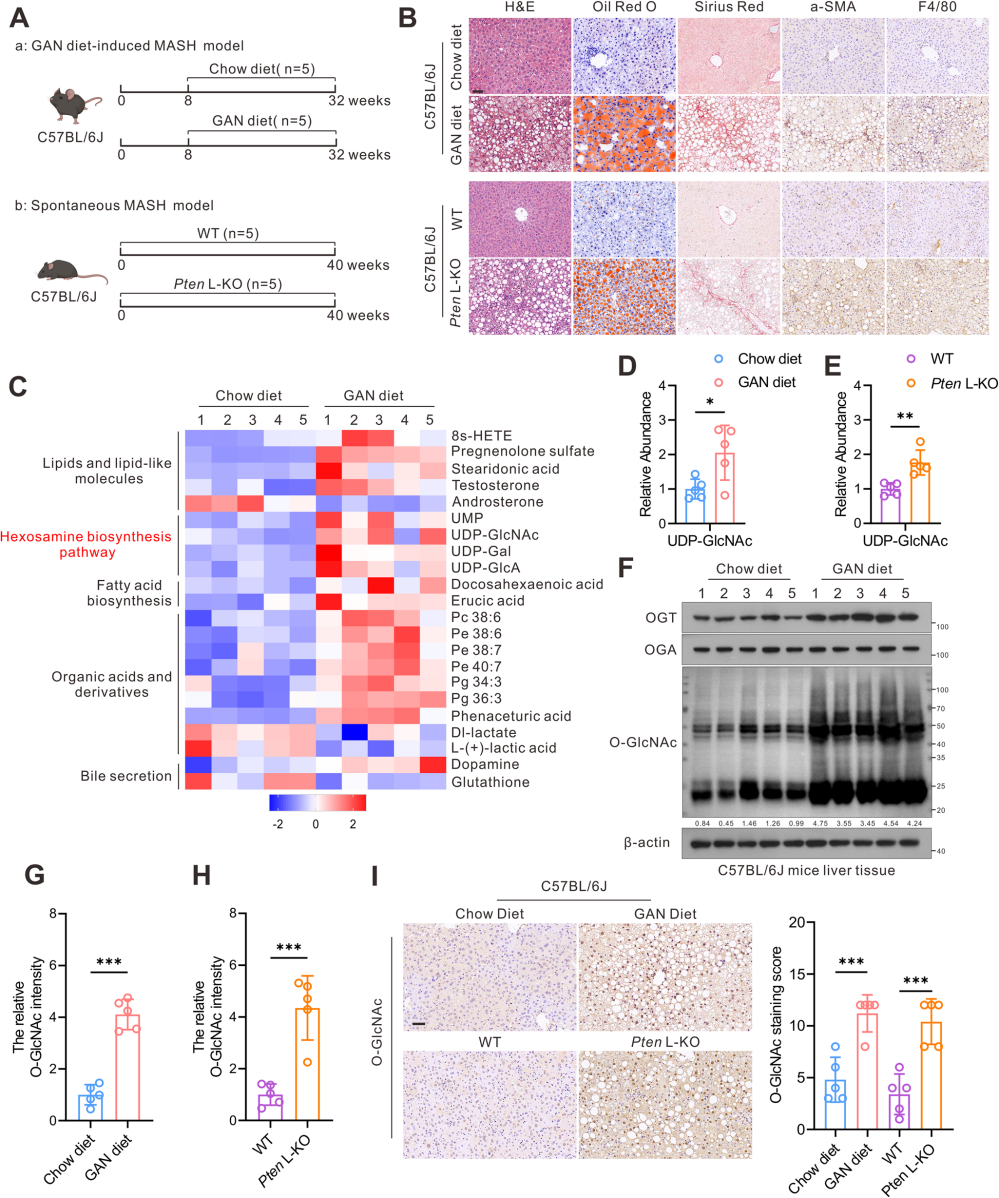
Hepatic HBP is activated in murine MASH models. (A) Schematic illustration of MASH mouse models. C57BL/6J mice fed a GAN diet (24 weeks, n = 5) and *Pten^flox/flox^*; Albumin‐Cre mice (*Pten* L‐KO mice, 40 weeks, n = 5). (B) Paraffin‐embedded serial liver sections were stained with H&E, Sirius Red, and immunostained for α‐SMA and F4/80. The frozen sections were stained with Oil Red O. Scale bar = 50 µm. (C) Heatmap of hepatic metabolite alterations in GAN diet‐fed mice. (D, E) UDP‐GlcNAc abundance in GAN diet‐fed mice (D) and *Pten* L‐KO mice (E). (F) The expression levels of OGT, OGA, and *O*‐GlcNAcylation were analyzed by immunoblotting. (G, H) Quantification of hepatic *O*‐GlcNAcylation intensity in GAN diet‐fed mice (G) and *Pten* L‐KO mice (H). (I) Representative *O*‐GlcNAcylation immunostaining and quantification in liver tissues from GAN diet‐fed mice and *Pten* L‐KO mice. Scale bar = 50 µm. For (D, E, G, H, I), statistical data are presented as mean ± SD, and statistical analyses were performed using a two‐tailed Student's t‐test. ^*^ *p* < 0.05, ^**^ *p* < 0.01 and ^***^ *p* < 0.001.

### 2.2 Proteomic Identification of FTO Ser195 as the Primary *O*‐GlcNAcylation Site

To delineate the functional impact of hyper‑*O*‑GlcNAcylation in MASH pathogenesis, we performed 4D‐label‐free quantitative *O*‐GlcNAcylation proteomics (Figure 2A). Subcellular localization analysis revealed that *O*‐GlcNAcylation primarily occurs in the nucleus (Figure S4A). Additionally, gene ontology (GO) and KEGG enrichment analysis demonstrated that *O*‐GlcNAcylated proteins were enriched in cellular processes, catalytic activity, PI3K‐AKT and calcium signaling pathway, suggesting a crucial role in MASH progression (Figure S4B,C). To identify key molecules, we conducted intersection analysis of mass spectrometry data (*O*‐GlcNAc‐modified proteins upregulated fold change > 5) and GWAS‐identified MASH‐associated genes [23]. This integrative screen highlighted FTO as a central node, displaying a 7.62‐fold increase in *O*‐GlcNAc modification at Ser192 (Mus musculus residue; equivalent to human Ser195) in GAN diet‐induced MASH mice (Figures 2B,C). Immunohistochemistry (IHC) and sWGA pull‐down assays confirmed elevated FTO protein and *O*‑GlcNAcylation levels in murine MASH models (Figure 2D and S4D). Co‐IP in MIHA and primary murine hepatocytes (PMH) confirmed a direct FTO‐OGT interaction (Figures 2E–G and S5A), and immunofluorescence revealed their nuclear co‐localization (Figures 2H and S5B). Using two constructed FTO deletion mutants (Figure 2I), we found that the N‐terminal region of FTO (1‐326 aa) is necessary for interaction with OGT (Figure 2J). Moreover, treatment with Thiamet G (TMG, an OGA inhibitor) or OSMI‐1 (an OGT inhibitor) significantly increased or decreased FTO *O*‐GlcNAcylation, respectively (Figure S5C–J). Subsequent analysis of FTO mass spectrometry data (Figure 2K) and cross‐species conservation analysis confirmed high conservation of human FTO Ser195 across multiple species (Figure 2L). Critically, a serine‑to‑alanine mutation (S195A) markedly abolished FTO *O*‐GlcNAcylation in sWGA pull‐down, *O*‐GlcNAc IP, and Click‐iT assays (Figures 2M,N and S5K), confirming Ser195 as the primary *O*‐GlcNAcylation site of FTO.

**FIGURE 2:**
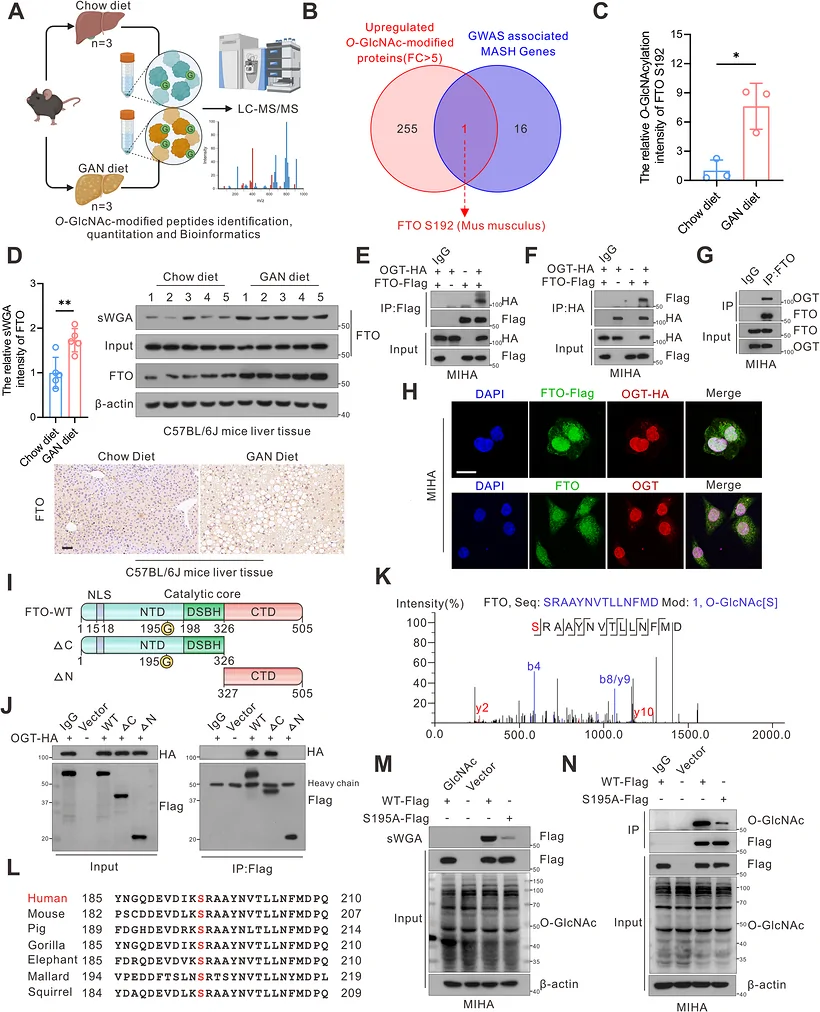
Ser195 of FTO is the major *O*‐GlcNAcylation site. (A) Schematic diagram of *O*‐GlcNAcylation 4D label‐free quantitative proteomics. (B) Venn diagram of upregulated *O*‐GlcNAc‐modified proteins (fold change > 5) and GWAS‐associated MASH genes. (C) The *O*‐GlcNAcylation intensity of FTO Ser192 (Mus musculus: S192, Homo sapiens: S195) in proteomic analysis. (D) Hepatic FTO protein levels and FTO *O*‐GlcNAcylation intensity in GAN diet‐fed mice: immunoblot (top), IHC (bottom), and sWGA pull‐down (top; quantified left), scale bar = 50 µm. (E‐G) The Co‐IP of exogenous (E, F) and endogenous (G) OGT and FTO in MIHA cells. (H) Subcellular colocalization of exogenous (up) and endogenous (down) OGT and FTO in MIHA cells via immunofluorescence, scale bar = 20 µm. (I) Schematic of truncated FTO (△C or △N) construction. NTD: N‐terminal region of FTO (△C, 1–326 aa). CTD: C‐terminal region of FTO (△N, 327–505 aa). DSBH: double‐stranded β‐helix, which serves as the catalytic core of FTO. G: *O*‐GlcNAcylation. (J) The interactions between OGT and wild‐type FTO or truncated FTO (△C or △N) were detected in MIHA cells via Co‐IP. (K) Proteomic LC‐MS identified FTO Ser192 as the *O*‐GlcNAcylation site. (L) Cross‐species protein sequence alignment of FTO. (M, N) MIHA cells were transfected with Flag‐tagged FTO (WT, S195A) plasmids. sWGA pull‐down assay (M) and Co‐IP (N) were performed to detect the *O*‐GlcNAcylation signal. For (C, D), statistical data are presented as mean ± SD, and statistical analyses were performed using a two‐tailed Student's t‐test. ^*^ *p* < 0.05 and ^**^ *p* < 0.01.

### 2.3 *O*‐GlcNAcylation Enhances FTO Activity by Increasing Protein Stability

*O*‐GlcNAcylation dynamically modulates protein stability, enzymatic activity, and subcellular localization [24, 25]. To validate its functional impact on FTO, we treated MIHA and PMH cells with TMG and OSMI‐1, and evaluated protein stability. Cycloheximide chase assays showed TMG prolonged FTO half‐life, whereas OSMI‐1 accelerated its degradation (Figure S6A,B). Consistently, the S195A mutation and shRNA‐mediated OGT knockdown also accelerated FTO degradation (Figure 3A, Figure S6C). Crucially, only the proteasomal inhibitor MG132, but not lysosomal inhibitors [chloroquine (CQ); bafilomycin A1 (BafA1)], rescued OSMI‐1‐induced FTO degradation (Figure 3B), indicating ubiquitin‐proteasome‐dependent regulation. Ubiquitination assays further demonstrated that TMG reduced FTO polyubiquitination, whereas OSMI‐1 increased it; this effect was abolished by the S195A mutation (Figure 3C, Figure S6D). To identify the specific E3 ubiquitin ligases or deubiquitinases involved, we screened literature‐reported candidates [20, 26, 27, 28, 29] and identified USP20 (a deubiquitinase) and FBXW7 (an E3 ligase) as key interactors (Figure S6E). USP20 overexpression elevated FTO abundance, while FBXW7 overexpression reduced it (Figure S6F,G). Co‐IP assays revealed that wild‐type FTO bound more strongly to USP20 and weakly to FBXW7 than the S195A mutant (Figure 3D,E), indicating that *O*‐GlcNAcylation stabilizes FTO by enhancing USP20 recruitment and attenuating FBXW7‐mediated ubiquitination.

**FIGURE 3:**
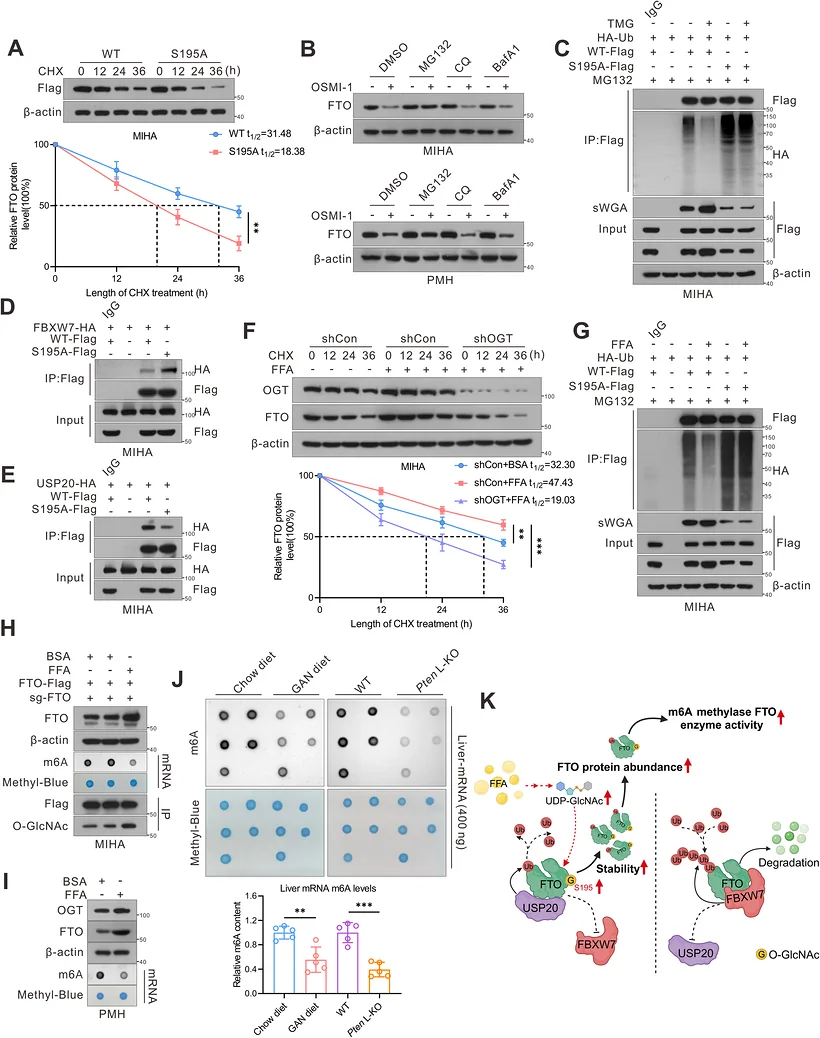
FFA enhanced hepatic FTO enzymatic activity in an *O*‐GlcNAcylation‐dependent manner. (A) MIHA cells were transfected with Flag‐tagged FTO (FTO‐WT, FTO‐S195A) plasmids, then cells were treated with 300 µm cycloheximide (CHX) for the indicated time (n = 3, performed in triplicate). For each group, the blotting signals were quantified by using ImageJ and normalized against the 0 h time point. (B) MIHA (top) or PMH (bottom) cells were treated with 10 µm MG132, 50 µm chloroquine (CQ), or 100 nm bafilomycin A1 (BafA1) for 6 h to block OSMI‐1‐induced FTO degradation. (C) MIHA cells were transfected with HA‐Ub and Flag‐tagged FTO plasmids and treated with DMSO, 25 µm Thiamet G (TMG), or 40 µm OSMI‐1 for 12 h. Before being harvested, cells were treated with 15 µM MG132 for 6 h and were divided into two parts for sWGA pull‐down and FTO ubiquitination assay. (D, E) Co‐IP of Flag‐tagged FTO with USP20‐HA (D) or FBXW7‐HA (E) in MIHA cells. (F) MIHA cells were infected with shCon or shOGT viruses and cultured with 200 µm FFA or BSA (n = 3, performed in triplicate). (G) Ubiquitination of Flag‐tagged FTO in MIHA cells cultured with 200 µm FFA. (H) MIHA cells were infected with FTO‐sg lentiviral viruses to induce endogenous FTO knockdown and transfected with FTO‐WT plasmids to overexpress exogenous FTO, followed by cultured with 200 µm FFA or BSA for 24 h. Immunoblotting and m6A dot blot were performed. (I) PMH cells were cultured with 200 µm FFA or BSA for 24 h and harvested for immunoblotting and m6A dot blot. (J) Hepatic mRNA m6A levels in MASH mice (top) and the quantitative analysis, n = 5 (bottom). (K) Schematic illustration of FFA upregulating *O*‐GlcNAcylation of FTO at Ser195, impeding FBXW7‐dependent ubiquitination, prolonging FTO half‐life, and enhancing enzyme activity. For (A, F, J), statistical data are presented as mean ± SD. For (A, J), statistical analyses were performed using a two‐tailed Student's *t*‐test. For (F), statistical analysis was performed using one‐way ANOVA followed by Tukey's test. ^**^ *p* < 0.01 and ^***^ *p* < 0.001.

We next examined whether *O*‑GlcNAcylation influences FTO's m6A demethylase activity, which depends on both protein abundance and catalytic efficiency (Figure S7A). TMG reduced global m6A levels without altering intrinsic FTO catalytic efficiency, whereas OSMI‐1 exerted the opposite effect (Figure S7B,C). When protein levels were normalized among FTO‐WT, the S195A mutant, and a catalytically inactive mutant (H231A, D233A, R316Q, R322Q), FTO‐WT and the S195A mutant showed comparable global m6A levels (Figure S7D). In vitro FTO demethylation assays and RNA pull‐down assays confirmed similar catalytic efficiency between FTO‐WT and the S195A mutant (Figure S7E,F). These results indicate that *O*‐GlcNAcylation enhances FTO enzymatic activity primarily by increasing its protein stability rather than altering its intrinsic catalytic efficiency.

### 2.4 Free Fatty Acids Enhance FTO Demethylase Activity in an *O*‐GlcNAcylation‐Dependent Manner

Our proteomics data revealed elevated FTO *O*‐GlcNAcylation in murine MASH models (Figure 2C). To model hepatic lipid stress in vitro, we treated MIHA and PMH cells with escalating concentrations of FFA (Figure S8A,D). FFA treatment elevated FTO protein and global *O*‑GlcNAcylation in a dose‑dependent manner without affecting *FTO* mRNA levels (Figure S8B,E). Lipid accumulation and enhanced FTO *O*‑GlcNAcylation were confirmed by Oil Red O staining and sWGA pull‑down, respectively (Figure S8C,F), confirming that lipid overload upregulates FTO *O*‐GlcNAcylation.

To investigate whether FFA enhances FTO enzymatic activity in an *O*‐GlcNAcylation‐dependent manner, we performed FTO protein stability and ubiquitination assays in MIHA cells. Under FFA stimulation, lipid overload reduced ubiquitination and extended the half‑life of wild‑type FTO, but not of the S195A mutant; these effects were abolished by OGT knockdown (Figure 3F,G). Consistently, FFA‐stimulated cells and MASH livers exhibited increased FTO abundance and reduced global m6A levels (Figure 3H–J). Collectively, these results demonstrated that lipid stress enhances FTO *O*‐GlcNAcylation, inhibits ubiquitin‐proteasomal degradation, increases protein stability and abundance, and ultimately elevates FTO enzymatic activity and suppresses hepatic m6A levels (Figure 3K).

### 2.5 RNA‐seq and MeRIP‐seq Identify *HRAS* as a Key FTO Target Under Lipid Stress

Given that lipid overload enhances FTO protein abundance and demethylase activity via *O*‐GlcNAcylation, we sought to identify its functional mRNA targets under lipid stress. We performed integrated RNA sequencing (RNA‐seq) and m6A RNA immunoprecipitation sequencing (MeRIP‐seq) in FTO‐knockout (sg‐FTO) and control (sg‐control) MIHA cells under FFA stimulation (Figure 4A). Global m6A levels were significantly elevated in sg‐FTO cells, as confirmed by m6A dot blot and cumulative distribution analyses (Figure 4B,C). m6A peaks were enriched near stop codon‐proximal regions (stopC) and coding sequences (CDS) (Figure 4D), and m6A motif analysis confirmed enrichment of the canonical RRACH (R = A/G; H = A/C/U) sequence (Figure S9A). Integration of differentially expressed genes (DEGs) from RNA‐seq and differentially m6A‐modified transcripts from MeRIP‐seq identified 87 overlapping candidate target genes (Figure 4E). KEGG and GO analyses of these genes highlighted significant enrichment of the MAPK signaling pathway (Figure 4F, Figure S9B). Gene Set Enrichment Analysis (GSEA) further indicated downregulation of MAPK signaling, TNF‐α response, inflammation, and cytokine activity in sg‐FTO cells (Figure S9C), implicating a critical role of FTO in lipotoxicity and MASH pathogenesis. Among MAPK pathway‐enriched genes validated by RT‐qPCR, *HRAS* exhibited the most pronounced reduction in sg‐FTO cells, while re‐expression of wild‐type FTO, but not the S195A mutant, restored *HRAS* mRNA levels (Figure 4G, Figure S9D). MeRIP‐qPCR and RIP‐qPCR confirmed that FTO binds to *HRAS* mRNA and reduces its m6A modification (Figure 4H,I, Figure S9E,F). IGV genome browser analysis identified a specific m6A peak within exon 1 of *HRAS* (Figure 4J). Using synthesized *HRAS*‐specific oligos based on this site, in vitro demethylation and RNA pull‐down assays demonstrated that FTO directly binds to *HRAS* mRNA and demethylates this specific m6A site (Figure 4K,L, Figure S9G). Collectively, these findings demonstrate that lipid stress promotes FTO‐mediated m6A demethylation of *HRAS* mRNA, establishing *HRAS* as a key functional target of FTO.

**FIGURE 4:**
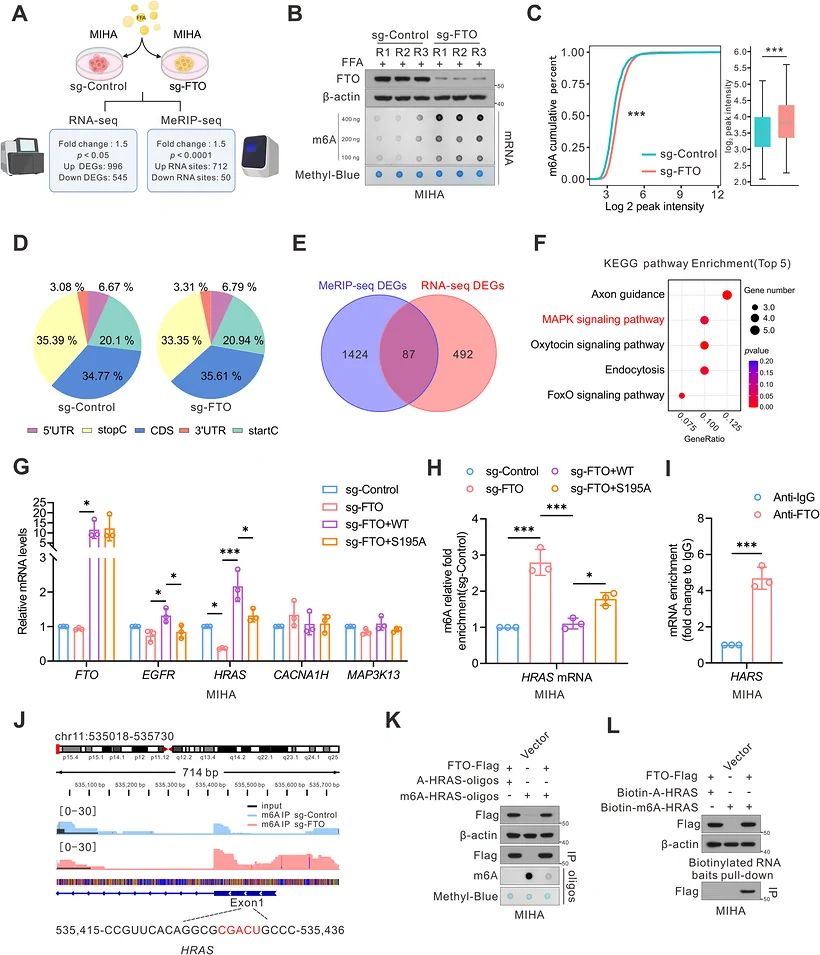
Identification of FTO targets by high‐throughput RNA‐seq and MeRIP‐seq. (A) Schematic of RNA‐seq and MeRIP‐seq (n = 3) workflow. (B) Immunoblotting and m6A dot blot of RNA‐seq and MeRIP‐seq cell samples. (C) Cumulative distribution curves and box plot for m6A peak abundance in the sg‐Control and sg‐FTO groups. (D) Pie chart depicting the distribution of m6A peaks. (E) Venn diagram illustrating the overlapped genes of RNA‐seq and MeRIP‐seq. (F) Enrichment of KEGG pathways of overlapped genes. (G) Relative mRNA levels of MAPK signaling pathway‐enriched genes in MIHA cells identified by RT‐qPCR (n = 3, performed in triplicate). (H) MeRIP‐qPCR to detect m6A intensity of *HRAS* mRNA in MIHA cells. (I) RIP‐qPCR using anti‐FTO antibody in MIHA cells. (J) Different *HRAS* m6A peaks in sg‐Control and sg‐FTO cells were visualized using IGV. (K) Immunopurified FTO‐Flag protein was incubated with A or m6A *HRAS* oligos (in vitro FTO demethylation activity assay), and the m6A level was determined. (L) RNA pull‐down in MIHA cells. For (C, G, H, I), statistical data are presented as mean ± SD. For (C), statistical analysis was performed using the Wilcoxon rank‐sum test (Mann‐Whitney U test) by Cloud‐seq Biotech (Shanghai, China). For (G, H), statistical analyses were performed using one‐way ANOVA followed by Tukey's test. For (I), statistical analysis was performed using a two‐tailed Student's t‐test. ^*^ *p* < 0.05 and ^***^ *p* < 0.001.

### 2.6 FTO *O*‐GlcNAcylation Accelerates Hepatic Lipid Accumulation via the HRAS/PI3K/AKT Axis

To define the functional consequence of FTO‑mediated *HRAS* regulation, we first assessed *HRAS* mRNA stability. These results demonstrated that wild‐type FTO, but not the S195A mutant, significantly prolonged *HRAS* mRNA half‐life (Figure 5A). Treatment with the FTO enzymatic inhibitor FB23‐2 or re‐expression of a catalytically inactive FTO mutant reversed this stabilizing effect, indicating that FTO *O*‐GlcNAcylation upregulates *HRAS* mRNA levels in an m6A demethylase activity‐dependent manner (Figure 5B). m6A modifications are recognized by distinct reader proteins (e.g., YTHDF1/2/3, IGF2BP1/2/3) that regulate mRNA stability, translation, splicing, and nuclear export [30, 31]. To identify which m6A reader proteins bind to *HRAS* mRNA, we performed RNA pull‐down assays using *HRAS*‐specific probes, which revealed significant enrichment of YTHDF2 (Figure 5C, Figure S10A). Subsequent RIP‐qPCR and mRNA stability assays confirmed that YTHDF2 binds to *HRAS* mRNA and destabilizes it (Figure 5D,E, Figure S10B). Western blotting further demonstrated that YTHDF2 decreases HRAS protein abundance (Figure 5F). Given that HRAS activates both the RAF/MEK/ERK and PI3K/AKT signaling pathways in MASH progression [32, 33, 34], we conducted rescue experiments in MIHA and PMH cells. Re‐expression of FTO‐WT, but not the S195A mutant, preferentially activated the HRAS/PI3K/AKT pathway over the RAF/MEK/ERK pathway (Figure 5G, Figure S10C). Moreover, *HRAS* knockdown significantly reduced intracellular TC, TG, and lipid accumulation, while re‐expression of HRAS in sg‐FTO cells robustly activated the PI3K/AKT pathway and restored TC, TG levels, and lipid accumulation (Figure 5H, Figure S10D–F). Conversely, AKT inhibition with MK2206 suppressed FTO‑driven lipid accumulation (Figure 5I–K, Figure S10G, H).

**FIGURE 5:**
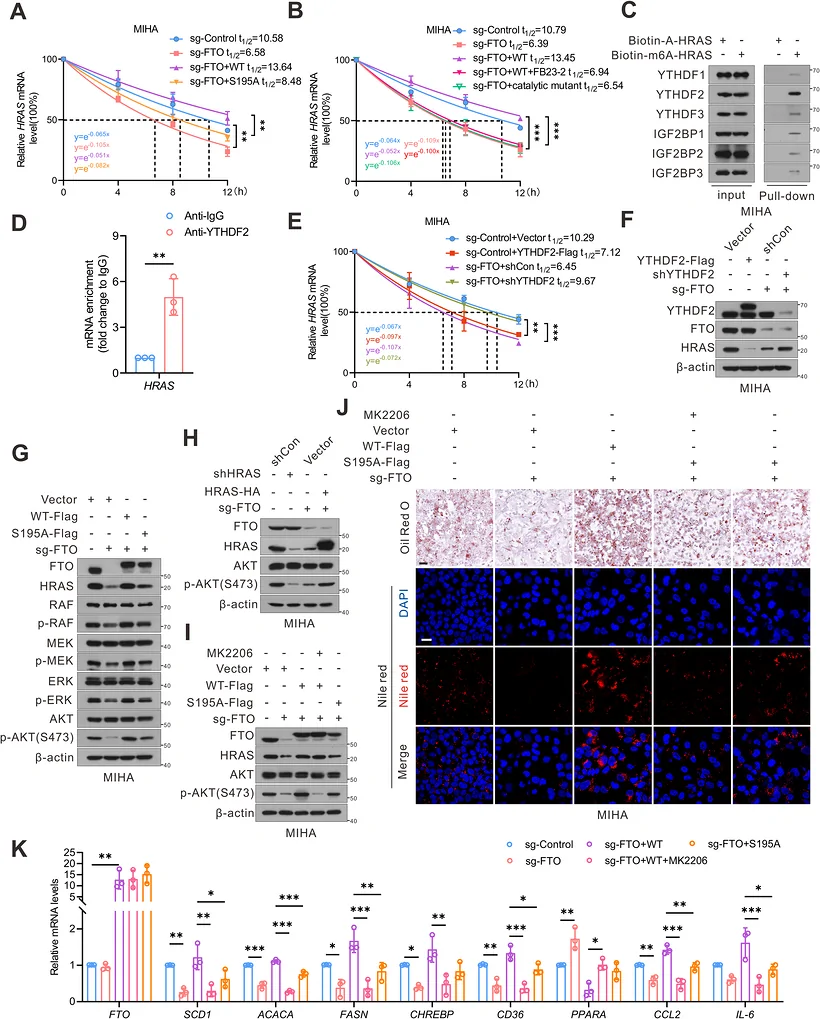
FTO stabilizes *HRAS* mRNA via m6A demethylase activity, activating the PI3K/AKT axis to drive intracellular lipid accumulation. (A) The half‑life of *HRAS* mRNA in MIHA cells. Cells were infected with sg‐Control or sg‐FTO viruses and transfected with Flag‐tagged FTO or vector plasmids. All cells were treated with actinomycin D (5 µg/mL) for the indicated time (n = 3, performed in triplicate). Within each group, the mRNA abundance at the 0 h time point was set to 100%. (B) After viral infection, MIHA cells were transfected with FTO‐Flag or the catalytically inactive mutant (FTO catalytic mutant; H231A, D233A, R316Q, R322Q) and cultured with FB23‐2 (FTO demethylase inhibitor, 10 µm) or DMSO for 24 h. (C) Immunoblotting detecting the enrichment of m6A readers (YTHDF1/2/3, IGF2BP1/2/3) in an RNA pull‐down assay using A or m6A *HRAS* probes in MIHA cells. (D) RIP‐qPCR using anti‐YTHDF2 antibody in MIHA cells, and fold enrichment was normalized to the IgG control. (E, F) MIHA sg‐Control cells were transfected with the vector or YTHDF2‐Flag plasmids, while MIHA sg‐FTO cells were infected with shCon or shYTHDF2 viruses. The half‐life of *HRAS* mRNA (E) and HRAS protein abundance (F) were detected using RT‐qPCR and immunoblotting. (G, H) MIHA cells were infected with sg‐Control or sg‐FTO viruses and transfected with Flag‐tagged FTO (G) or HRAS‐HA (H) plasmids. Protein abundance was detected using immunoblotting. (I‐K) After viral infection and Flag‐tagged FTO plasmid transfection, MIHA cells were cultured with DMSO or 10 µM MK2206 (AKT inhibitor) for 24 h. ORO (J, top, scale bar = 50 µm) and Nile Red (J, bottom, scale bar = 20 µm) staining of intracellular lipid droplets. Relative mRNA levels of lipogenic and inflammatory genes (K) in MIHA cells were identified by RT‐qPCR (n = 3, performed in triplicate). For (A, B, D, E, K), statistical data are presented as mean ± SD. All statistical analyses except (D) were performed using one‐way ANOVA followed by Tukey's test. For (D), statistical analysis was performed using a two‐tailed Student's *t*‐test. ^*^ *p* < 0.05, ^**^ *p* < 0.01 and ^***^ *p* < 0.001.

To validate these findings in vivo, we employed a GAN diet‐induced MASH model subjected to hepatic *Fto* knockdown via AAV8‐TBG‐sh*Fto* (5 × 10^11^ V.g), followed by adenoviral rescue with either pAd‐hFTO‐WT‐Flag or pAd‐hFTO‐S195A‐Flag (2×10^9^ pfu), along with administration of MK2206 (Figure 6A). *Fto* knockdown markedly ameliorated MASH pathology. Compared with the S195A mutant, re‐expression of hFTO‐WT resulted in larger livers, increased body weight and liver‐to‐body weight ratios, elevated serum AST, ALT, TC, TG and FFA levels, as well as higher hepatic TNF‐α and IL‐6 levels (Figure 6B–G, Figure S11A–H). Furthermore, wild‐type FTO rescue also led to higher hepatic FTO abundance, more pronounced activation of the HRAS/PI3K/AKT pathway, increased lipid accumulation, and inflammatory infiltration (Figure 6H–L, Figures S11I,J and S12A,B). Critically, these effects were partially reversed by MK2206 treatment. Collectively, these findings demonstrate that lipid stress‐induced FTO *O*‐GlcNAcylation accelerates hepatic lipid accumulation and promotes MASH progression through activation of the HRAS/PI3K/AKT signaling axis.

**FIGURE 6:**
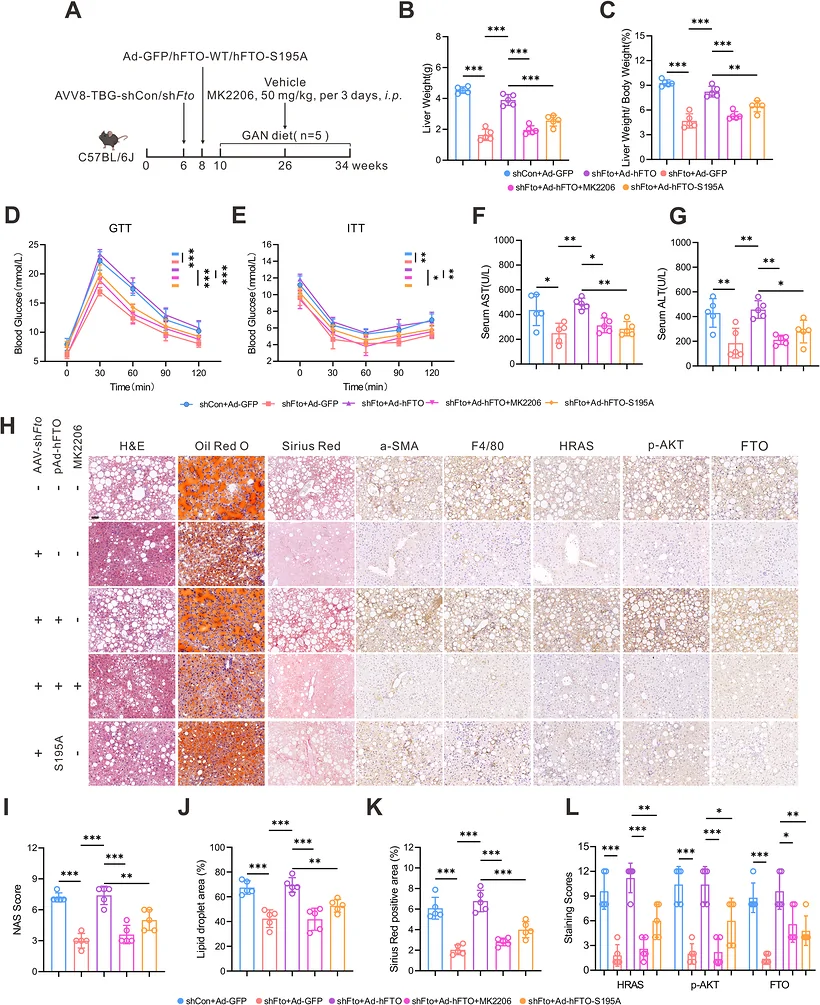
Targeting FTO *O*‐GlcNAcylation and AKT alleviates MASH in vivo. (A) Schematic diagram of GAN diet‐induced MASH mice. (B, C) The liver weights (B) and the ratio of liver weight to body weight (C). (D‐G) The GTT (D), ITT (E), serum AST (F), and ALT (G) concentrations. (H) Paraffin‐embedded serial liver sections were stained with H&E, Sirius Red, and immunostained for α‐SMA, F4/80, HRAS, p‐AKT, and FTO. The frozen sections were stained with Oil Red O. Scale bar = 50 µm. (I–L) The quantitative and semi‐quantitative analyses of immunohistochemistry data. NAFLD activity score (NAS). For (D‐G, I‐L), statistical data are presented as mean ± SD and performed using one‐way ANOVA followed by Tukey's test. ^*^ *p* < 0.05, ^**^ *p* < 0.01 and ^***^ *p* < 0.001.

### 2.7 Targeting FTO Ser195 *O*‐GlcNAcylation Alleviates MASH Progression

Cell‐penetrating peptides (CPPs), short peptides capable of penetrating cell membranes, have been extensively engineered for specific molecular targeting [35, 36]. To specifically target FTO Ser195 *O*‐GlcNAcylation, we designed a series of CPPs (Figure 7A) and generated a site‐specific antibody against FTO Ser195 *O*‐GlcNAcylation (S195‐O‐FTO). Our in vitro results showed that CPPs‐S195‐3# potently suppressed FTO Ser195 *O*‐GlcNAcylation in both MIHA and PMH cells with no significant cytotoxicity (Figure 7B,C, Figure S13A,B). To evaluate its cellular uptake efficiency and in vivo biodistribution, we conjugated the CPPs with the CY5 fluorophore (Figure S13C). Time‐course imaging of MIHA and PMH cells incubated with 20 µm CY5‐CPPs revealed efficient cellular uptake in a time‐dependent manner (Figure S13D,E). Following intraperitoneal injection of CY5‐CPPs (5 mg/kg) into C57BL/6J mice, in vivo and *ex vivo* organ imaging at 12 h post‑injection revealed predominant fluorescence enrichment in the liver (Figure S13F). Functionally, CPPs‑S195‑3# suppressed the activation of the HRAS/PI3K/AKT pathway and reduced intracellular TC, TG, and lipid accumulation under lipid overload conditions (Figure 7D–G, Figure S14A–D).

**FIGURE 7:**
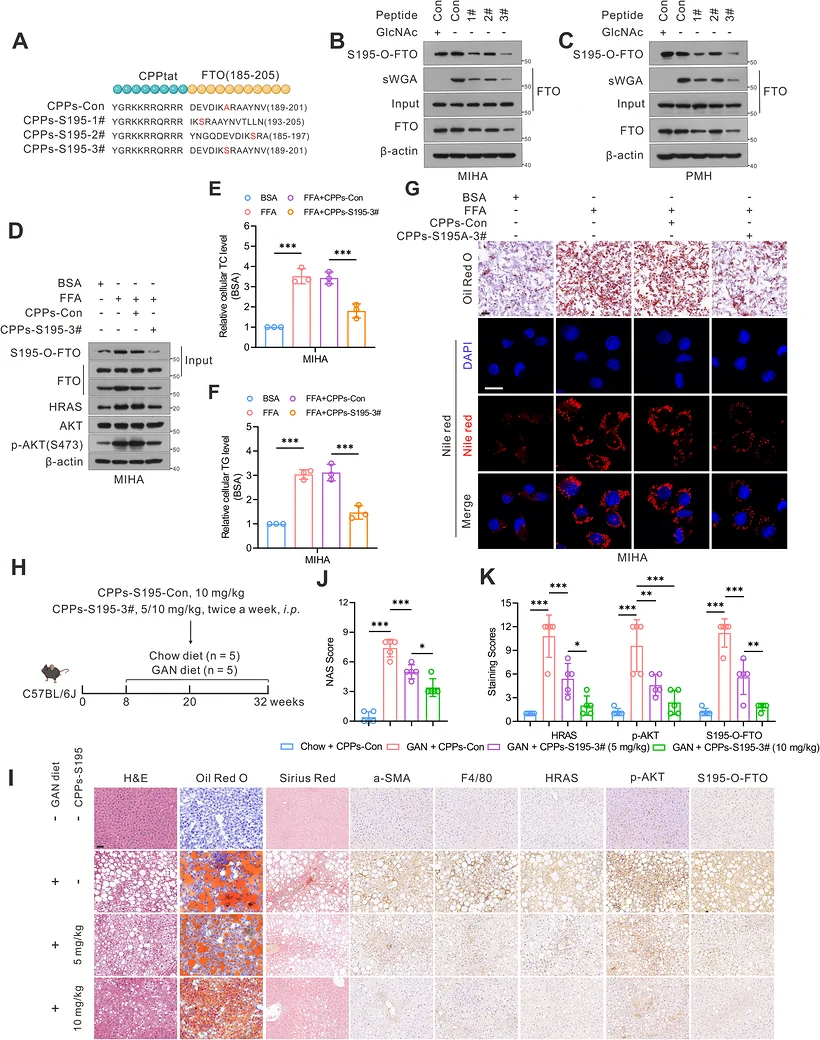
CPPs‐Mediated Inhibition of FTO Ser195 *O*‐GlcNAcylation suppresses the HRAS/PI3K/AKT axis and alleviates MASH in vitro and in vivo. (A) Design of FTO cell‐penetrating peptides (CPPs). (B, C) FTO *O*‐GlcNAcylation levels in MIHA (B) and PMH (C) cells treated with CPPs (20 µM, 24 h) were assessed by sWGA pull‐down and S195‐O‐FTO antibody (FTO Ser195‐specific O‐GlcNAc antibody). (D‐G) MIHA cells were pretreated with 200 µm FFA or BSA control, followed by CPPs‐S195‐3# or CPPs‐Con (20 µM). FTO *O*‐GlcNAcylation was analyzed using immunoblotting (D). Total cholesterol (E) and triglyceride (F) levels were measured and normalized to the BSA control group. Lipid droplets were visualized by Oil Red O (top, scale bar = 50 µm) and Nile Red (bottom, scale bar = 20 µm) staining (G). (H) Treatment regimen for CPPs administration in GAN diet‐induced MASH mice. (I) Paraffin‐embedded serial liver sections were stained with H&E, Sirius Red, and immunostained for α‐SMA, F4/80, HRAS, p‐AKT, and S195‐O‐FTO. The frozen sections were stained with Oil Red O. Scale bar = 50 µm. (J, K) The semi‐quantitative analyses of immunohistochemistry data. For (E, F, J, K), statistical data are presented as mean ± SD and performed using one‐way ANOVA followed by Tukey's test. ^*^ *p* < 0.05, ^**^ *p* < 0.01 and ^***^ *p* < 0.001.

To validate these findings in vivo, GAN‐diet‐induced MASH mice were treated with CPPs‐S195‐3# for 12 weeks (Figure 7H). Compared to the CPPs‐Con group, CPPs‐S195‐3# treatment significantly attenuated GAN diet‐induced MASH pathology. This protection manifested as reduced body weight, decreased liver size and weight, lower liver‐to‐body weight ratios, improved serum markers (AST, ALT, TC, TG, FFA), diminished hepatic lipid accumulation, and reduced inflammatory infiltration (Figure 7I–K, Figures S15A–R and S16A). Crucially, CPPs‐S195‐3# dose‐dependently reduced FTO S195 *O*‐GlcNAcylation and suppressed the HRAS/PI3K/AKT pathway, while exhibiting a favorable in vivo safety profile (Figure S16B–G). Collectively, these results demonstrated that targeted inhibition of FTO *O*‑GlcNAcylation at Ser195 via CPPs‑S195‑3# suppresses the HRAS/PI3K/AKT axis and alleviates MASH progression.

### 2.8 The FTO/HRAS/PI3K/AKT Axis Is Activated in Human MASH Livers

We investigated whether the FTO/HRAS/PI3K/AKT axis is also active in MASH patients. Analysis of human MASH datasets (GSE37031, GSE130970) revealed unchanged *FTO* mRNA but significantly elevated *HRAS* expression in MASH livers (Figure 8A, Figure S17A). GSEA analysis further indicated activation of the PI3K/AKT and MAPK signaling pathways in MASH patients (Figure 8B, Figure S17B). Immunohistochemistry on serial liver sections from MASH patients (n = 15, Clinical information in Table S3) and healthy donors (n = 6) confirmed increased FTO S195 *O*‑GlcNAcylation, higher FTO protein abundance, and enhanced HRAS/PI3K/AKT signaling in MASH samples (Figure 8C,D). Furthermore, correlation analysis of IHC staining scores showed significant positive correlations between FTO S195 *O*‐GlcNAcylation, FTO, HRAS, and p‐AKT levels (Figure 8E, Figure S17C,D). Taken together, these findings confirmed the activation of the FTO/HRAS/PI3K/AKT axis in MASH patients, underscoring its clinical relevance (Figure 8F).

**FIGURE 8:**
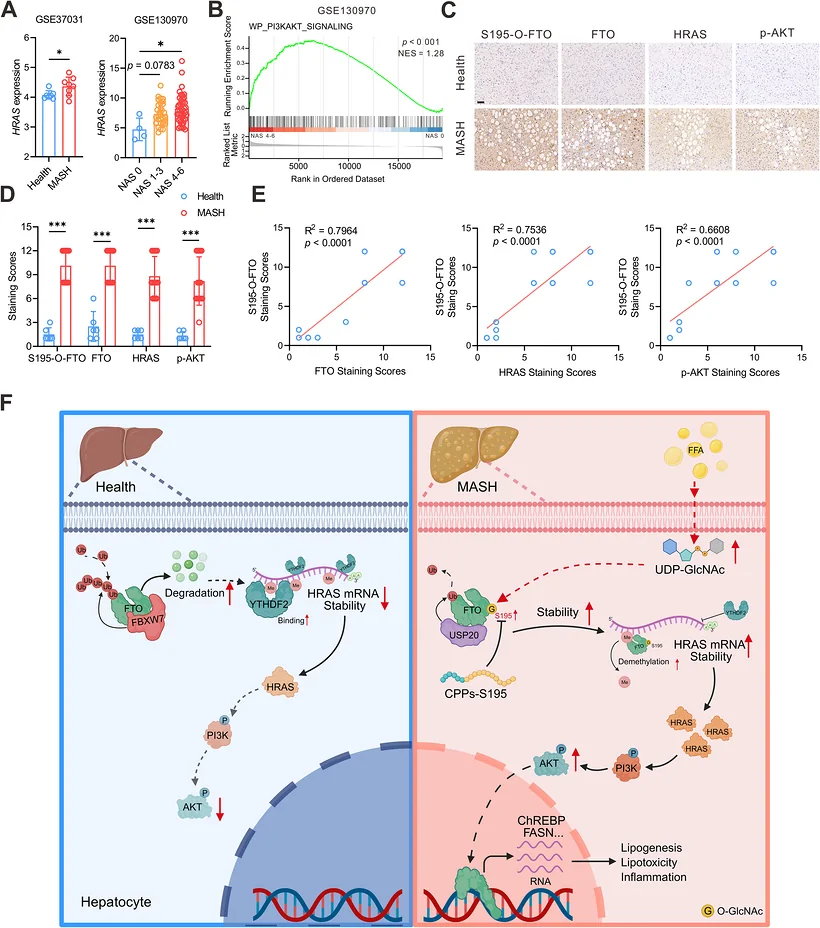
The FTO/HRAS/PI3K/AKT signaling axis is significantly upregulated in clinical specimens. (A) *HRAS* mRNA expression levels in GEO datasets. (B) GSEA analysis in GSE130970. (C) Paraffin‐embedded serial liver sections from healthy donors (n = 6) and MASH (n = 15) patients were subjected to immunohistochemical staining for S195‐O‐FTO, FTO, HRAS, and p‐AKT. Scale bar = 50 µm. (D) The semi‐quantitative analyses of immunohistochemical data. (E) Correlation analysis of S195‐O‐FTO, FTO, HRAS, and p‐AKT protein expression using the staining scores derived from (D). (F) Schematic depiction of the mechanism underlying *O*‐GlcNAcylation of FTO‐mediated MASH progression by stabilizing m6A‐modified *HRAS*. This schematic depiction was created in BioRender. Yin, J. (2025); https://BioRender.com/2xtjrou. For (D), statistical data are presented as mean ± SD; statistical analysis was performed using a two‐tailed Student's *t*‐test. ^*^ *p* < 0.05 and ^***^ *p* < 0.001.

## 3 Discussion

While lipid stress is a well‐established driver and hallmark feature of MASH [1, 37], the precise mechanisms that convert excess hepatic lipids into epitranscriptomic reprogramming via the m6A machinery remain incompletely understood. In this study, we demonstrate that lipid stress enhances UDP‐GlcNAc availability and elevates global *O*‐GlcNAcylation levels. Critically, we identify Ser195 as a novel *O*‐GlcNAcylated site on FTO that enhances its protein stability and activates the HRAS/PI3K/AKT pathway, thereby accelerating MASH progression (Figure 8F). Notably, pharmacological disruption of FTO *O*‐GlcNAcylation with CPPs‐S195‐3# ameliorates MASH pathology both in vitro and in vivo, highlighting the translational potential of targeting this PTM.

Our findings provide new insights into how lipid stress augments global *O*‐GlcNAcylation through enhanced HBP flux in MASH. While previous studies attributed increased *O*‐GlcNAcylation in MASH to elevated OGT expression [11, 12], we now show that amplified HBP flux and consequent UDP‐GlcNAc accumulation are sufficient to drive hyper‐*O*‐GlcNAcylation. Thus, both OGT expression and substrate availability coordinately regulate the hepatic *O*‐GlcNAc landscape in MASH. The HBP integrates nutrient inputs from glucose, glutamine, and acetyl‐CoA to generate UDP‐GlcNAc, thereby linking cellular metabolic status to diverse biological processes [38, 39]. We propose that lipid stress disrupts fatty acid homeostasis, which in turn stimulates HBP flux and elevates UDP‐GlcNAc availability. Importantly, unlike previous studies that primarily emphasized the role of OGT expression in promoting *O*‑GlcNAcylation [11, 12], our work reveals that enhanced HBP flux serves as a complementary mechanism under lipid overload conditions. These findings not only advance a more integrated understanding of *O*‑GlcNAc dysregulation in MASH but also highlight key HBP enzymes—such as GFAT1 (*de novo* synthesis), NAGK (salvage pathway), and PGM3—as potential therapeutic targets warranting further investigation.

As a dynamic PTM, *O*‐GlcNAcylation remodels protein function in response to metabolic stimulation, thereby influencing a range of pathological processes [24, 40]. In this study, we not only identified Ser195 as the principal *O*‐GlcNAcylation site on FTO under lipid stress, but also elucidated its functional relevance in MASH. Although *O*‐GlcNAcylation of FTO has been reported in acute myeloid leukemia (AML) and lipopolysaccharide (LPS)‐induced sepsis [41, 42], the modified residues and downstream consequences appear to be disease‐specific. Here, we demonstrated that lipid stress‐induced *O*‐GlcNAcylation at Ser195 impedes FBXW7‐mediated ubiquitination, thereby enhancing FTO protein stability and activating the HRAS/PI3K/AKT signaling axis via its m6A demethylase activity. This mechanism provides a plausible explanation for the observed elevation of FTO and the concomitant global loss of m6A modification in MASH models [15, 18], thereby positioning FTO *O*‐GlcNAcylation as a crucial link between metabolic stress and epitranscriptomic reprogramming.

Notably, m6A modification is the most abundant epigenetic modification in mammalian mRNAs that regulates RNA stability, splicing, export, and translation [43, 44], with growing implications in MASH pathogenesis [45, 46]. Our integrated RNA‐seq and MeRIP‐seq analyses identify *HRAS* as a key target of FTO under lipid stress, revealing a previously unrecognized mechanism by which FTO stabilizes the *HRAS* transcript and activates the PI3K/AKT pathway. While FTO has been shown to regulate lipid metabolism via m6A demethylase on transcripts such as *ChREBP*, *SREBF1*, *FASN*, *RUNX1T1*, and *PPARα* [15, 18, 47], our identification of *HRAS* as a target expands its functional repertoire in MASH. We further uncover a novel m6A modification site within exon 1 of *HRAS* (chr11: 535018–535730), distinct from previously reported 3′ UTR methylation [48], suggesting that FTO regulates *HRAS* primarily through mRNA stability rather than translational control. HRAS serves as a canonical upstream hub that activates the PI3K/AKT and RAF/MEK/ERK cascades [49, 50]. In MASH, our findings delineate a hyperactivated FTO/HRAS/PI3K/AKT axis, whereas the HRAS/RAF/MEK/ERK cascade is engaged to a lesser degree and warrants further investigation. Collectively, these findings offer mechanistic insights into how lipid stress‐induced *O*‐GlcNAcylation of FTO reprograms the m6A epitranscriptome to promote disease progression.

Based on this mechanism, we developed CPPs‐S195‐3#, a peptide designed to selectively disrupt *O*‐GlcNAcylation at FTO Ser195. This intervention increases m6A methylation on *HRAS* mRNA and consequently attenuates MASH progression. In contrast to the global *O*‐GlcNAcylation inhibitor OSMI‐1, which acts broadly on numerous OGT targets [9], CPPs‐S195‐3# selectively targets FTO Ser195 *O*‐GlcNAcylation, potentially minimizing off‐target effects. While the small‐molecule FTO inhibitor FB23‐2 has shown efficacy in AML [51], its therapeutic potential in MASH remains unexplored. Moreover, although resmetirom and semaglutide have been approved by the FDA for MASH treatment, future studies are warranted to explore whether combining them with CPPs‐S195‐3# exerts synergistic therapeutic benefits.

Despite these advances, several limitations should be considered. First, our mechanistic analyses focused primarily on hepatocytes, and it remains unclear whether non‐parenchymal cells, such as Kupffer cells, hepatic stellate cells, or endothelial cells, employ a similar *O*‐GlcNAcylation–FTO–m6A regulatory axis in MASH. Second, this CPP‐based targeting strategy, though effective in our experimental settings, requires further optimization for liver‐specific delivery to maximize therapeutic efficacy and safety. Finally, although *HRAS* was identified as a key downstream target of FTO, additional m6A‐modified transcripts likely contribute to MASH progression, warranting more comprehensive methylomic and proteomic analyses to fully elucidate FTO‐dependent epitranscriptomic rewiring.

In conclusion, we demonstrate that lipid stress activates the HRAS/PI3K/AKT pathway via *O*‐GlcNAcylation of FTO at Ser195, promoting MASH pathogenesis. These findings establish a critical link between nutrient‐sensitive signaling and epitranscriptomic regulation, highlighting the therapeutic potential of targeting the *O*‐GlcNAc‐FTO axis.

## 4 Experimental Section

### 4.1 RNA Extraction and Real‐Time PCR

Total RNA was isolated from liver tissues or cells using TRIzol reagent (15596018, Invitrogen) according to the manufacturer's instructions. cDNA was reverse‐transcribed from RNA using the PrimeScript RT Reagent Kit (RR047A, TaKaRa, Japan). Quantitative real‐time PCR was performed using a CFX Connect Real‐Time PCR Detection System (Bio‐Rad) with specific primers (Table S1). Relative mRNA expression levels were normalized to β‐actin and calculated using the 2^(−△△C(T))^ method.

### 4.2 Western Blotting and Antibodies

Liver tissues or cell samples were homogenized and lysed in cold lysis buffer (P10013, Beyotime) supplemented with 1x protease inhibitor (C0001, TargetMol) and 1x phosphatase inhibitor (C0002, TargetMol). The lysates were vortexed, sonicated, and centrifuged at 14000 g for 20 min at 4°C. The supernatant protein was quantified using the BCA Protein Assay Kit (P0398M, Beyotime). Equal amounts of protein were separated by SDS‐PAGE and transferred to PVDF membranes (IPVH00010, Millipore, MA, USA). Membranes were blocked with 5% non‐fat milk and incubated with the antibodies (Table S2) overnight at 4°C.

### 4.3 Patient Specimens/Subjects

Clinical samples consisted of serial paraffin‐embedded liver sections from histologically confirmed MASH patients (n = 15) and healthy donors (n = 6), obtained from the First Affiliated Hospital of Wenzhou Medical University and the Second Affiliated Hospital of Chongqing Medical University.

### 4.4 Study Approval

All animal experiments were conducted in accordance with the Guide for the Care and Use of Laboratory Animals and were approved by the Institutional Animal Care and Use Committee of Chongqing Medical University (IACUC‐CQMU‐2025‐0041).

The serial liver sections of healthy or MASH patients were from the First Affiliated Hospital of Wenzhou Medical University and the Second Hospital of Chongqing Medical University with written informed consent and approved by the Institutional Ethical Review Board of Wenzhou Medical University (approval no. 2018–059) and Chongqing Medical University (approval no. 309/2025), respectively.

### 4.5 Statistical Analysis

Statistical analysis was conducted using the GraphPad Prism software (version 8.0, La Jolla, CA, USA). Data are presented as mean ± standard deviation (SD). Experiments were performed with three biological replicates (n = 3), each measured in technical triplicates. Animal studies used five mice (n = 5) per group. Differences between two groups were determined using the Student's t‐test. Differences between multiple groups were determined by one‐way ANOVA. ^*^ *p* < 0.05, ^**^ *p* < 0.01, ^***^ *p* < 0.001 and ns, not significant.

The detailed methodology and experimental procedures are provided in the online supplemental materials.

## Author Contributions

J.Y., Y. Z., and Q.L. performed major experiments and data analysis. J.Y. and R.L. performed metabolic, proteomic, and MeRIP‐seq data analysis. J.Y., Y. Z., and Q.L. drafted the manuscript. N.T., K.W., and A.H. revised the manuscript with all authors providing feedback. M.Z., Y.Y., and D.C. collected clinical data. N.T., K.W., R.L., and A.H. provided data curation, funding acquisition, project administration, and supervision

## Funding

This work was supported by the National Natural Science Foundation of China (82272975 (NT) and 82573249 (KW) used for data collection, analysis, and interpretation), the Natural Science Foundation Project of Chongqing (CSTB2025NSCQ‐LZX0145, NT; Used for data collection and analysis), Senior Medical Talents Program of Chongqing for Young and Middle‐aged, the Kuanren talents and Joint project of Pinnacle Disciplinary Group, the Second Affiliated Hospital of Chongqing Medical University, and the Chongqing Professional Talents Plan for Innovation and Entrepreneurship Demonstration Team (CQYC202203091047, NT; Used for data collection and analysis), Chongqing Natural Science Foundation General Program (CSTB2025NSCQ‐GPX1193, RL; Used for data analysis).

## Conflicts of Interest

The authors declare no conflicts of interest.

## Supporting information




**Supporting File 1**: advs78218‐sup‐0001‐SuppMat.docx.

**Supporting File 2**: advs78218‐sup‐0002‐SuppMat.pdf.

## Data Availability

The original mass spectrometry analysis data of metabolic profile and *O*‐GlcNAcylomics were deposited on Big Sub (OMIX012913 and OMIX012916, respectively). The original RNA‐ seq and MeRIP‐seq data were deposited in the Genome Sequence Archive (GSA) database (HRA014606).
