# Supplementary material for: Hepatic Lipid Stress Drives Metabolic Dysfunction‐Associated Steatohepatitis Progression through FTO O‐GlcNAcylation‐Induced m6A Demethylation of HRAS
Source: Adv Sci (Weinh). 2026 Oct 11:e78218. Online ahead of print. doi: 10.1002/advs.78218 (PMC13655264; doi:10.1002/advs.78218)
Supplement: Supplementary file 1 [file ADVS-9999-e78218-s002.pdf]

Figure 1F

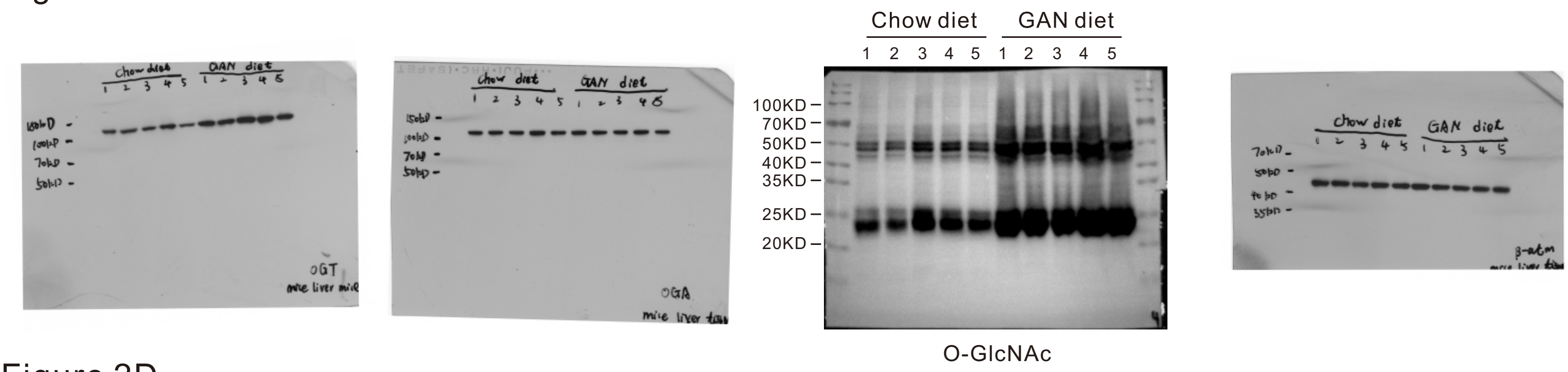

Figure 2D

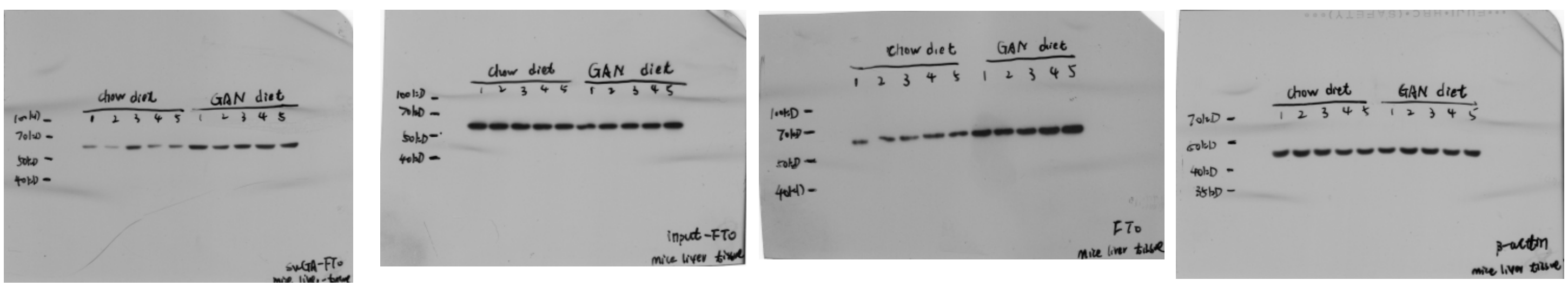

Figure 2E

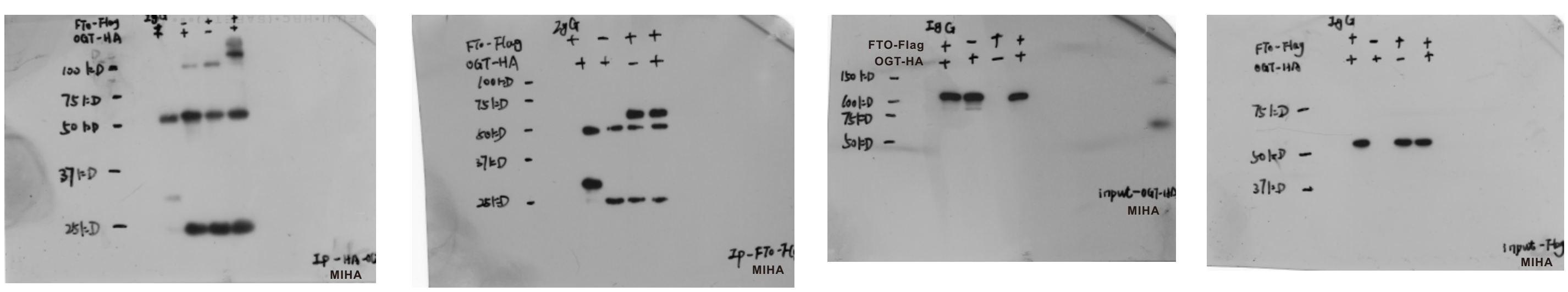

Figure 2F

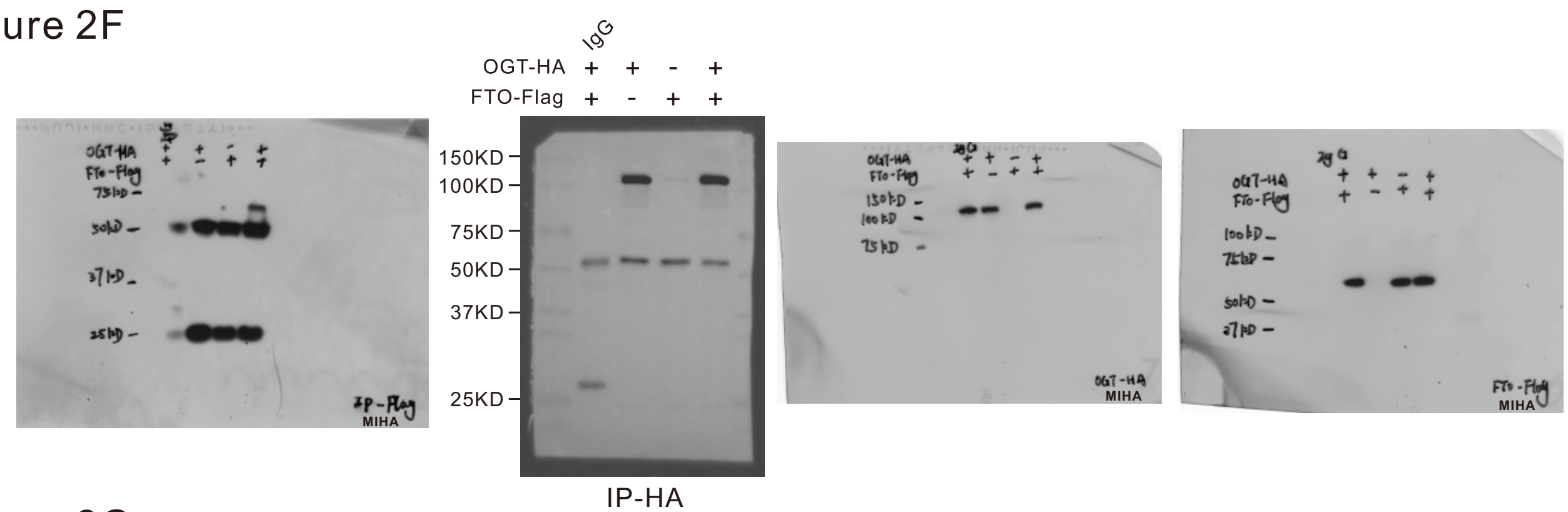

Figure 2G

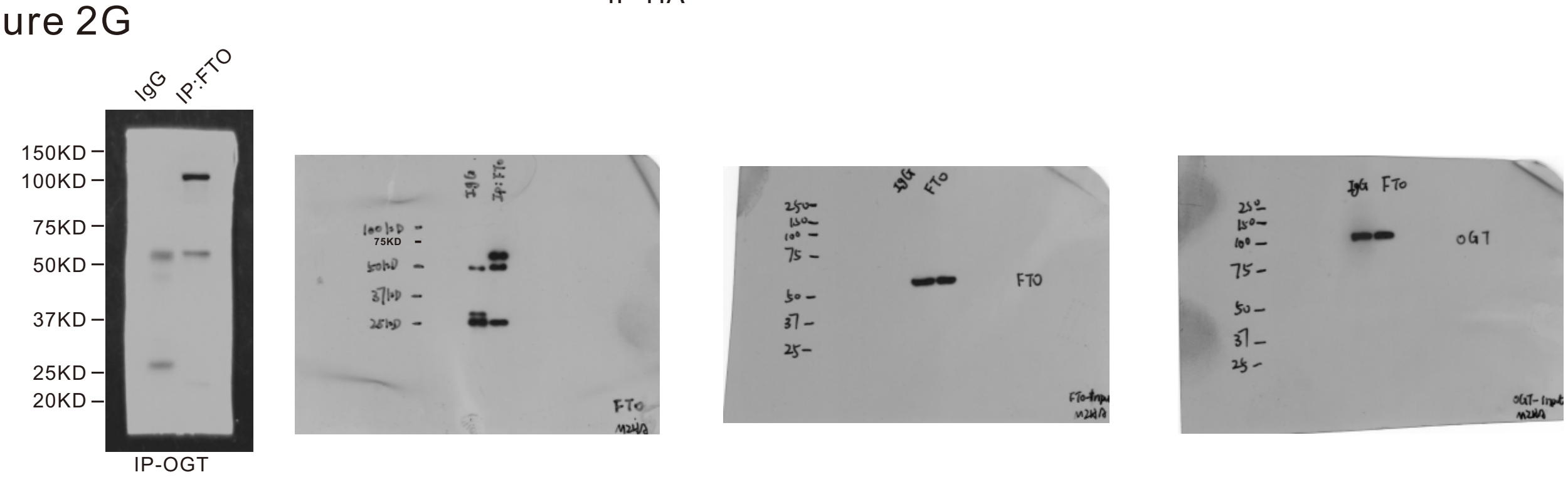

Figure 2J

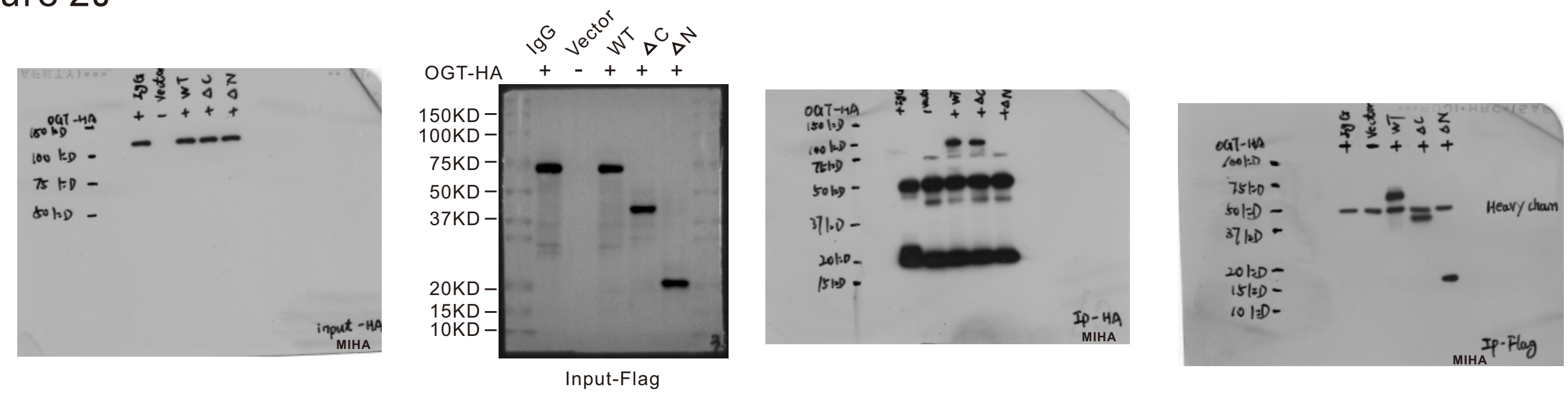

Figure 2M

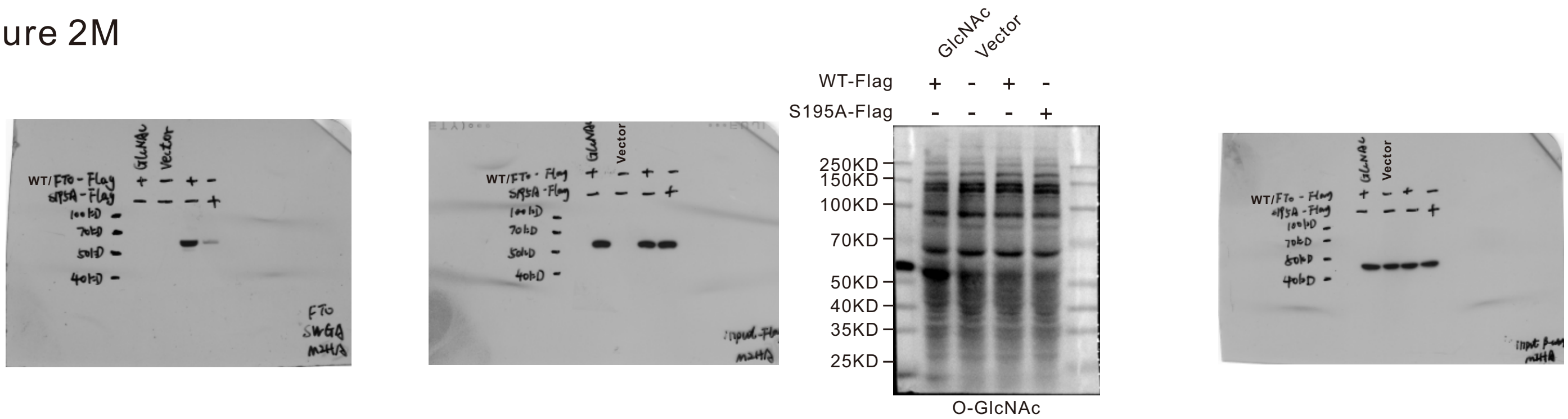

Figure 2N

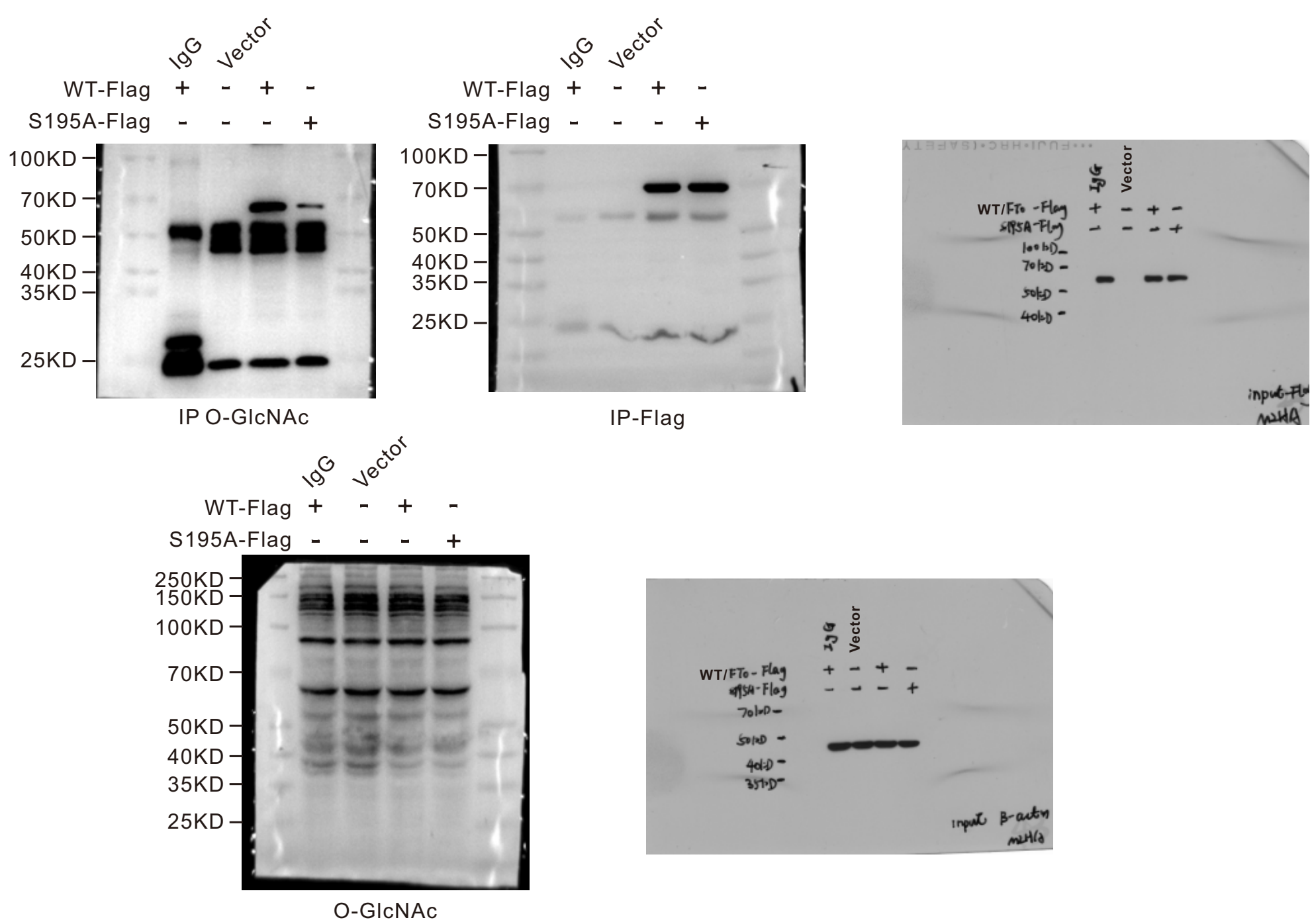

Figure 3A

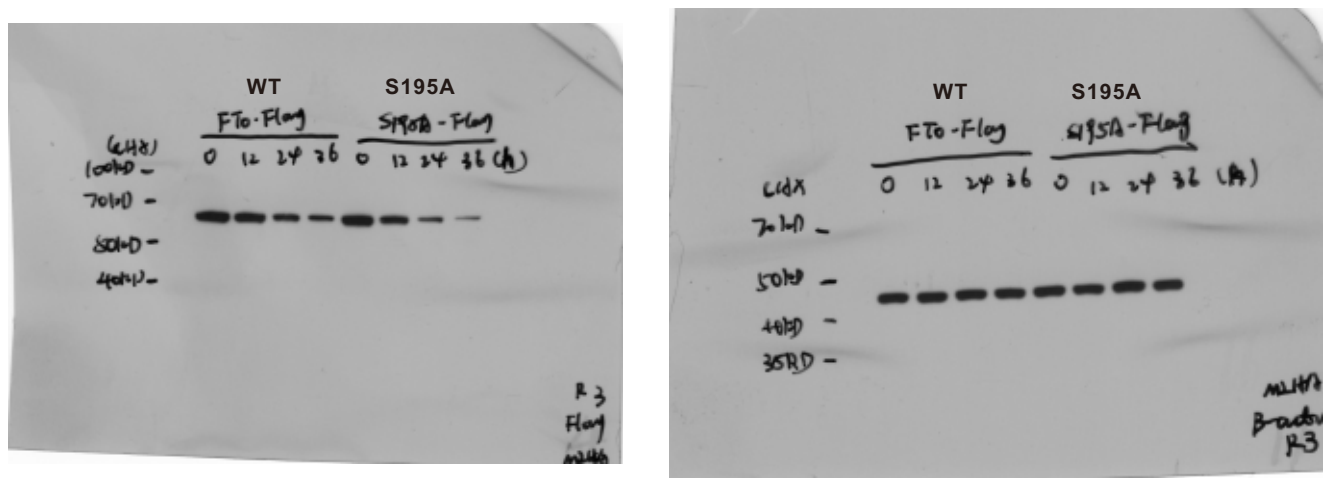

Figure 3B

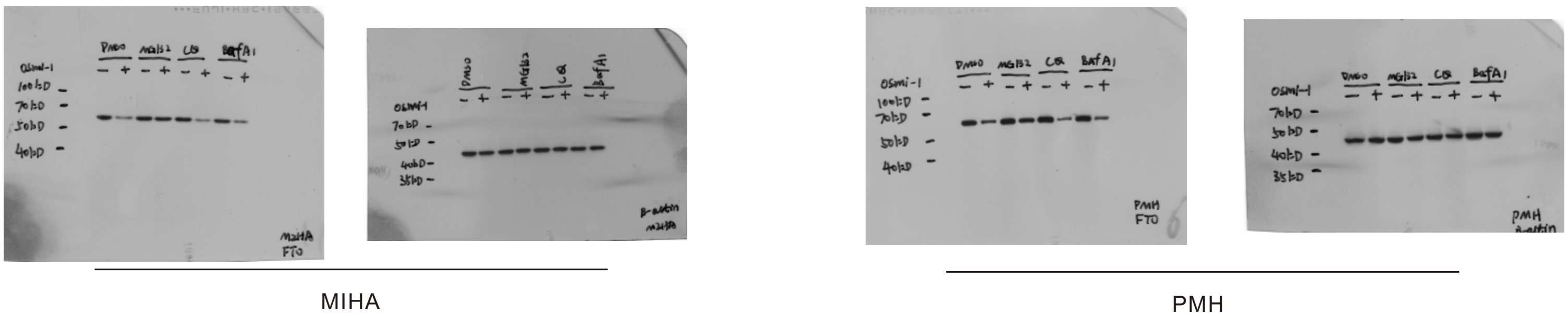

Figure 3C

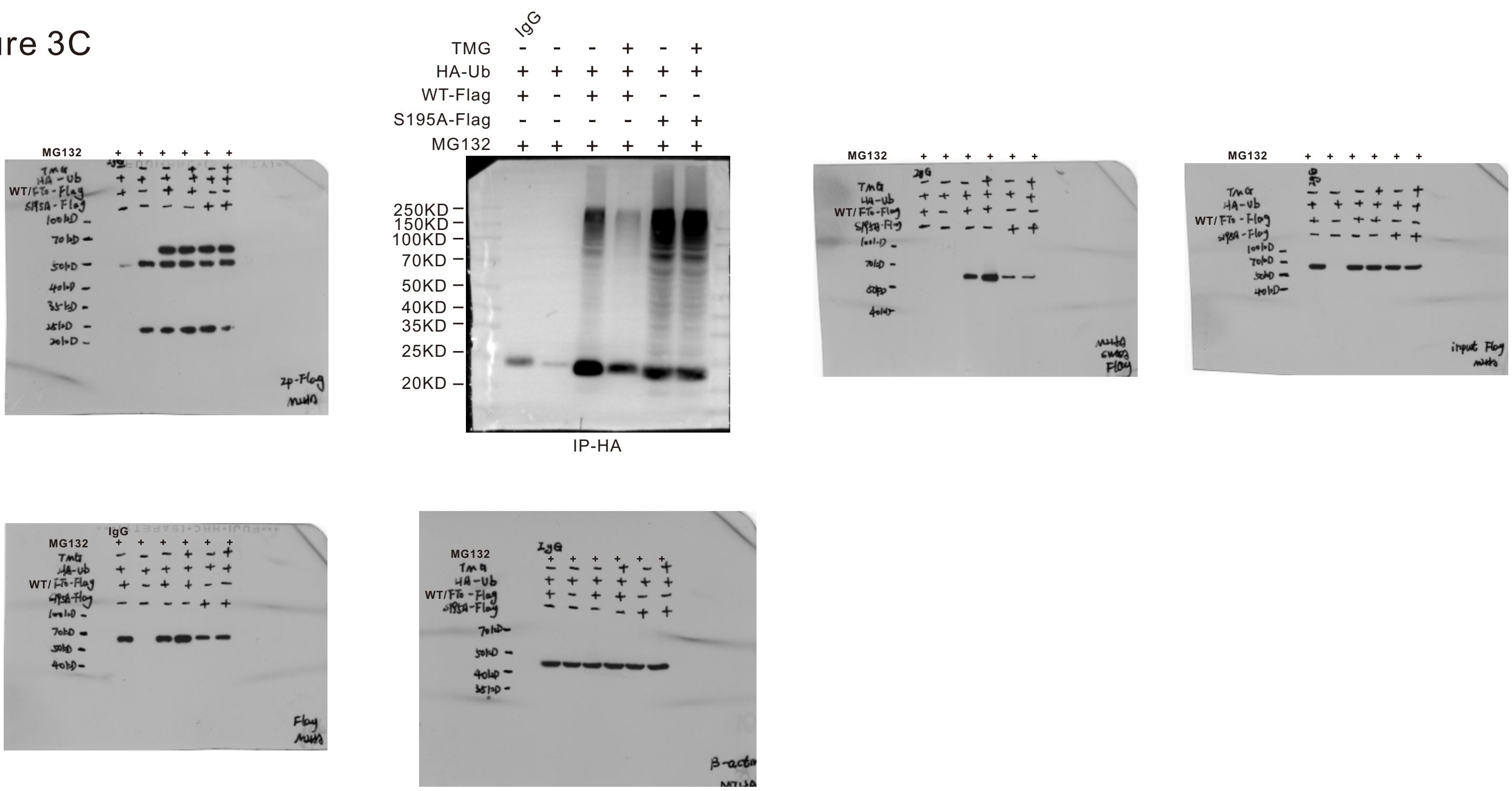

Figure 3D

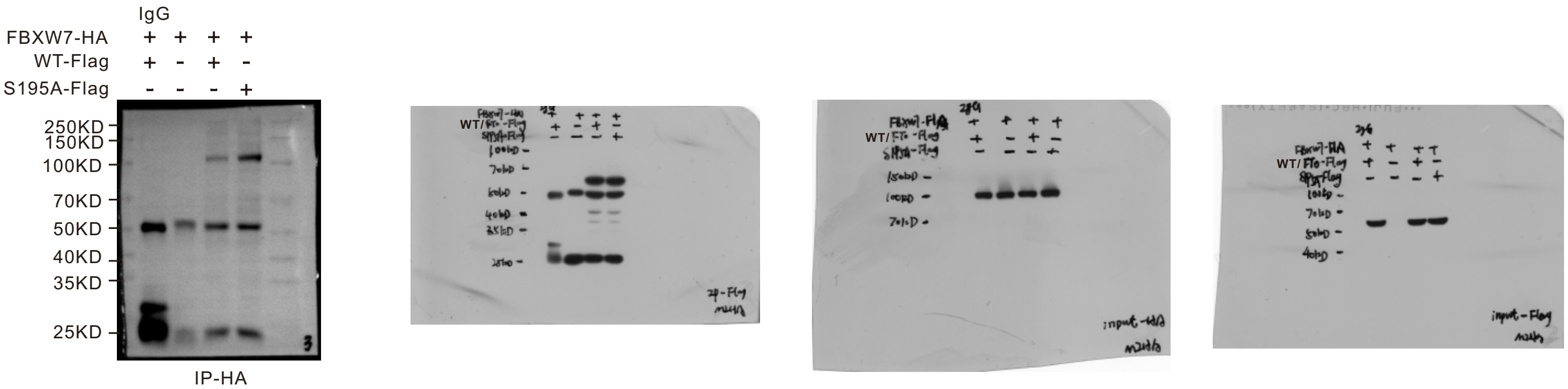

Figure 3E

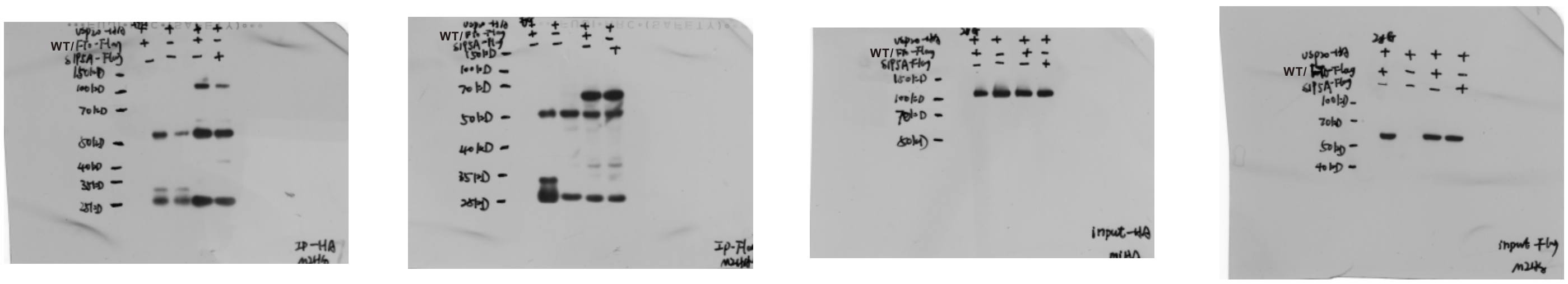

Figure 3F

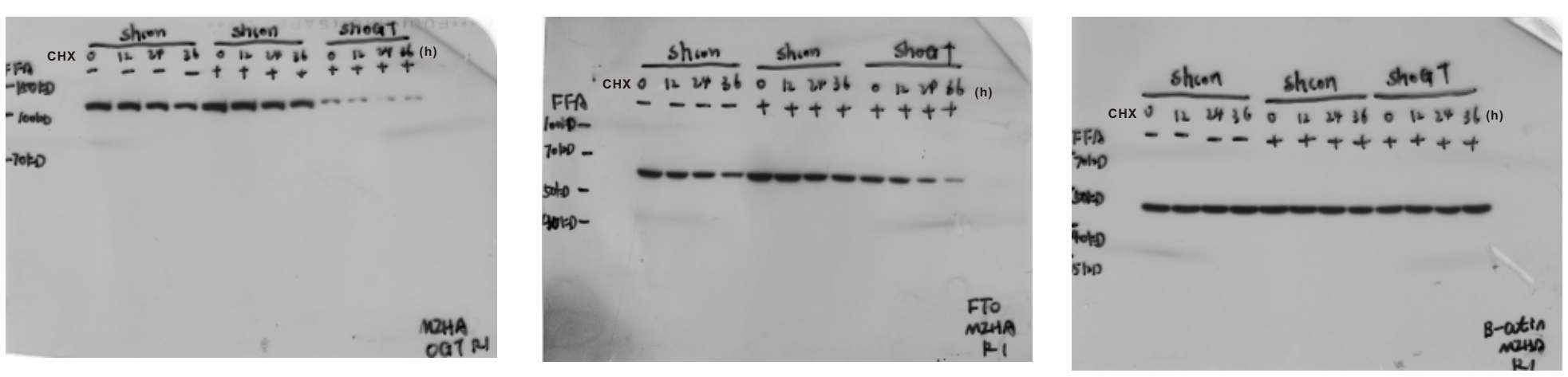

Figure 3G

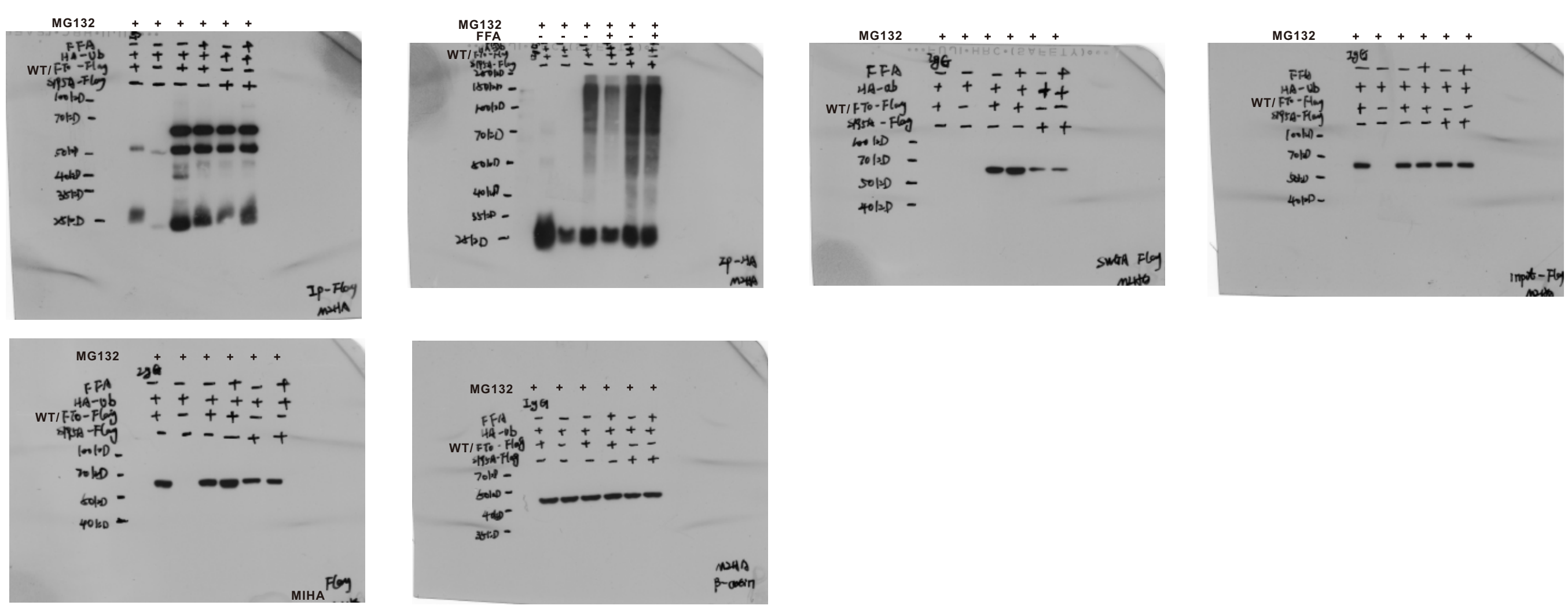

Figure 3H

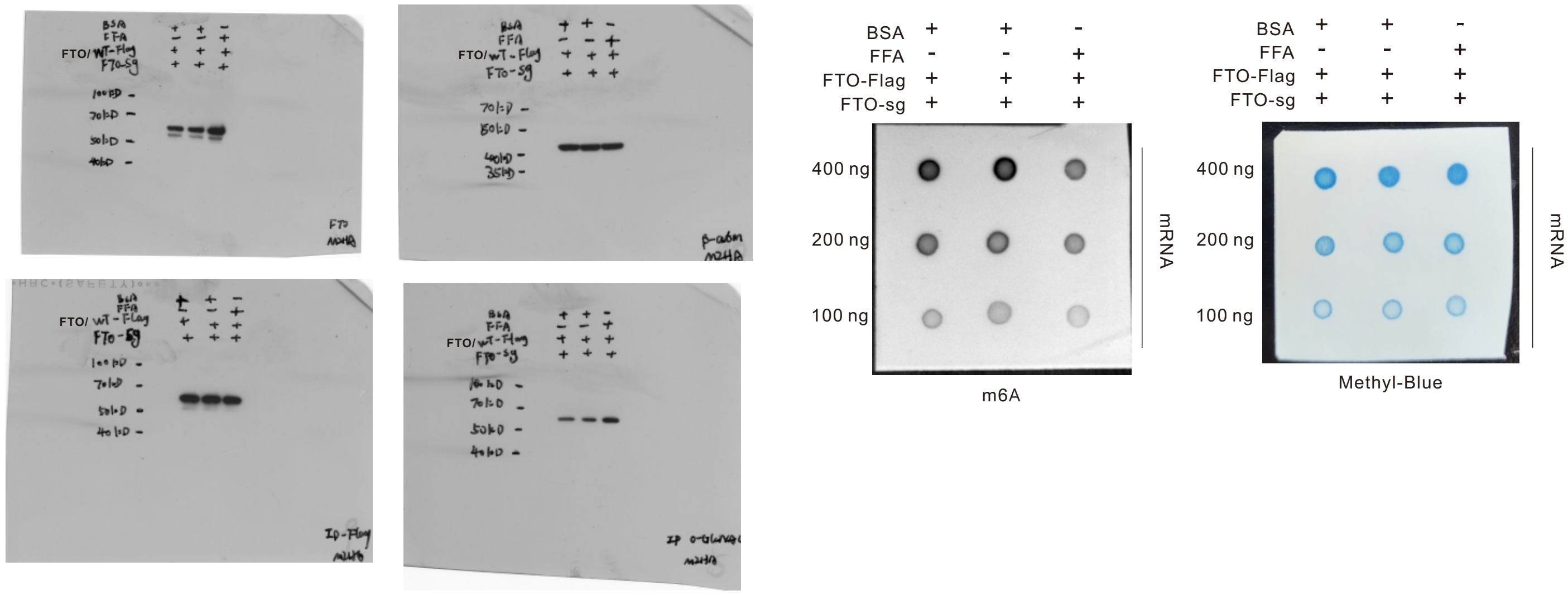

Figure 3I

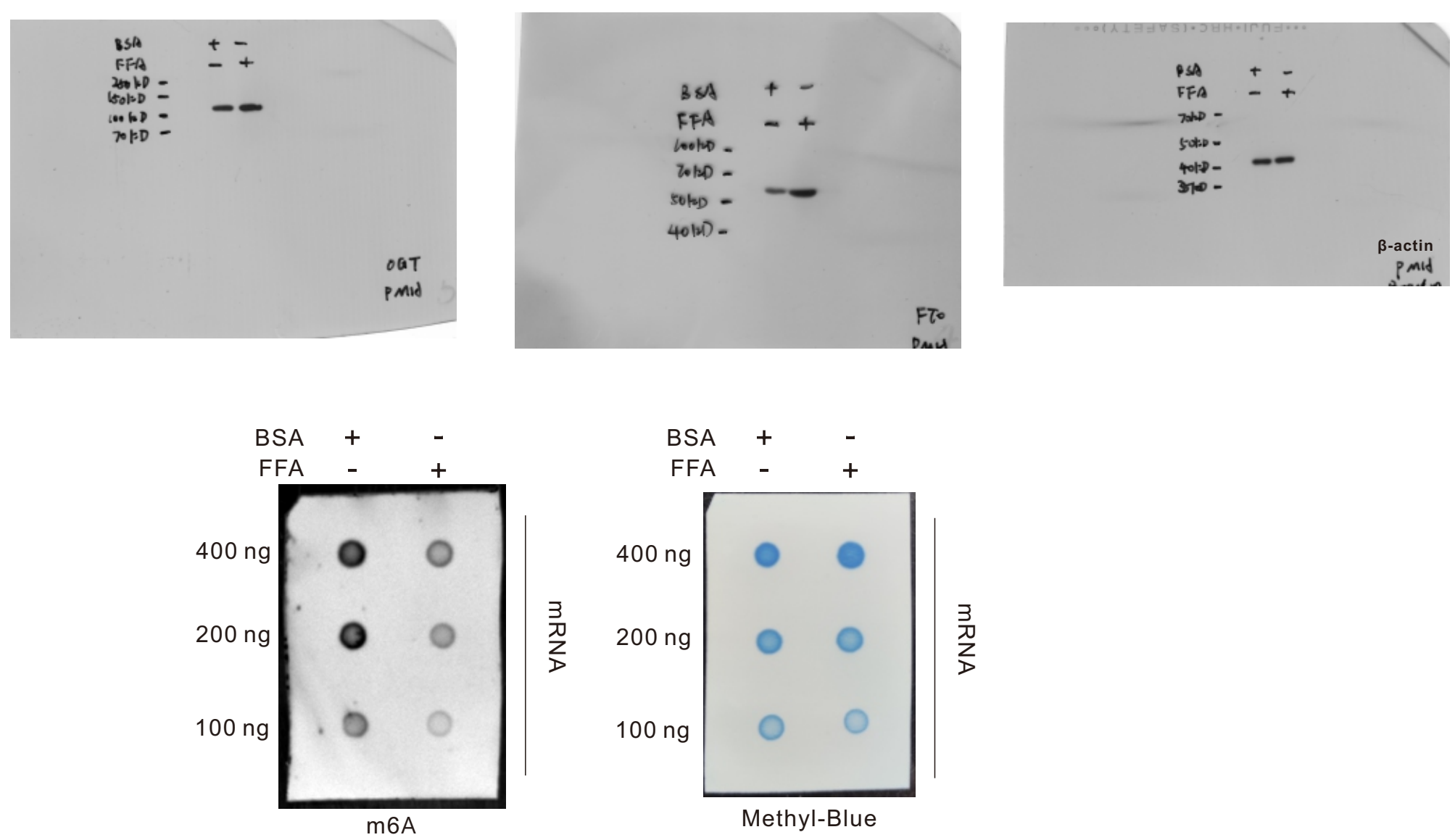

Figure 3J

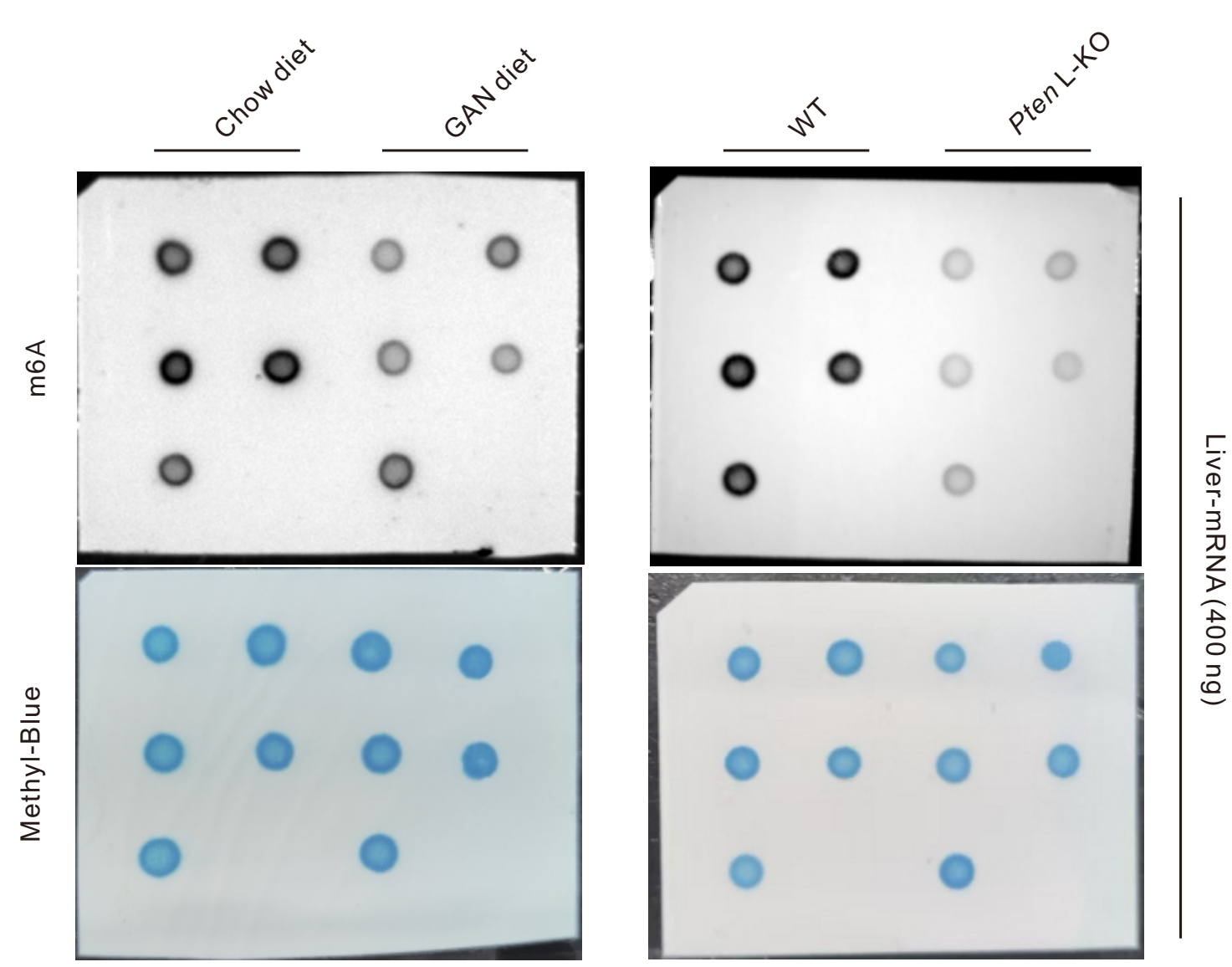

Figure 4B

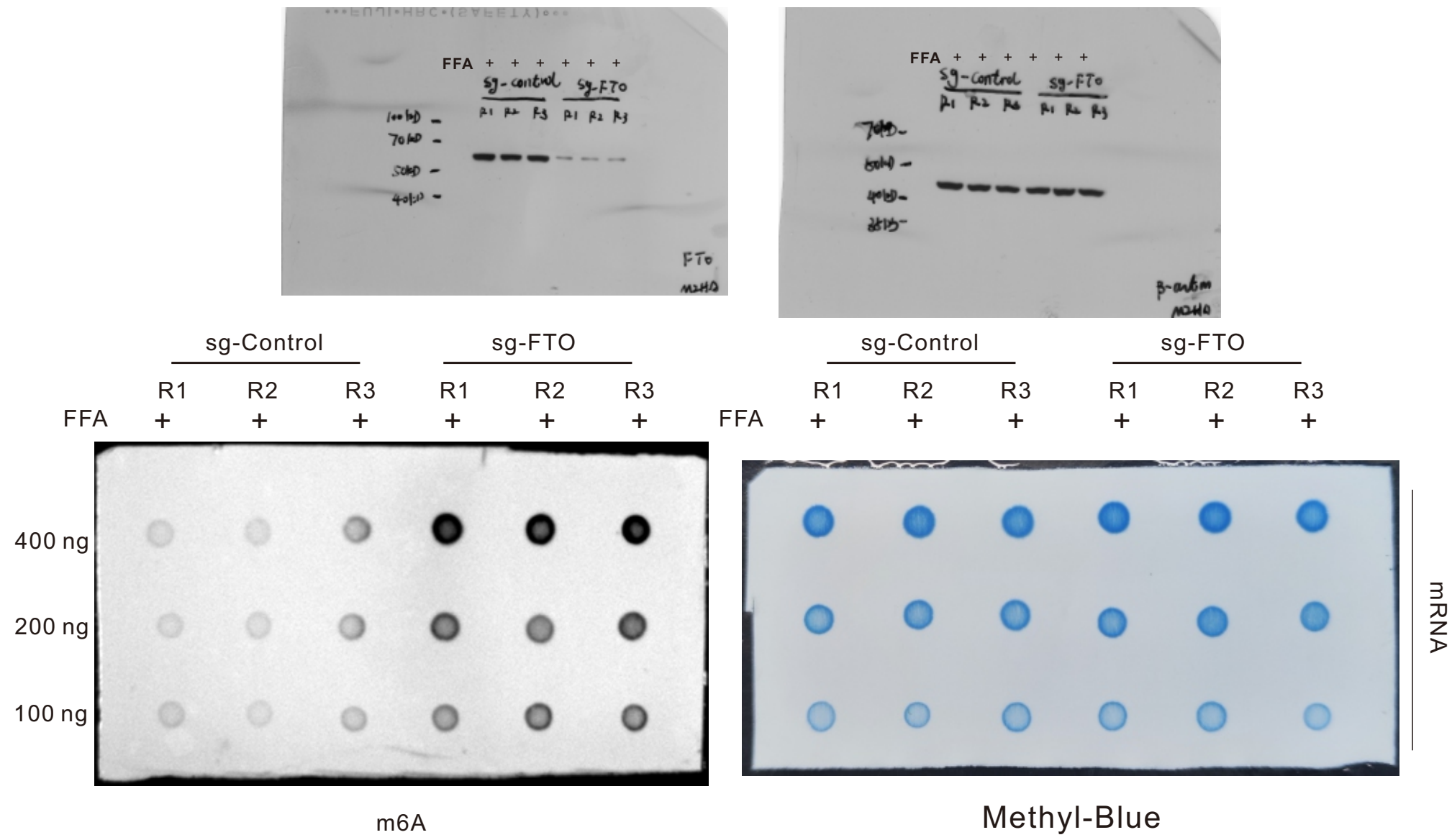

Figure 4K

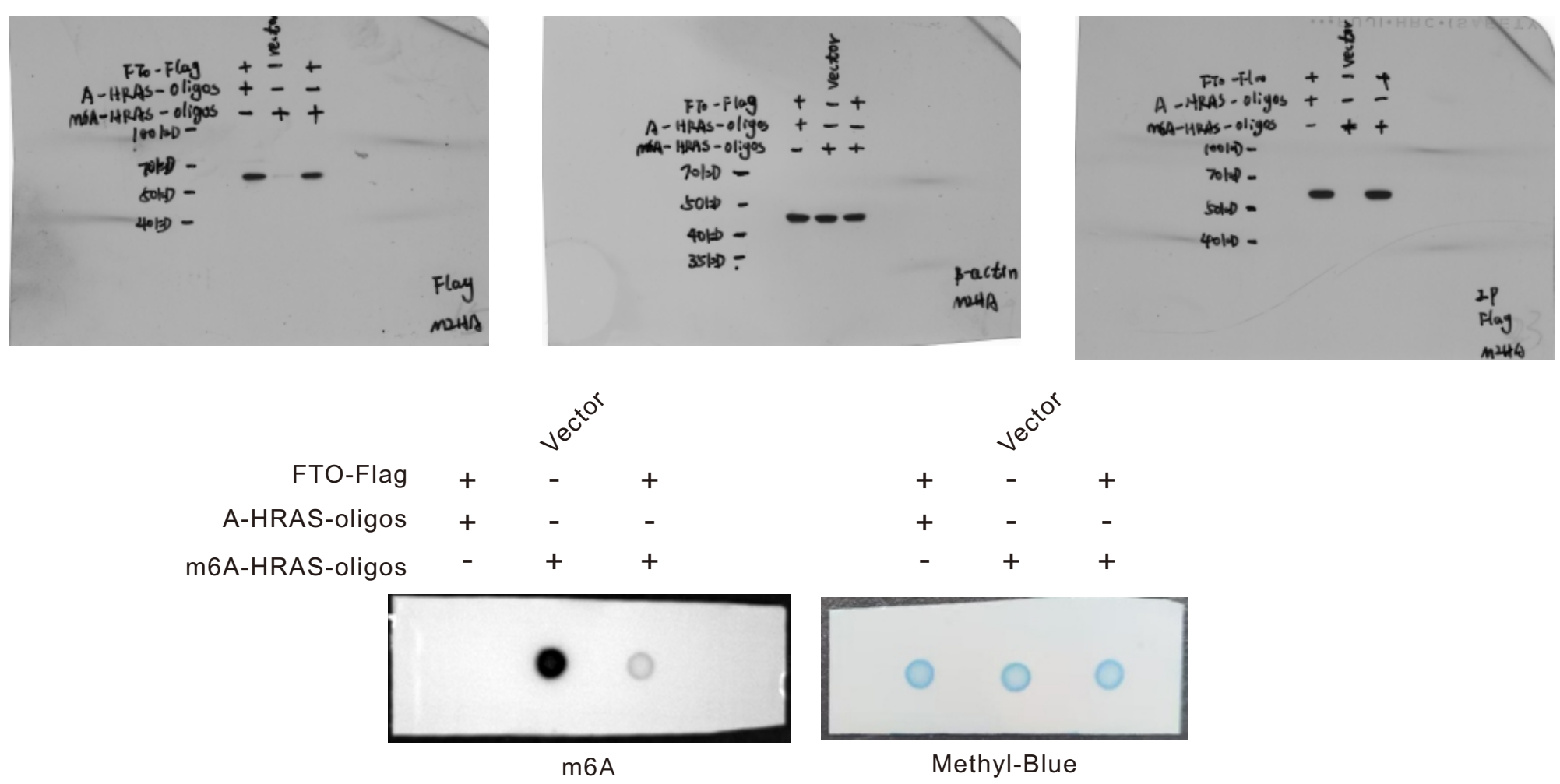

Figure 4L

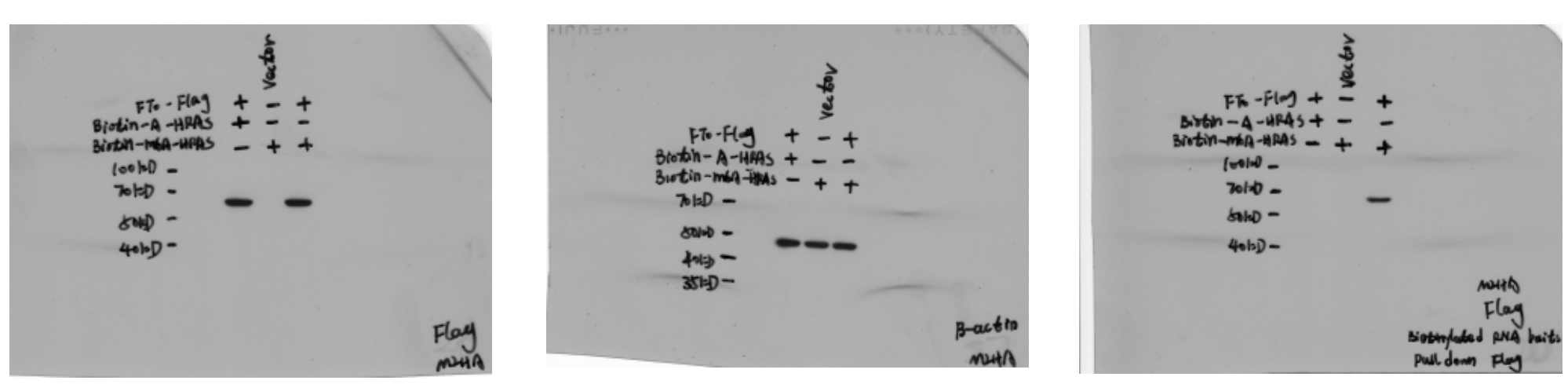

Figure 5C

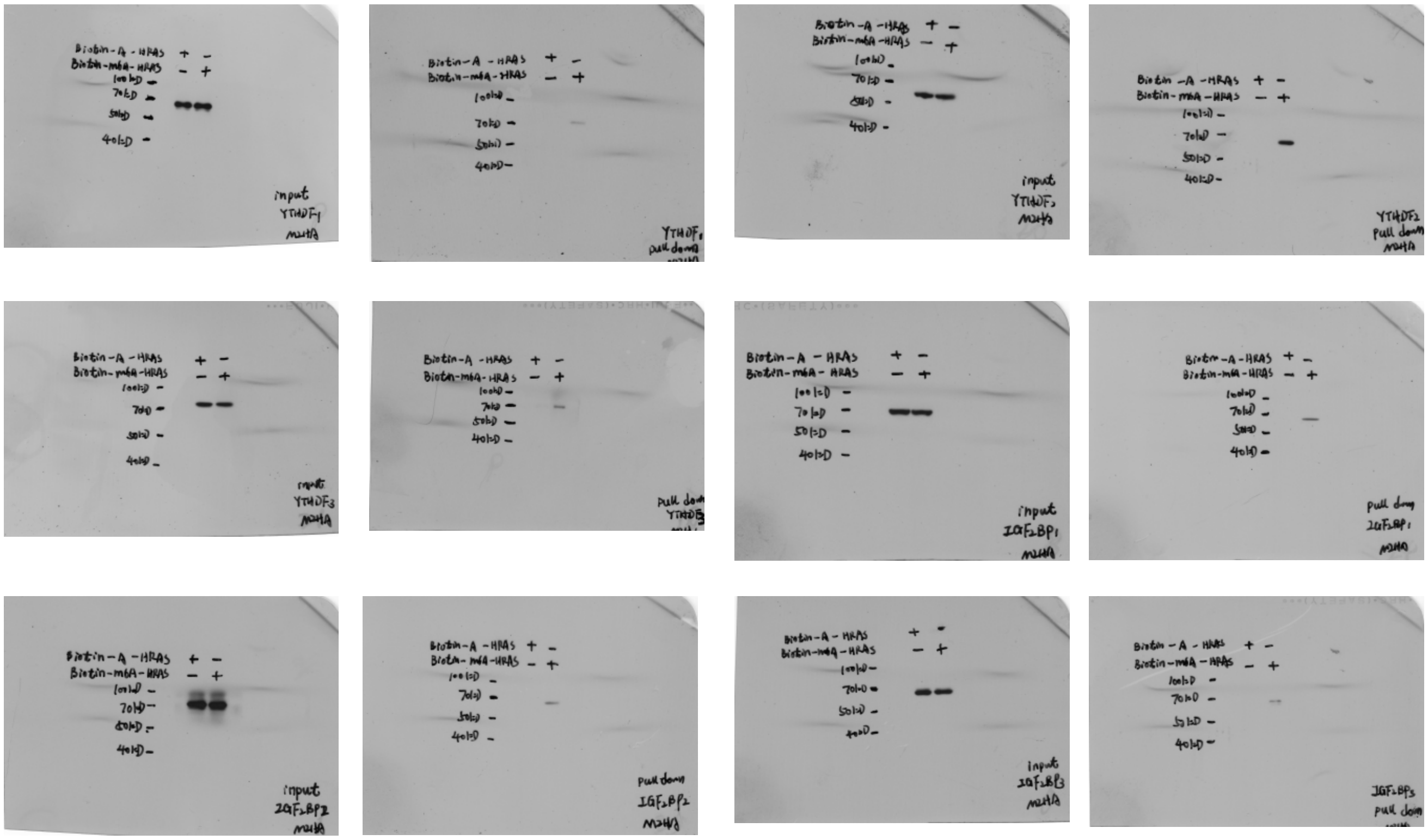

Figure 5F

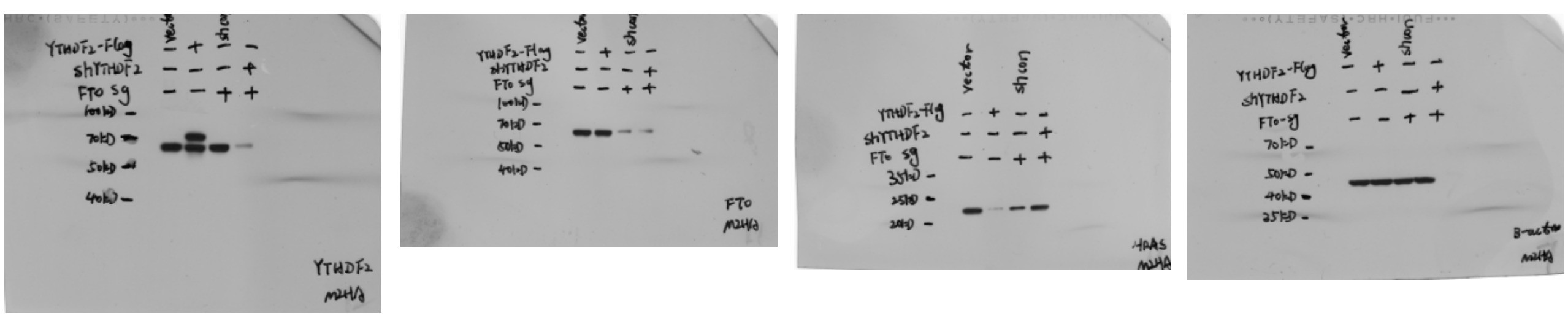

Figure 5G

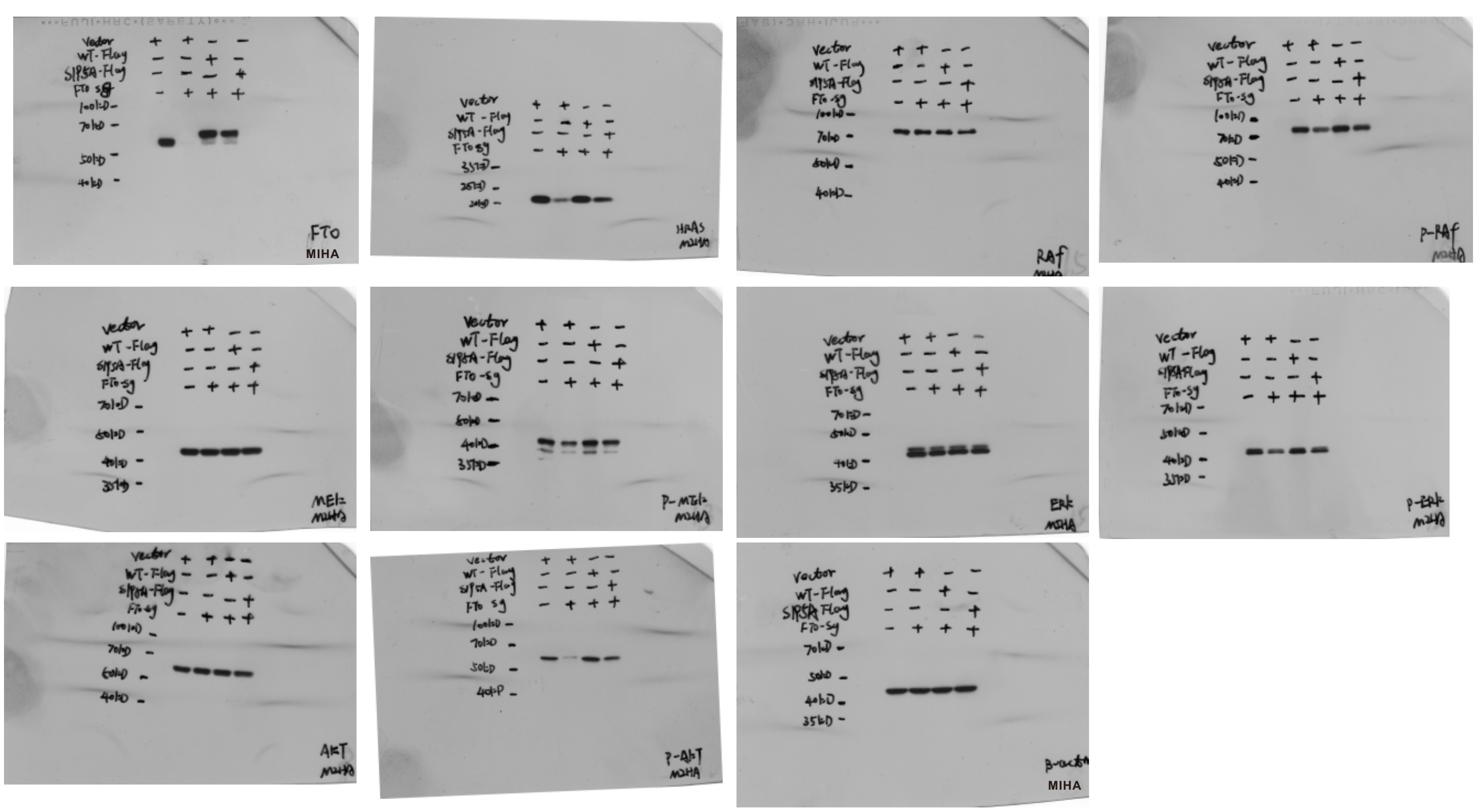

Figure 5H

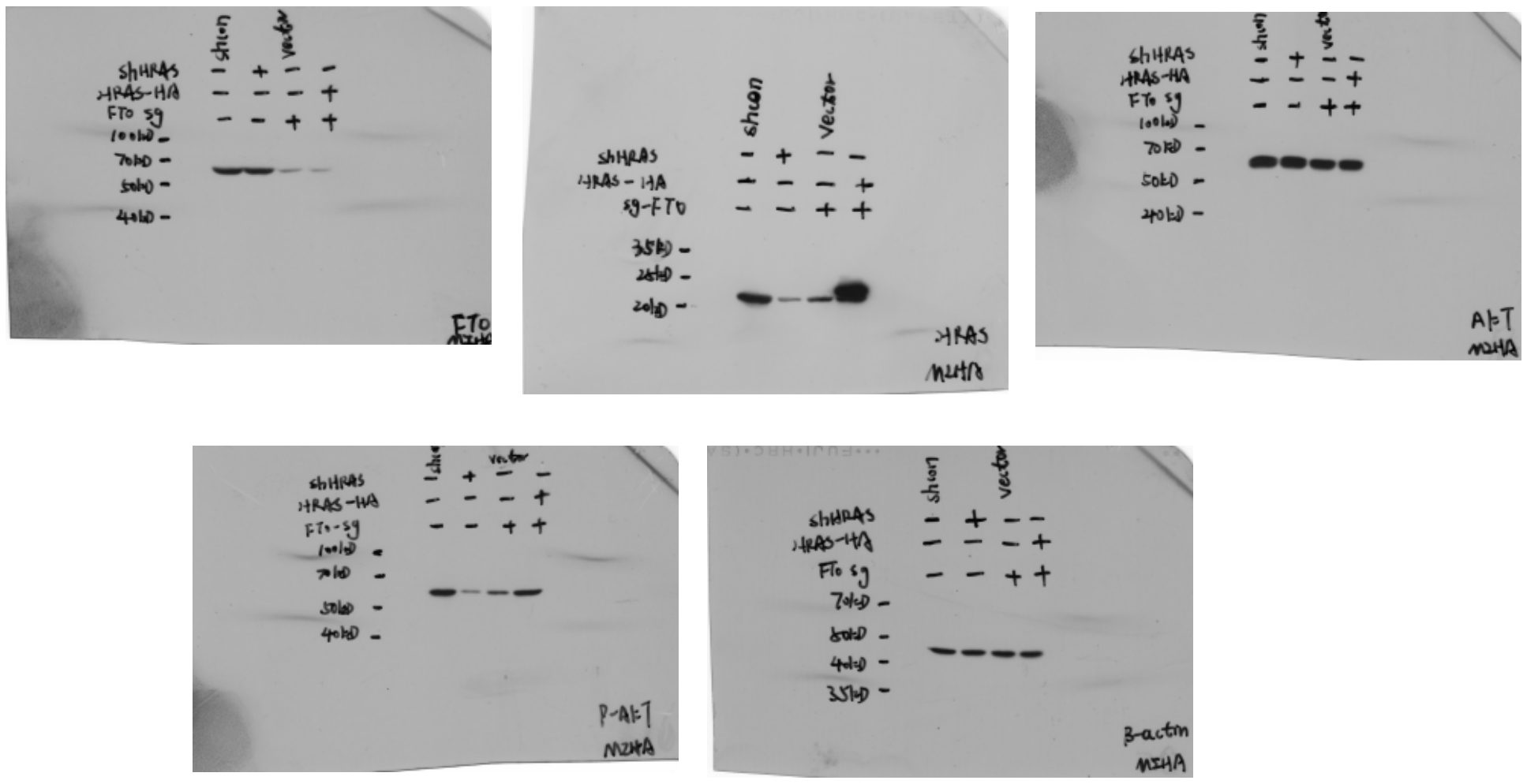

Figure 5I

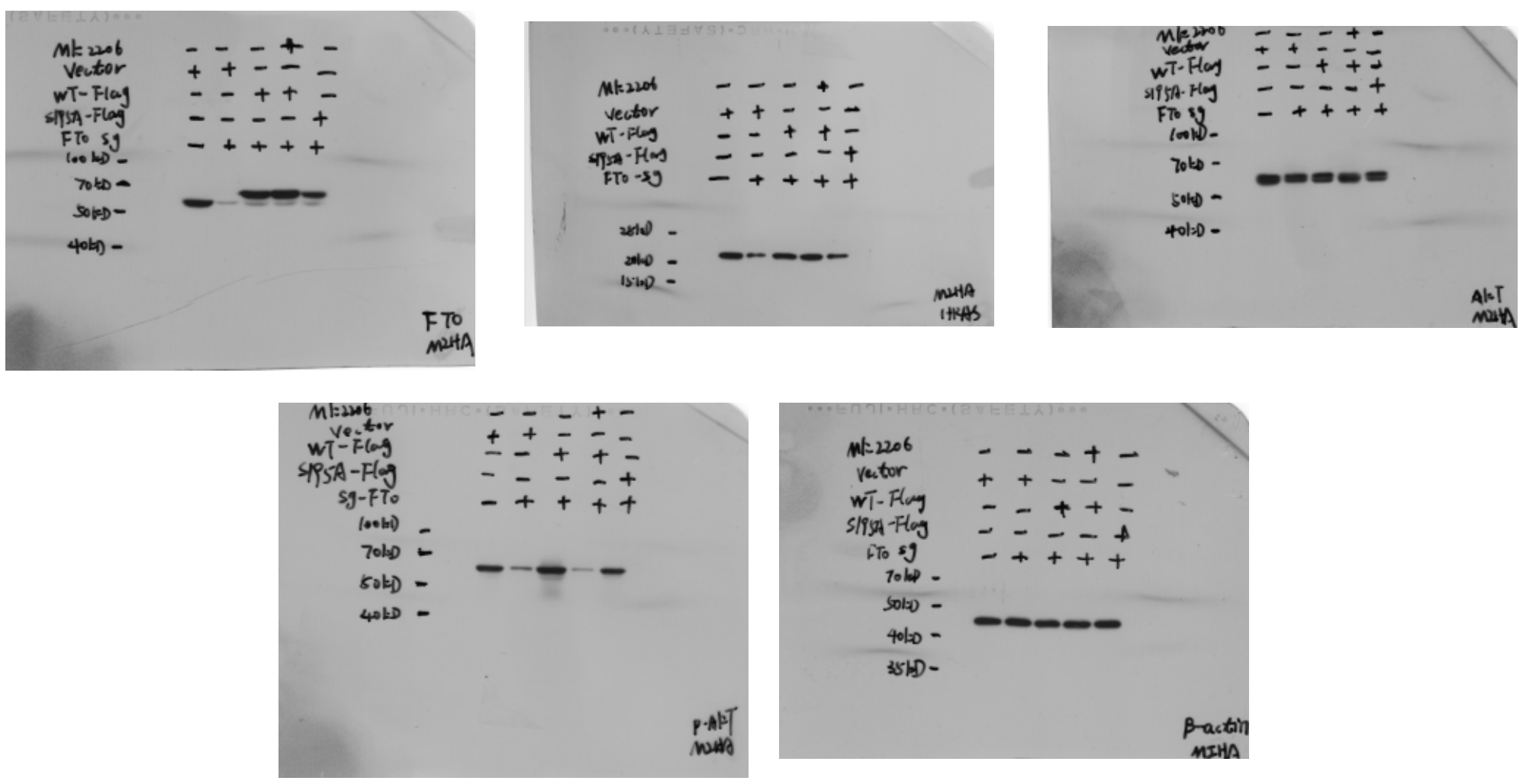

Figure 7B

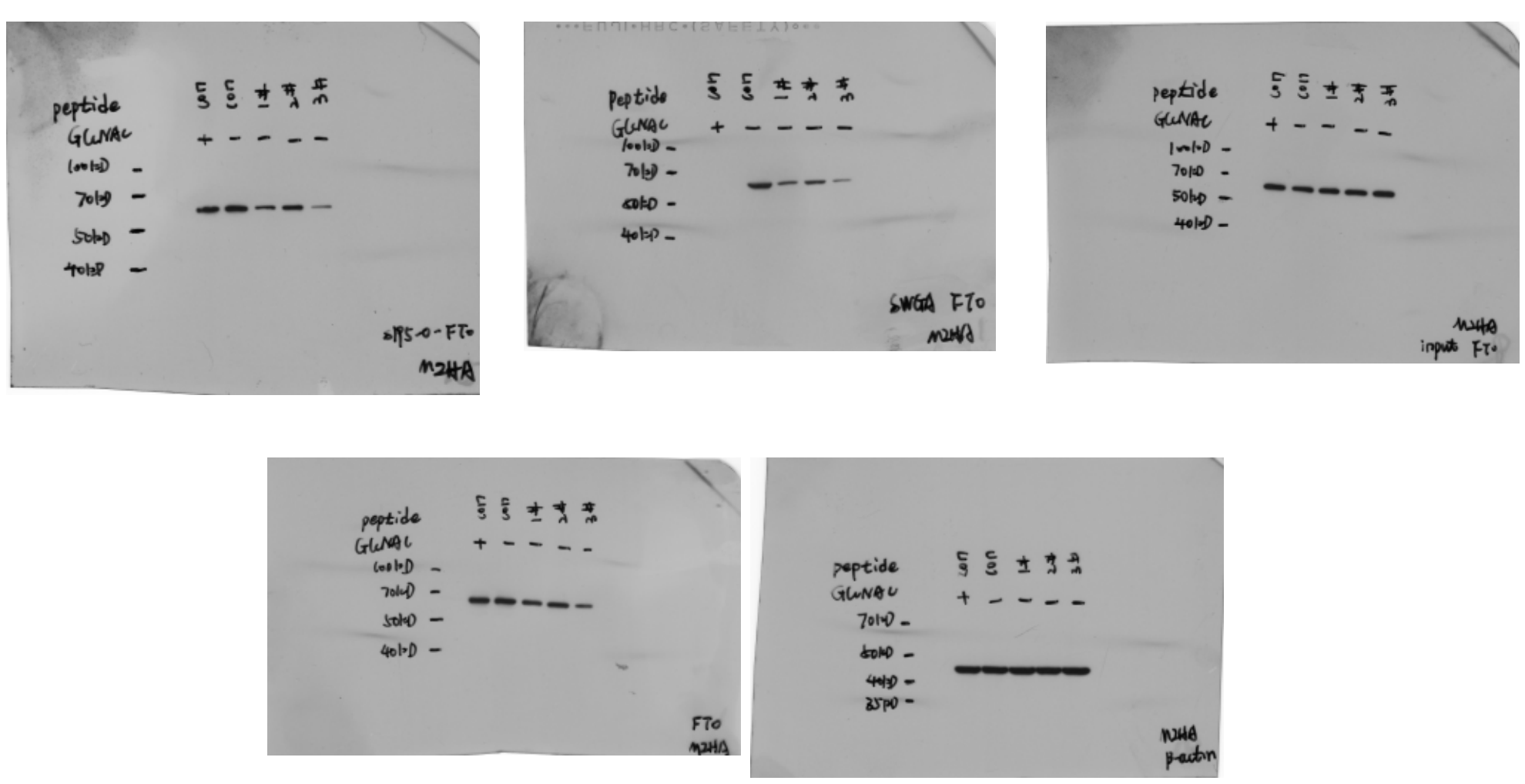

Figure 7C

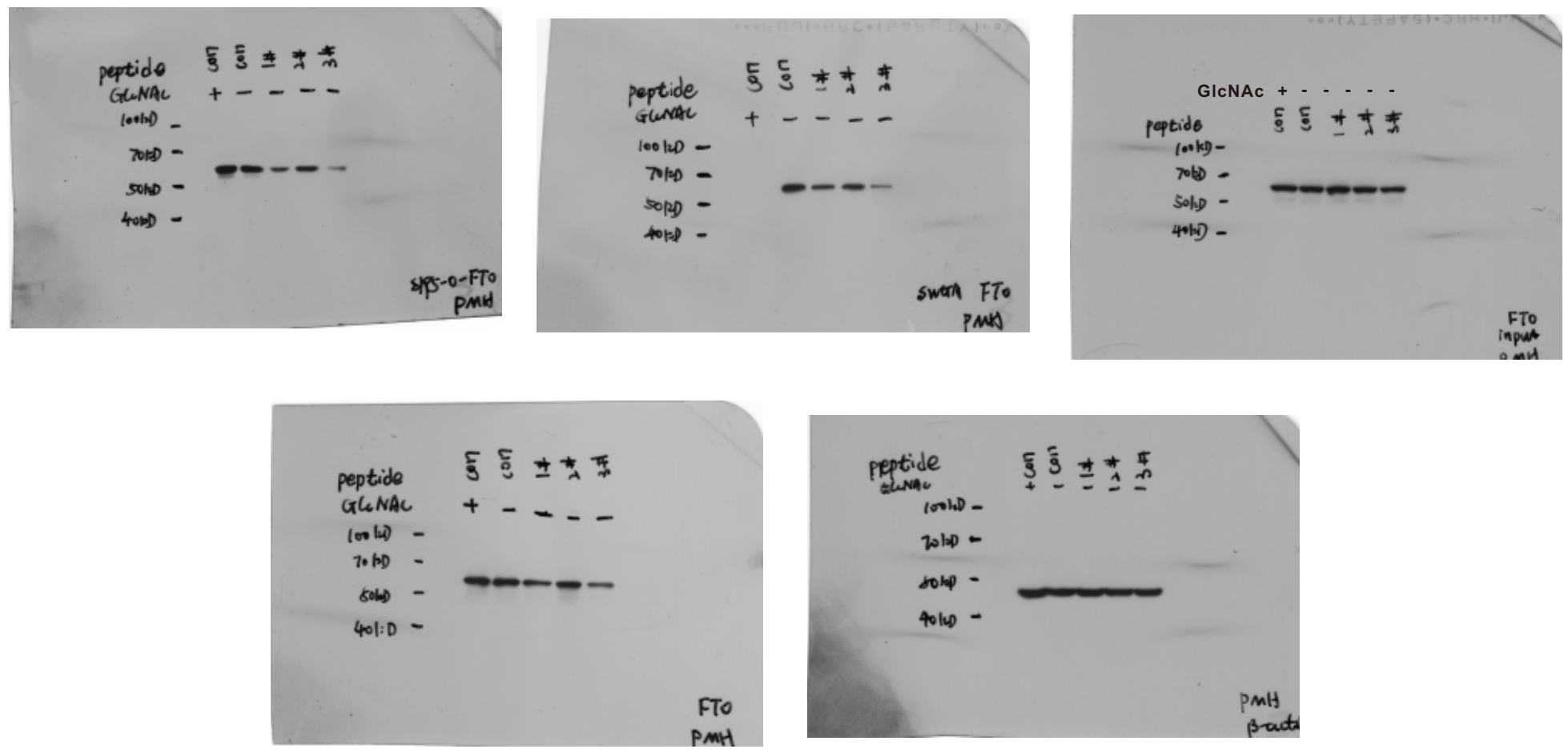

Figure 7D

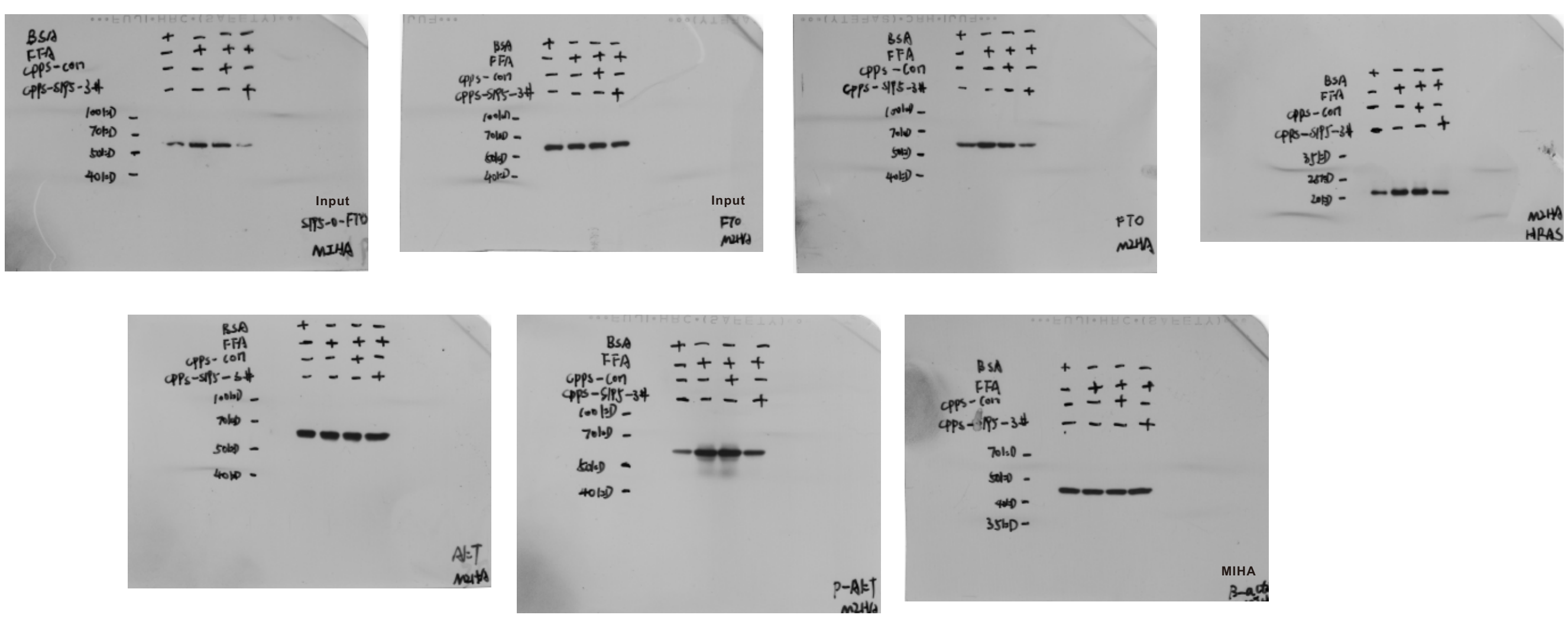

Figure S3E

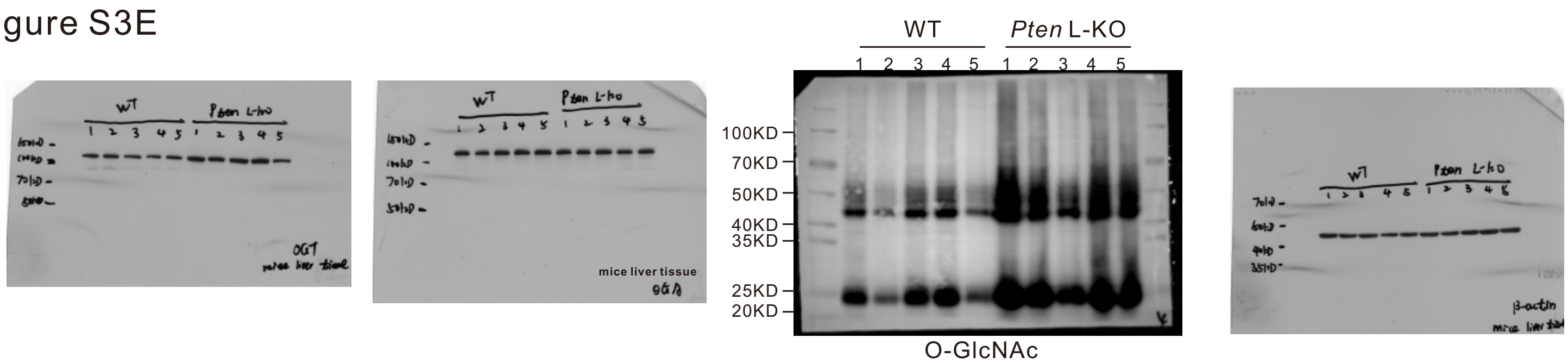

Figure S4D

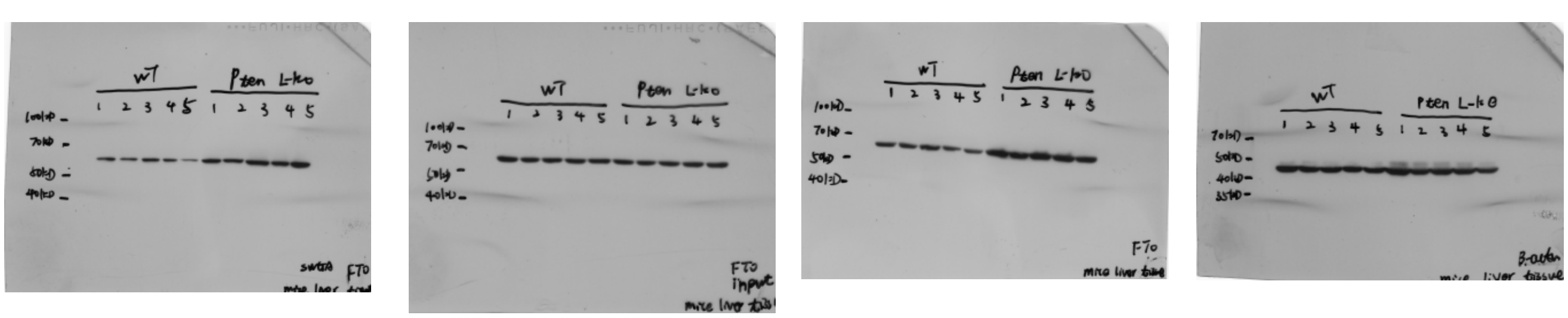

Figure S5A

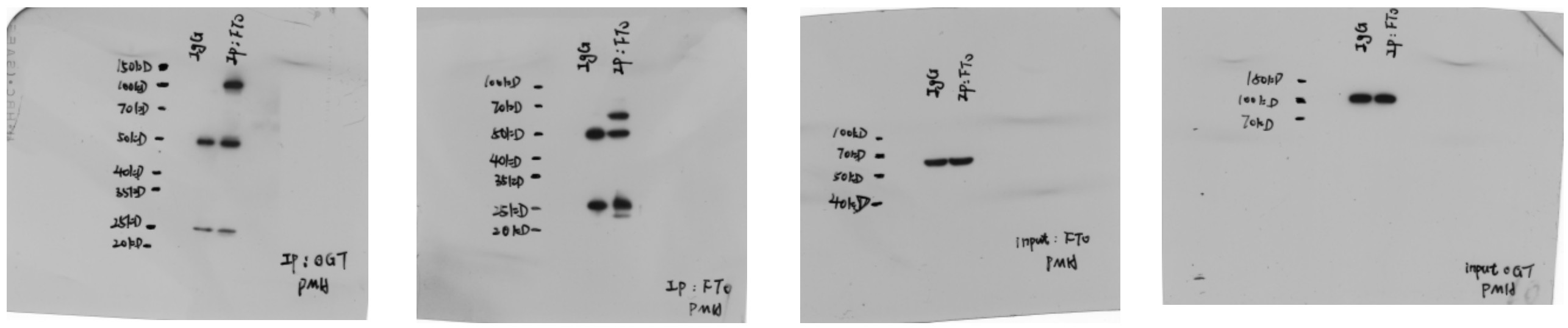

Figure S5C

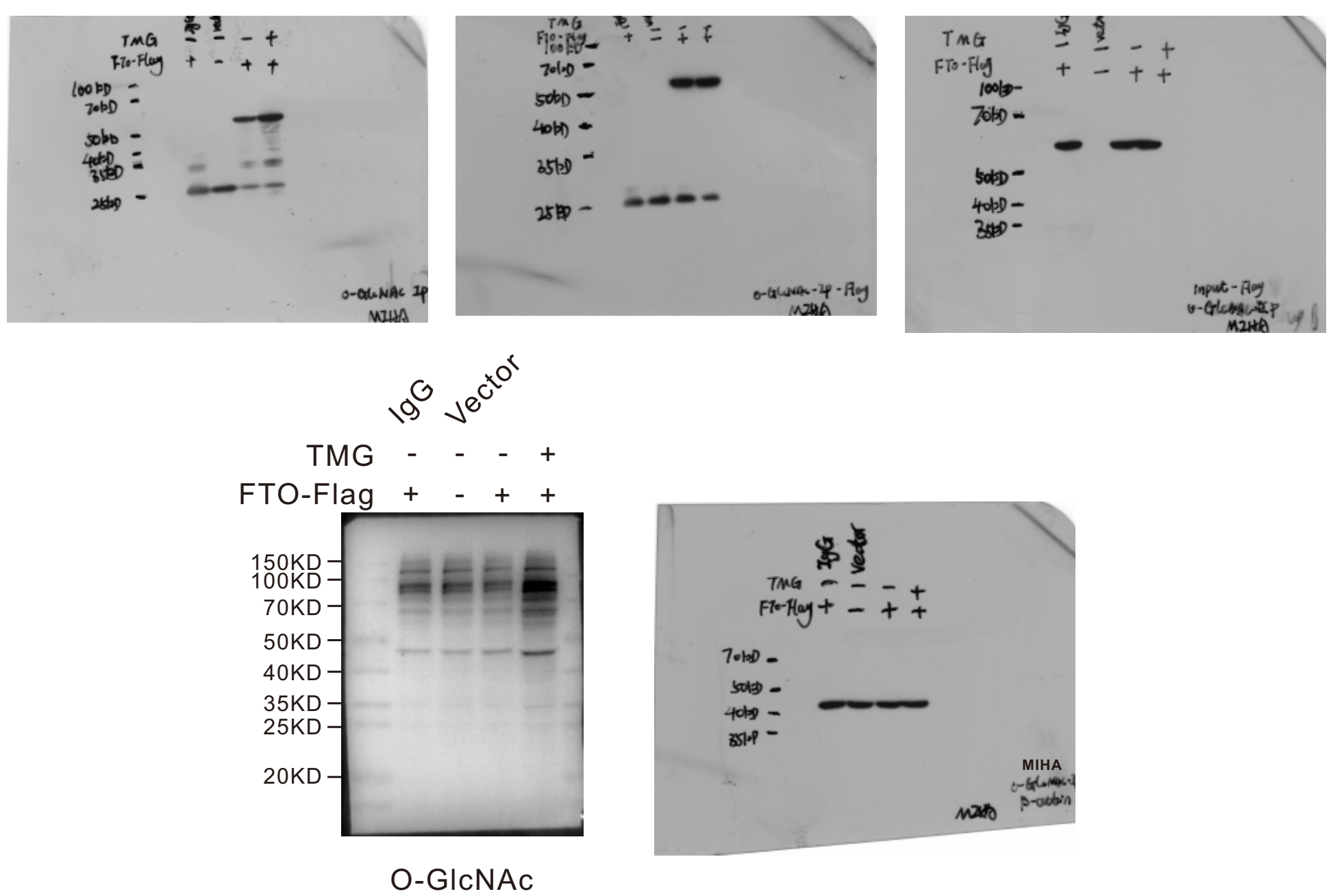

Figure S5D

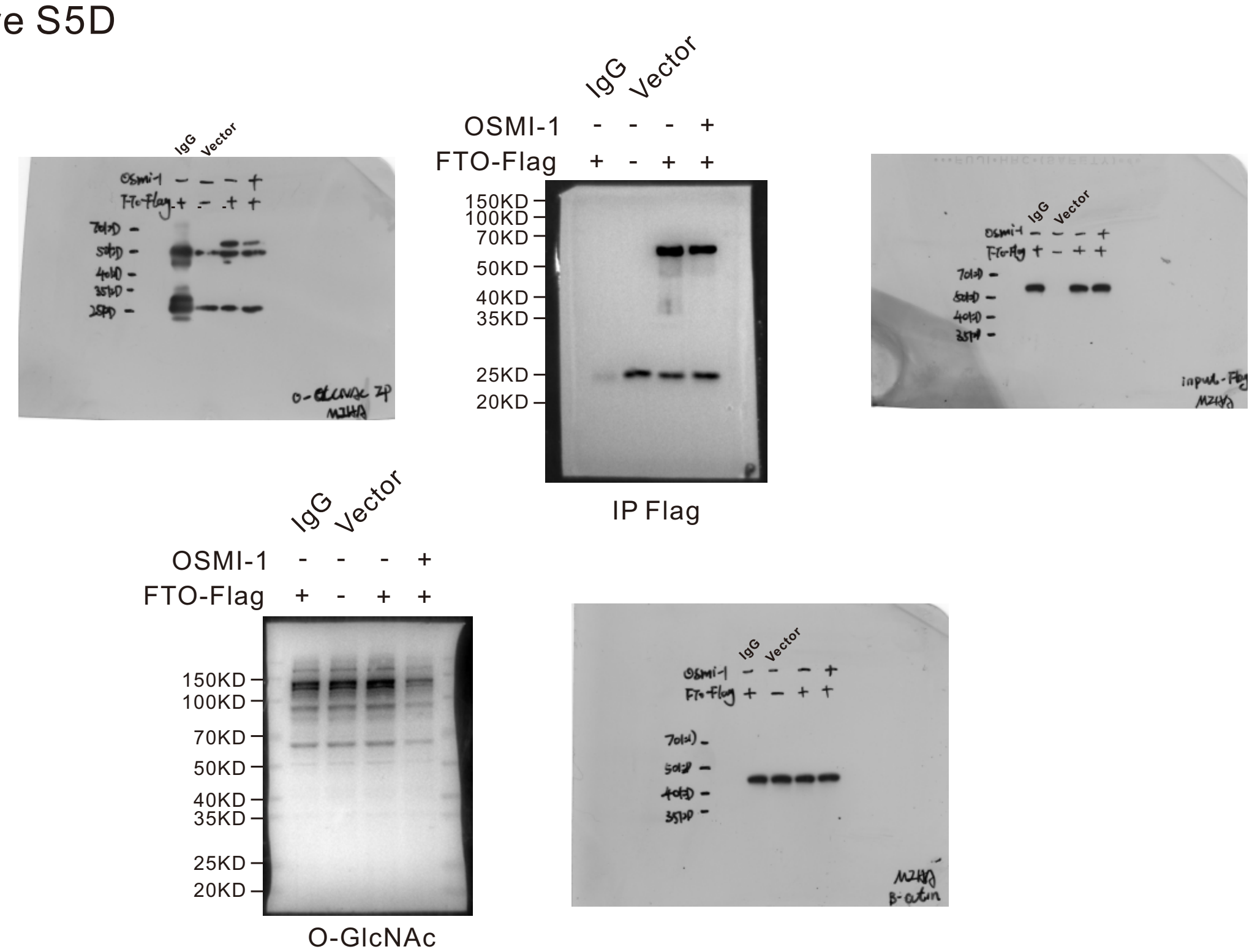

Figure S5E

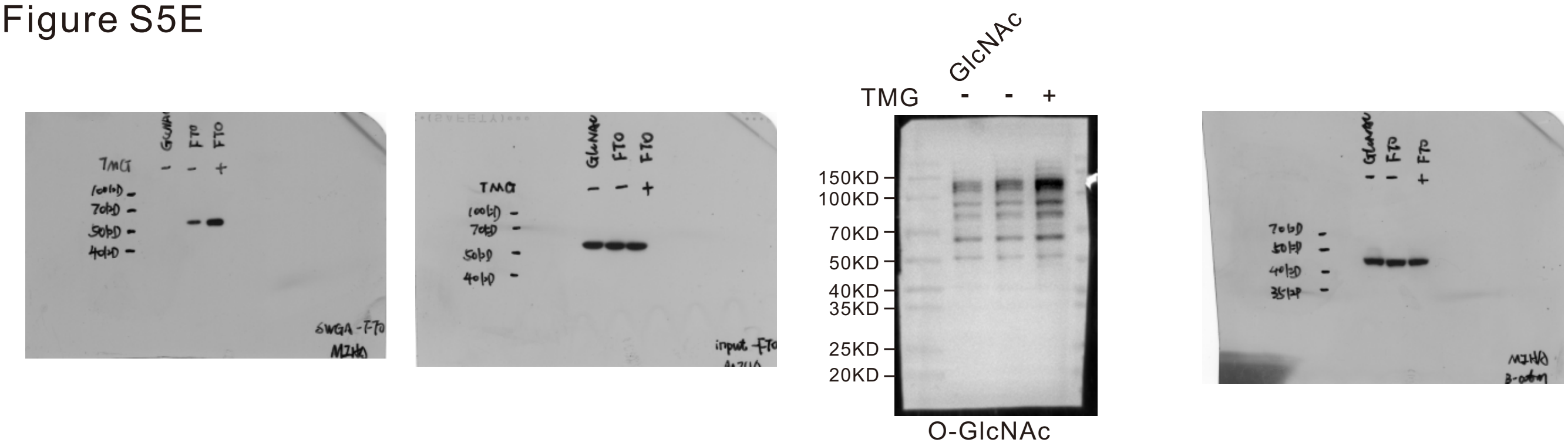

Figure S5F

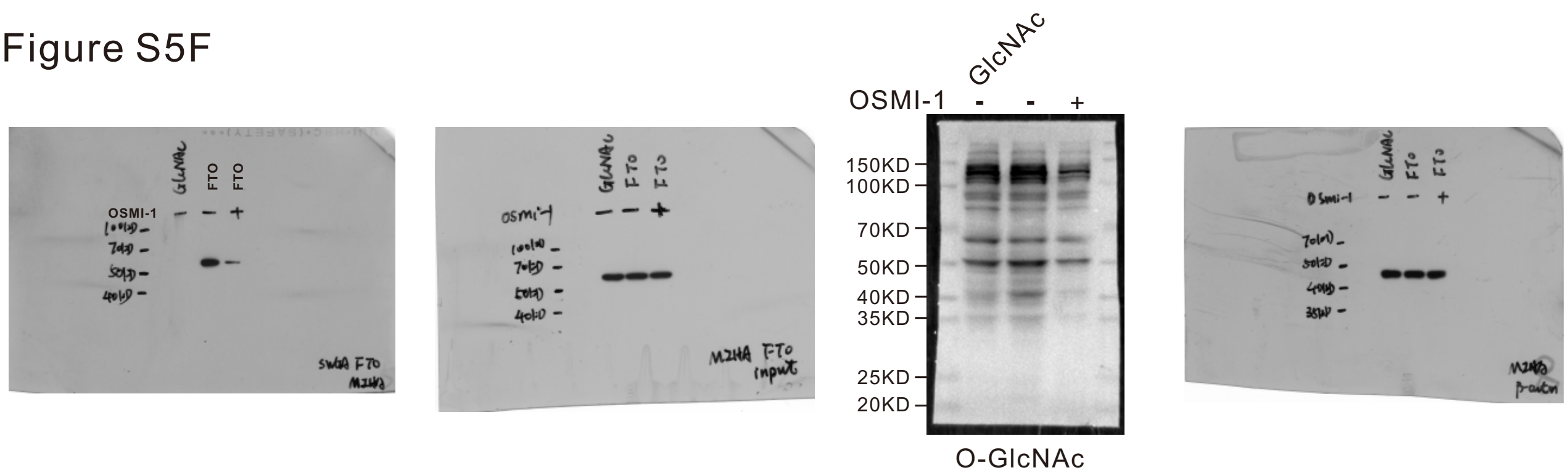

Figure S5G

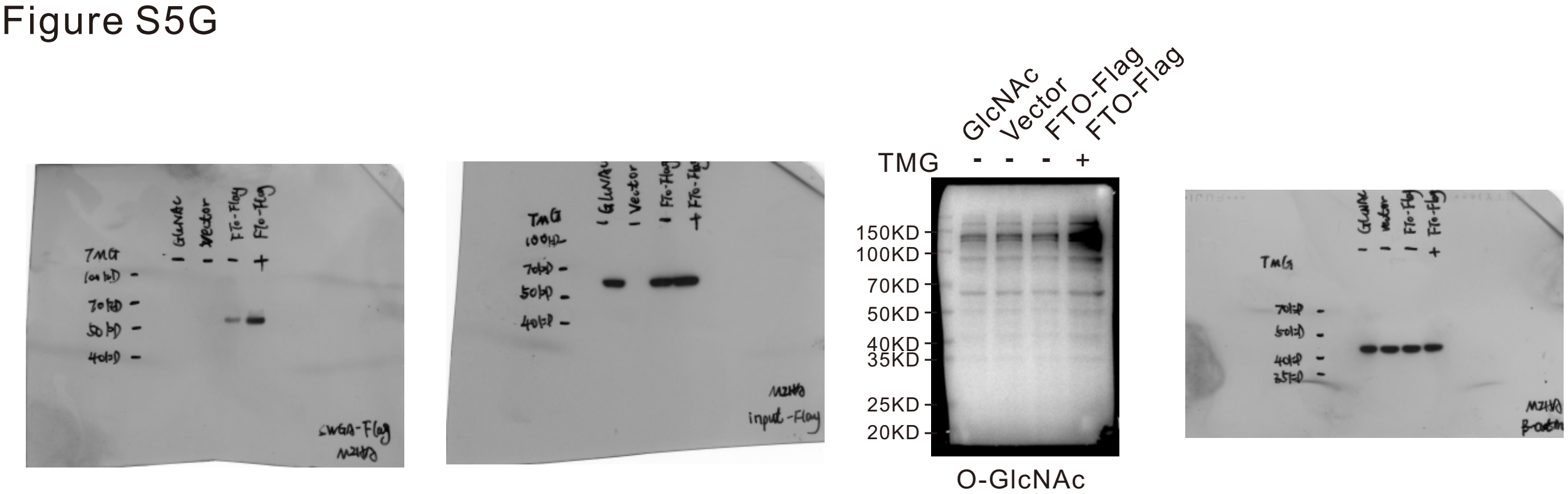

Figure S5H

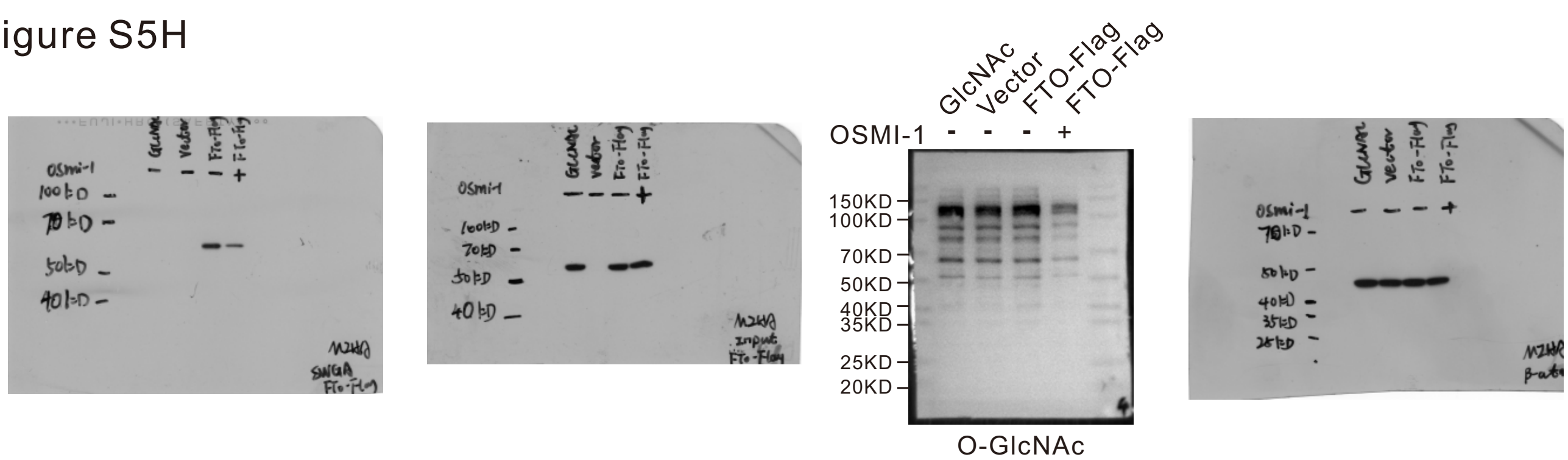

Figure S5I

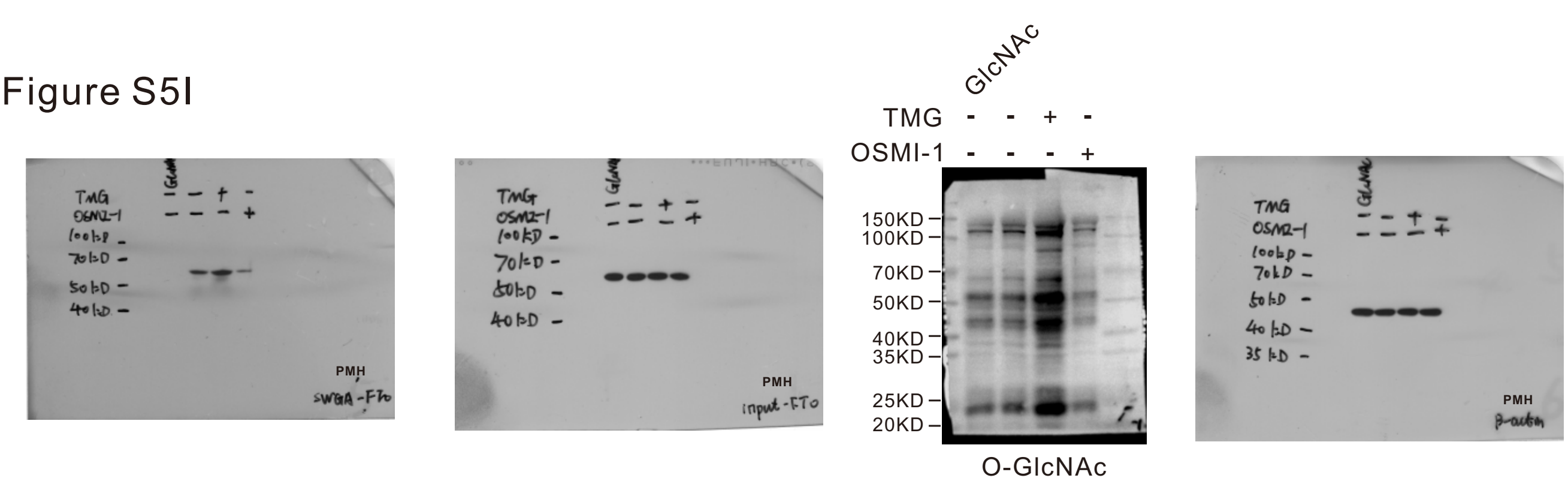

Figure S5J

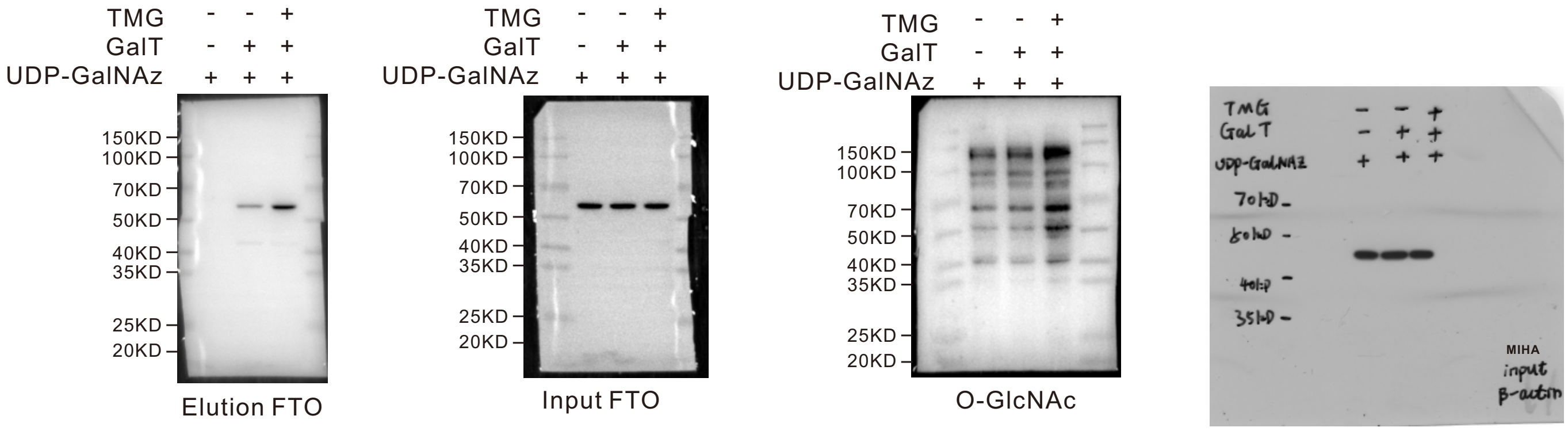

Figure S5K

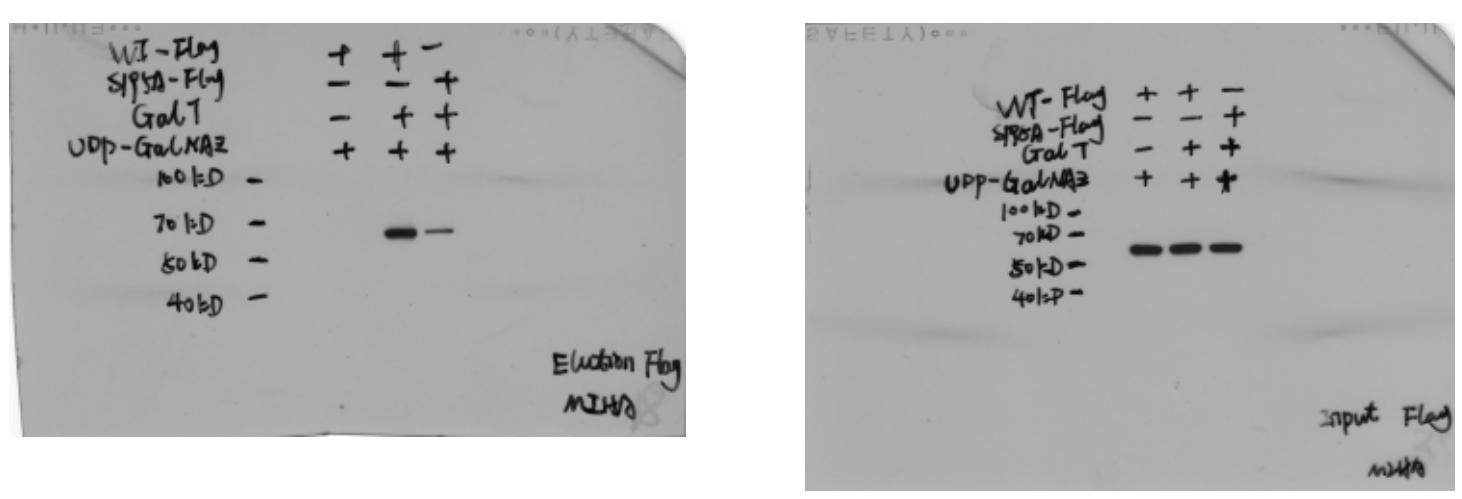

Figure S6A

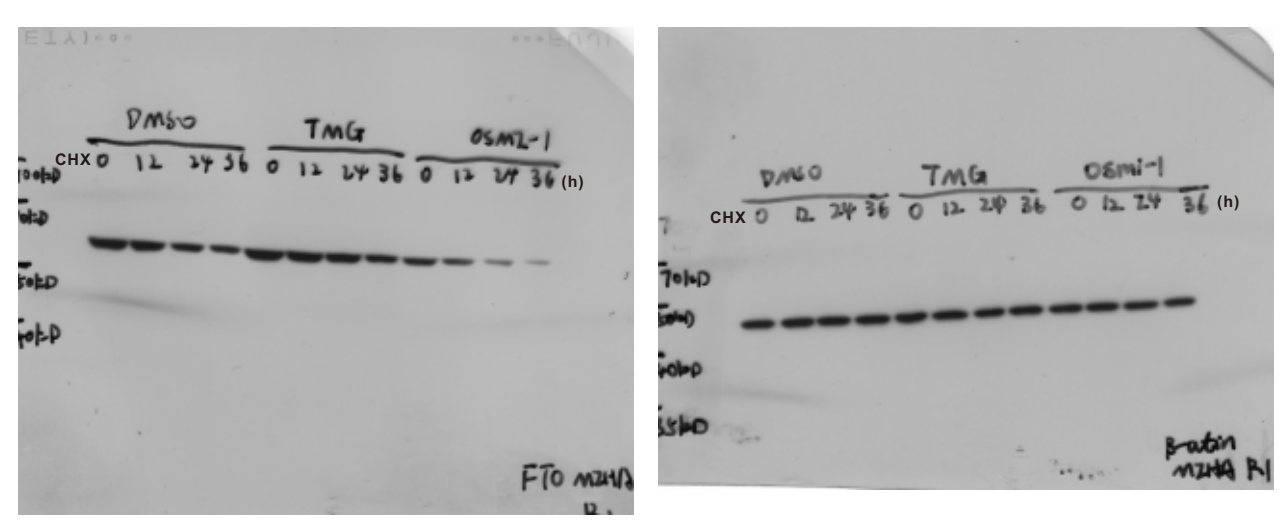

Figure S6B

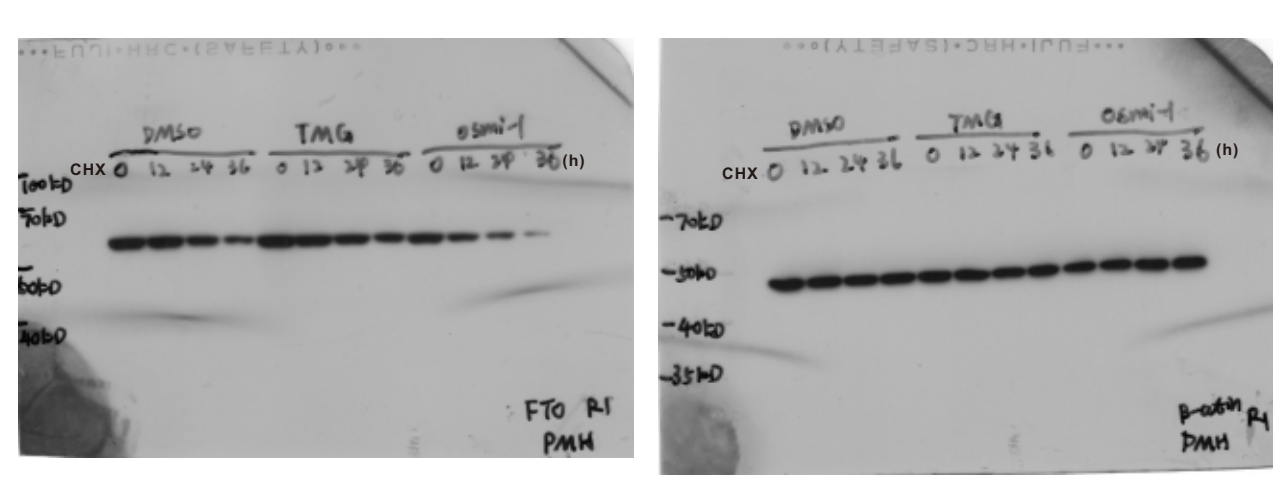

Figure S6C

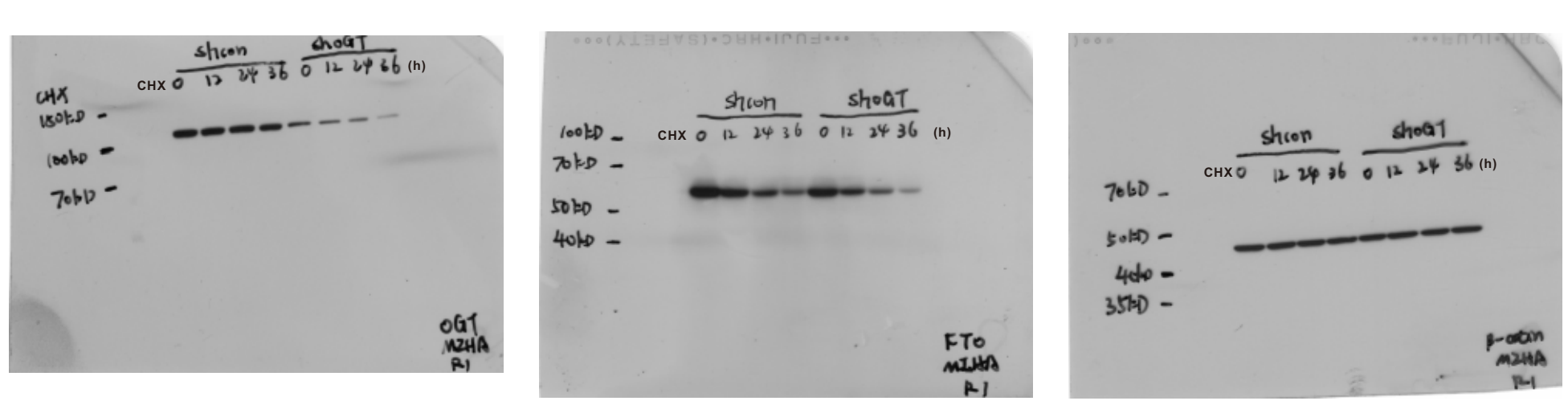

Figure S6D

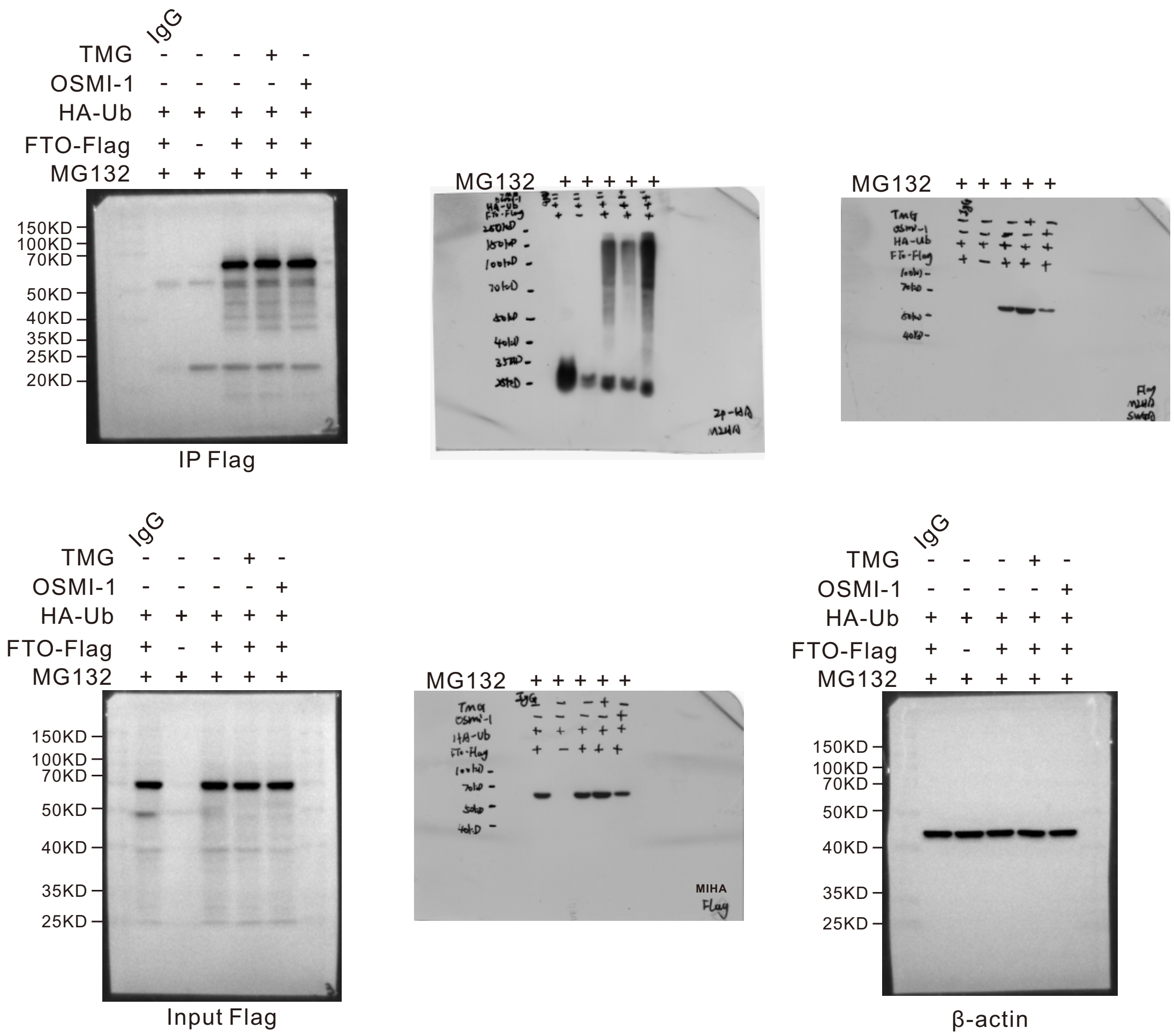

Figure S6E

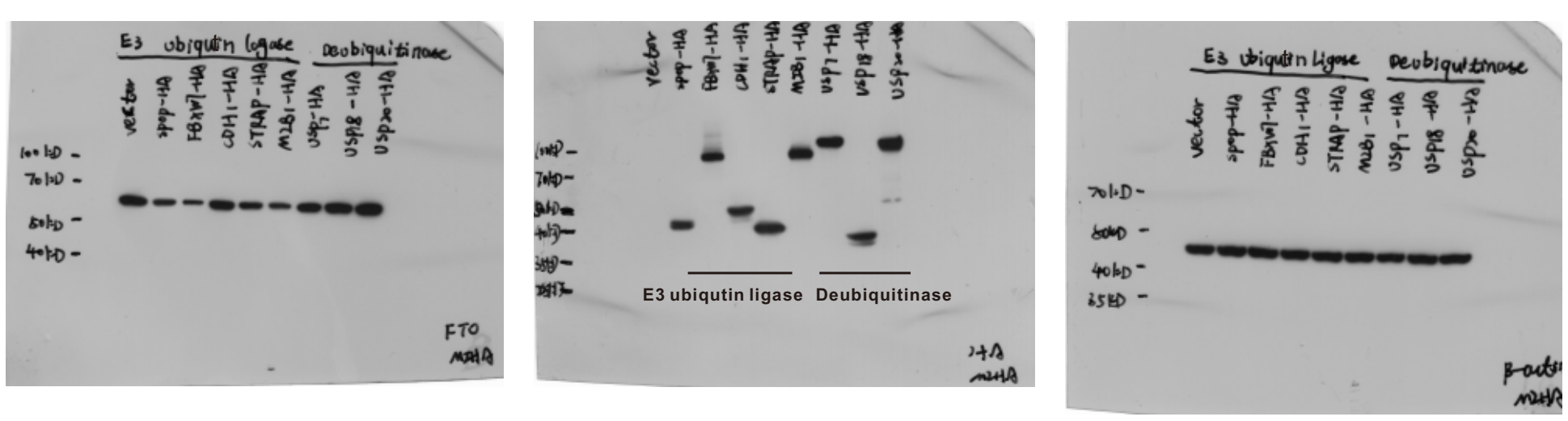

Figure S6F

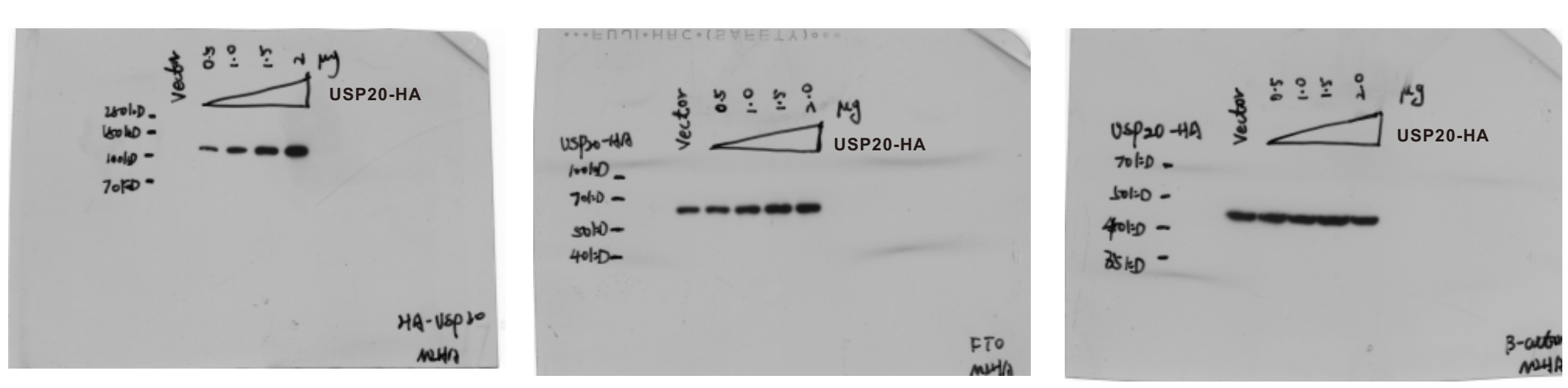

Figure S6G

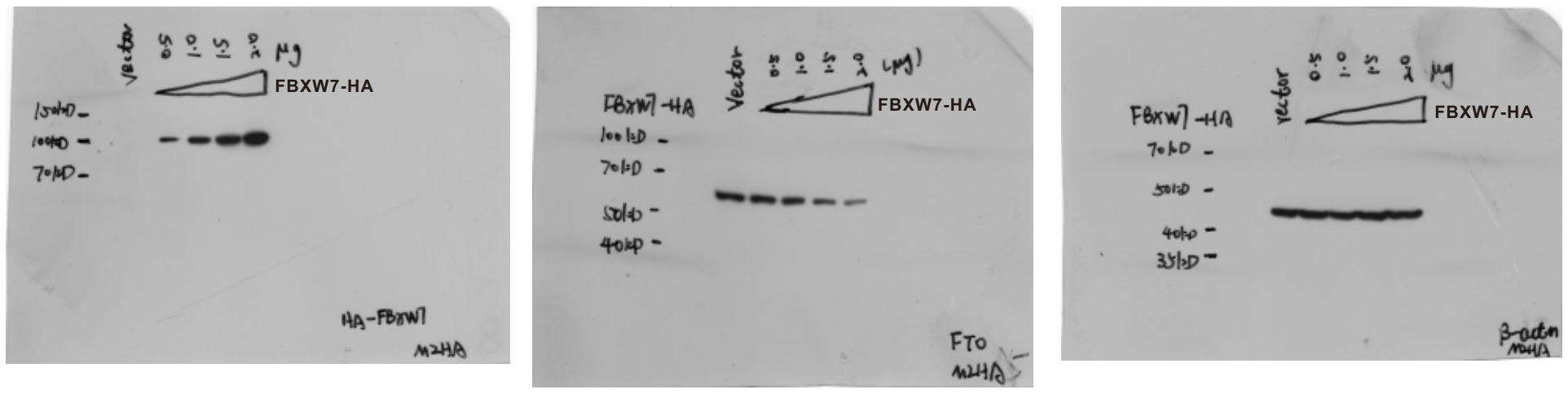

Figure S7B

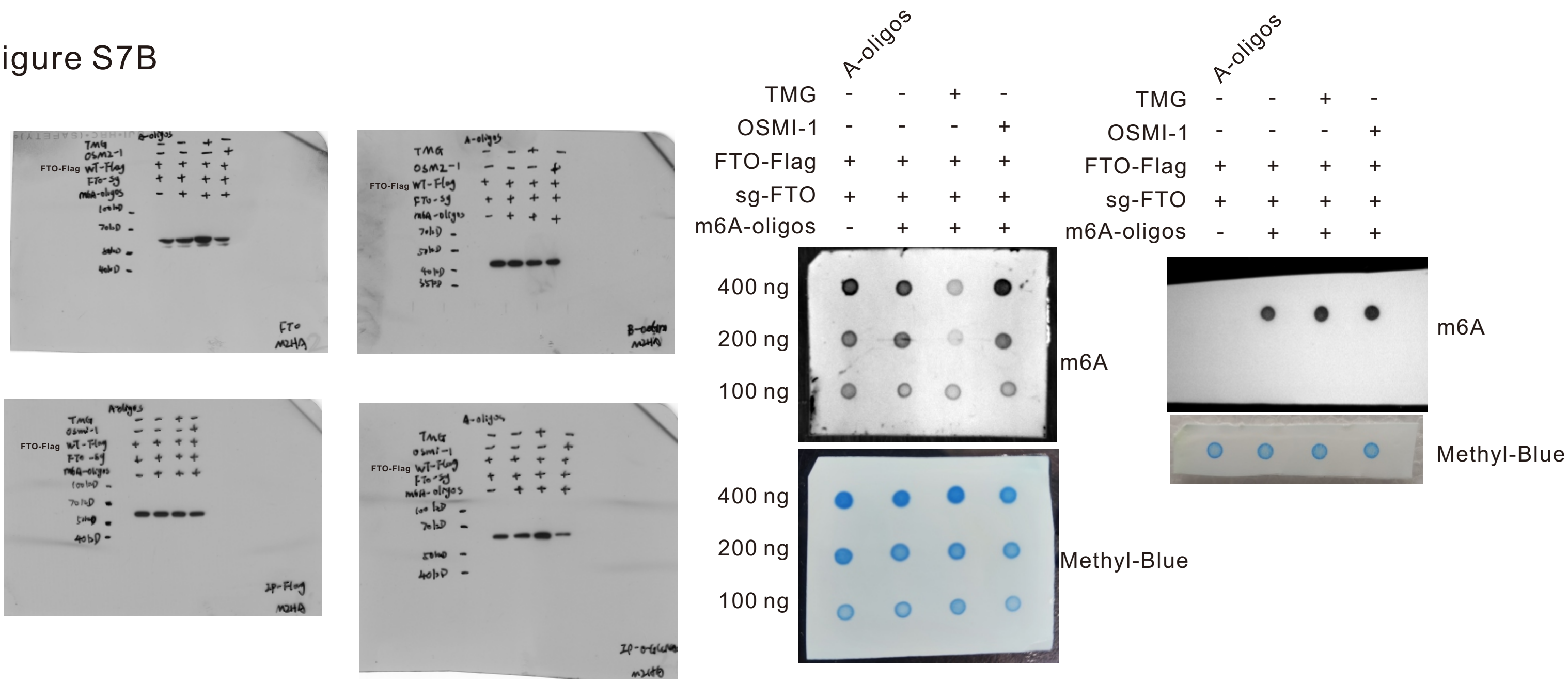

Figure S7C

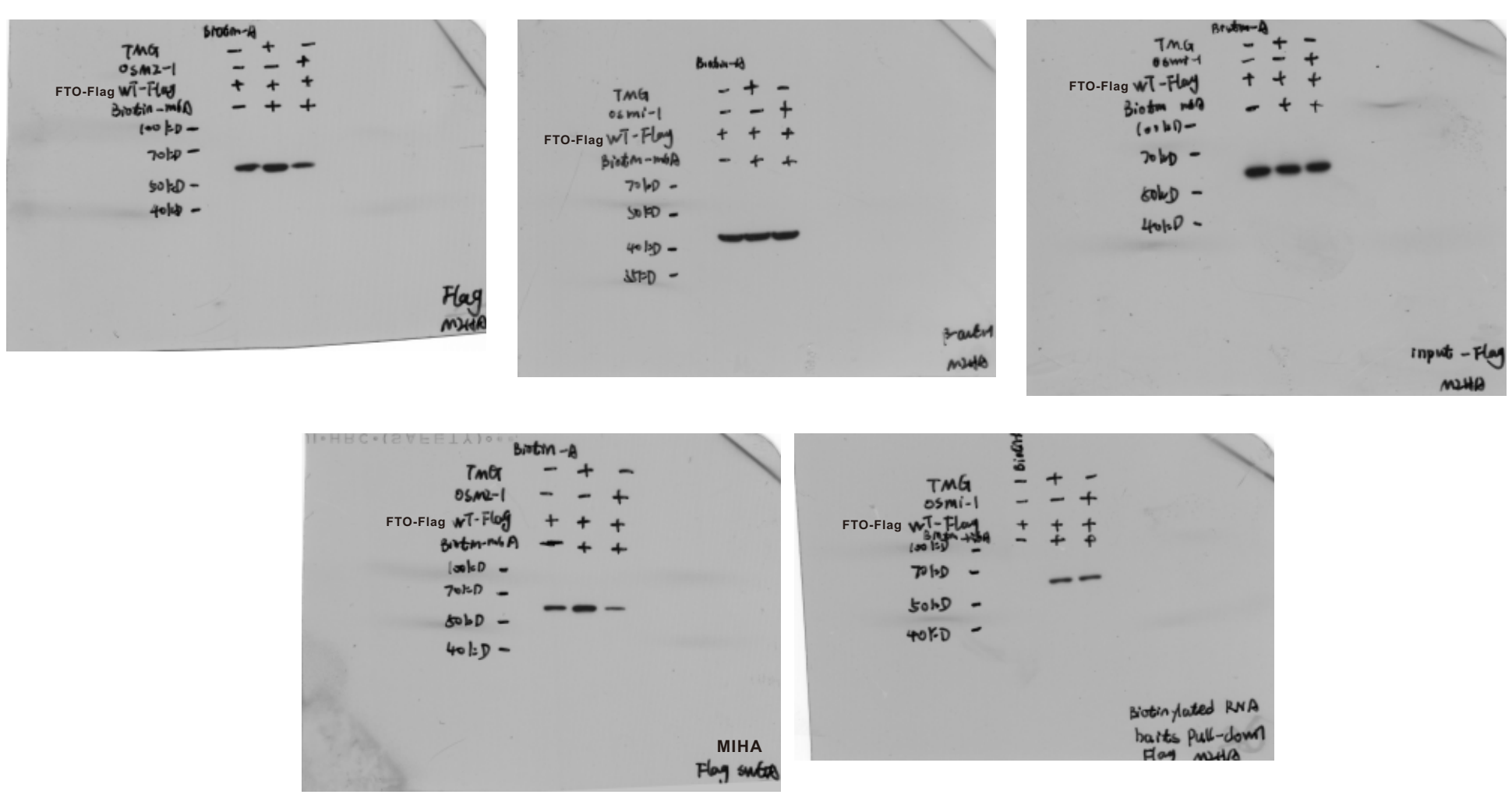

Figure S7D

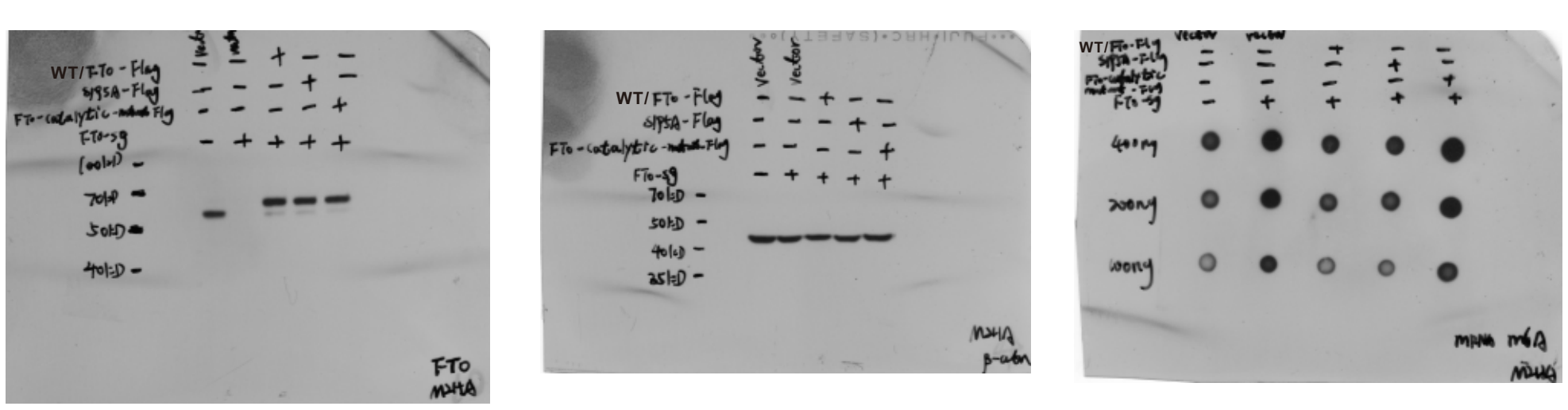

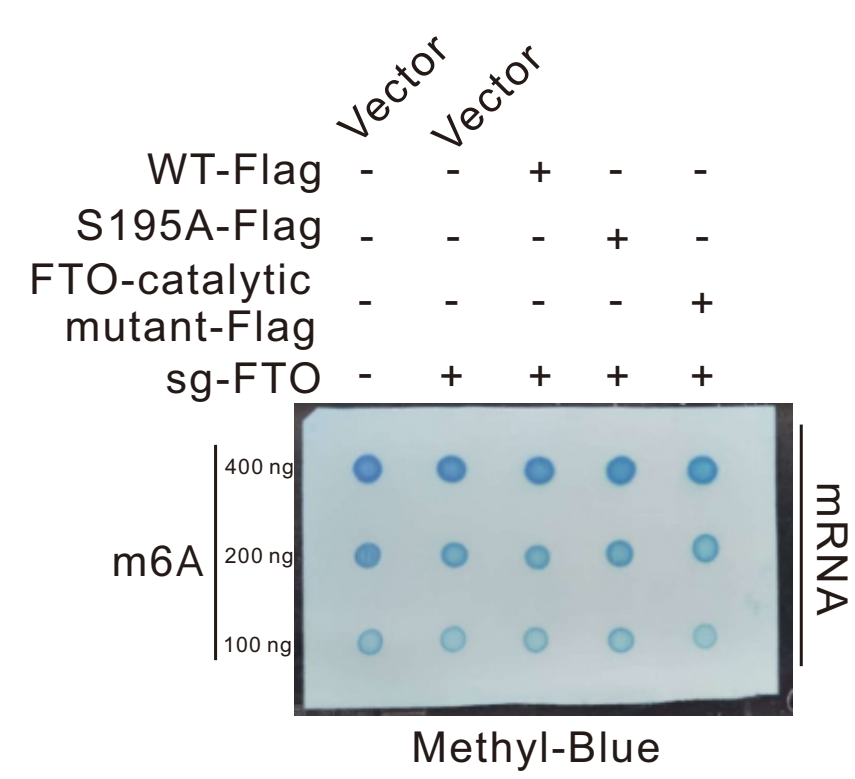

Figure S7E

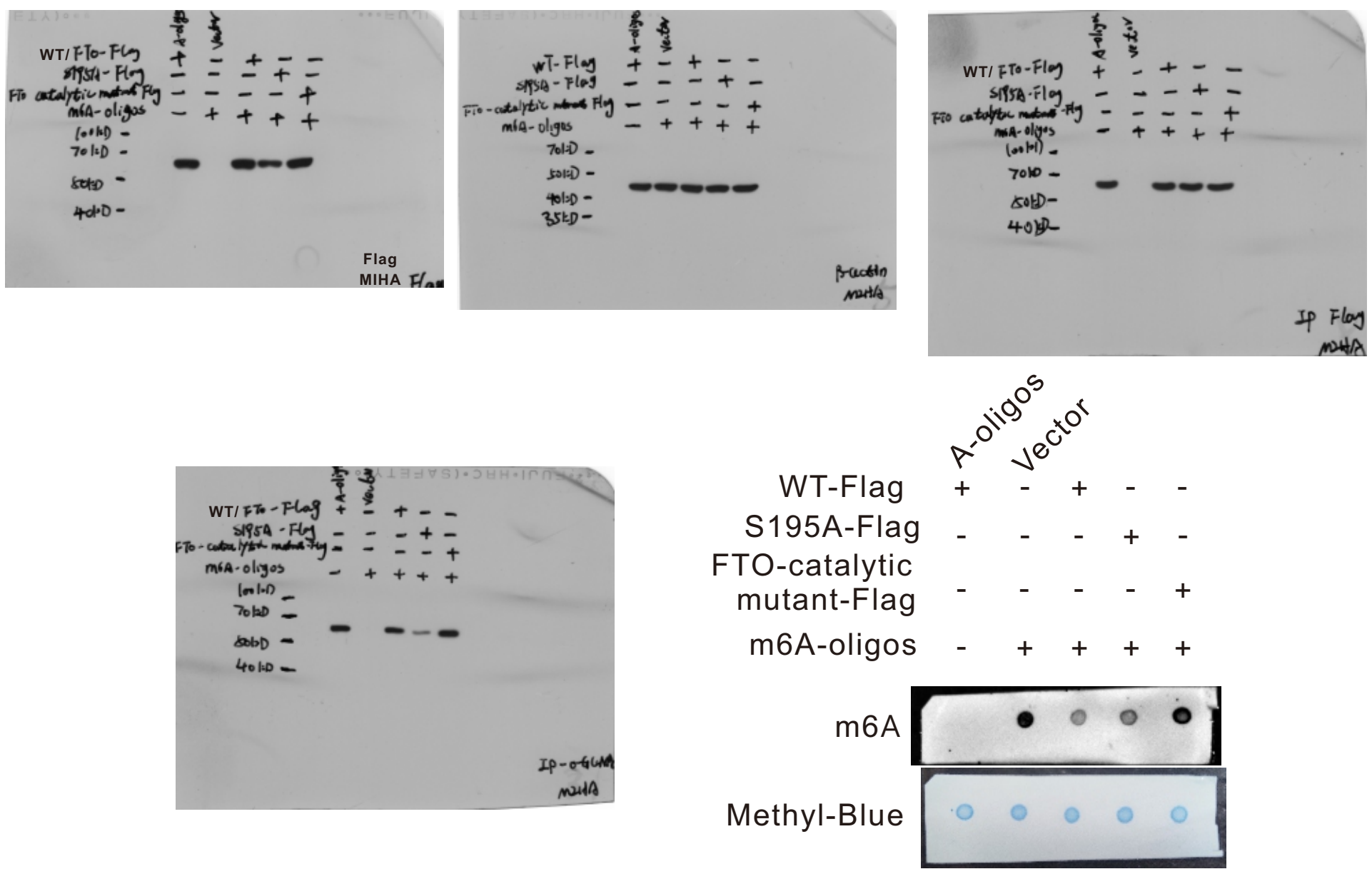

Figure S7F

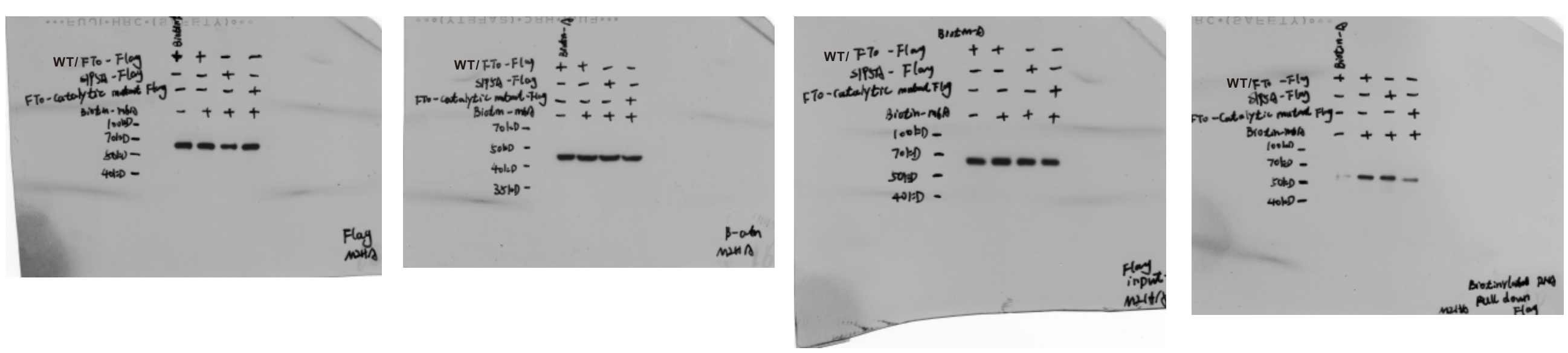

Figure S8A

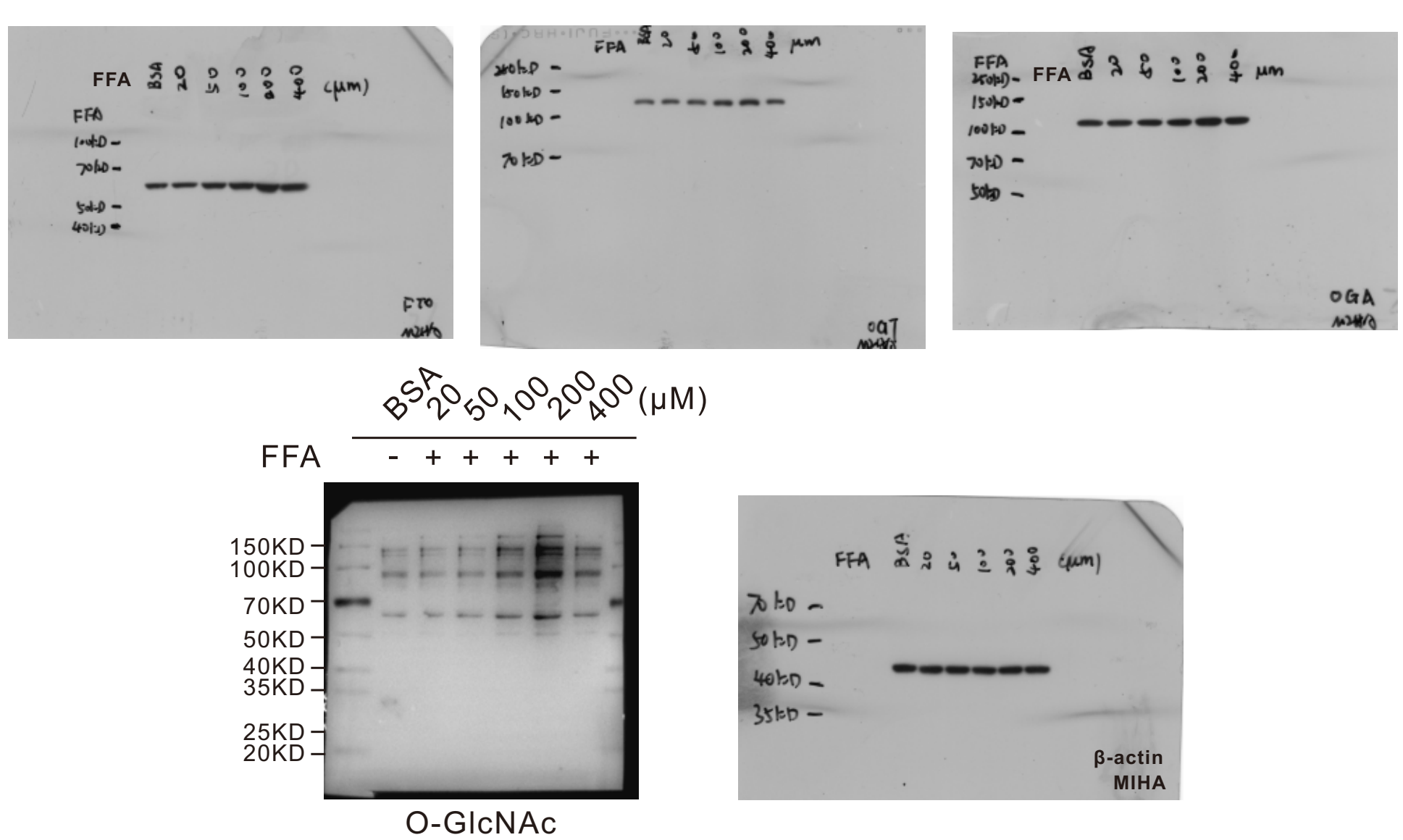

Figure S8C

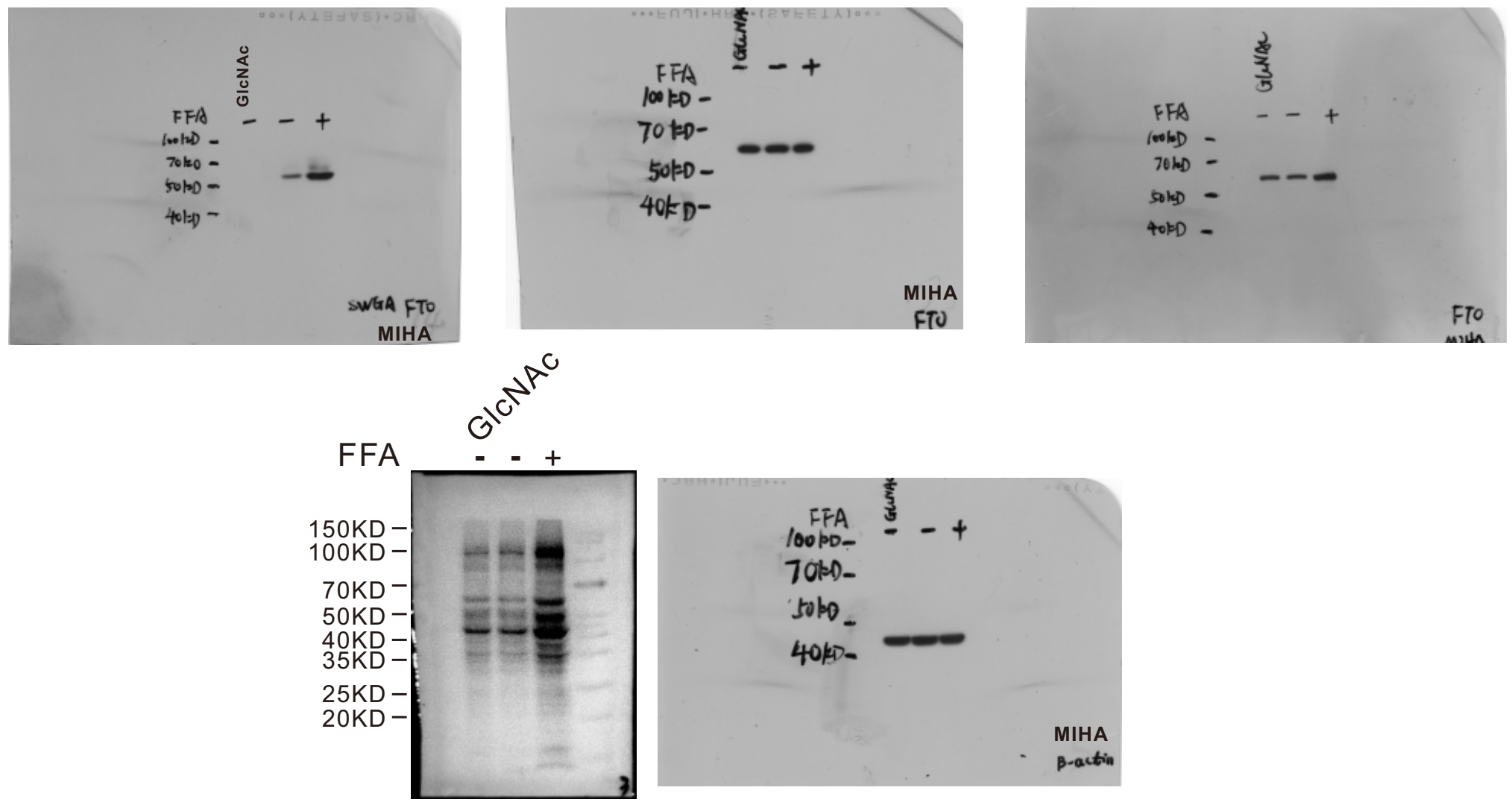

Figure S8D

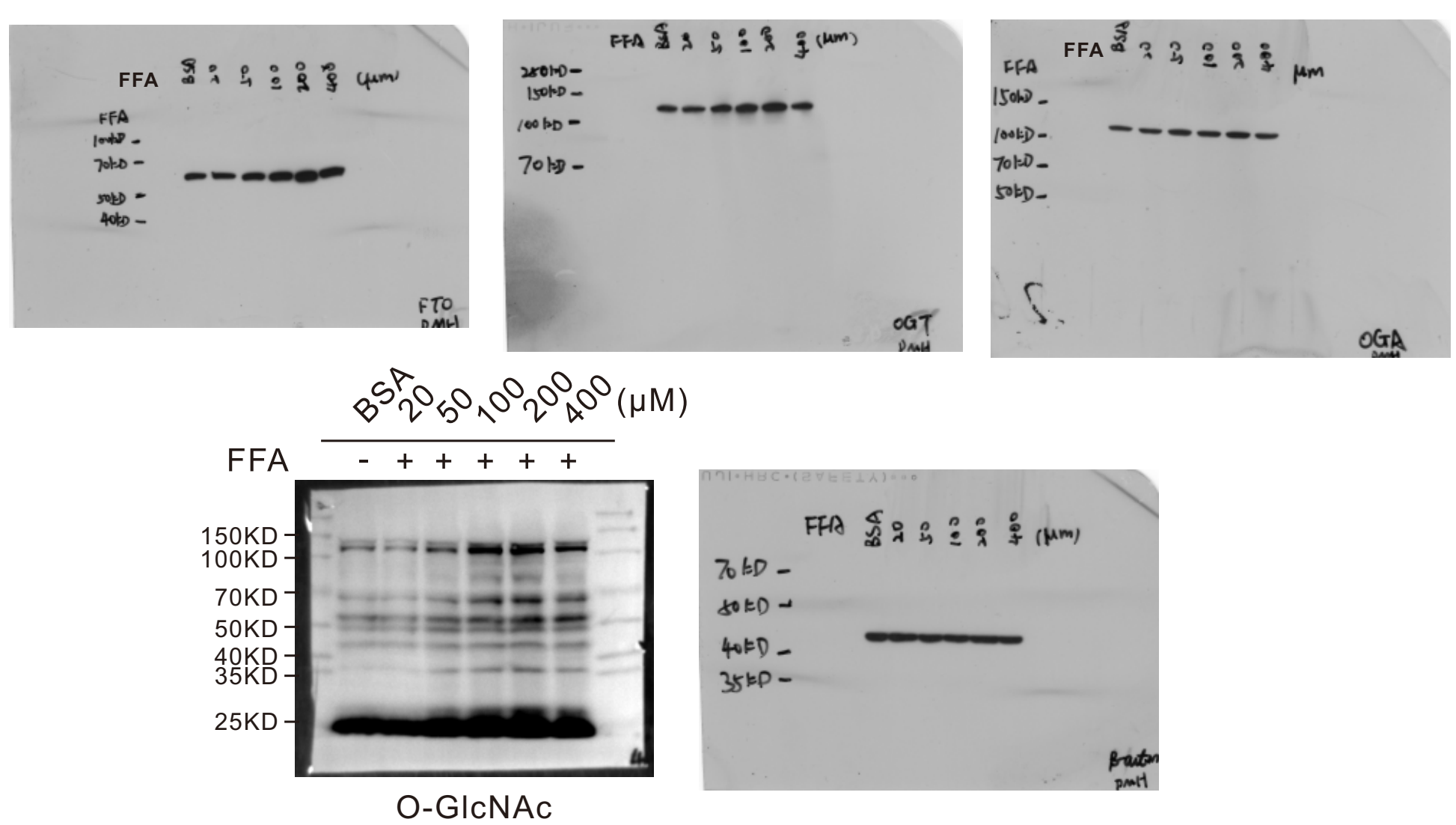

Figure S8F

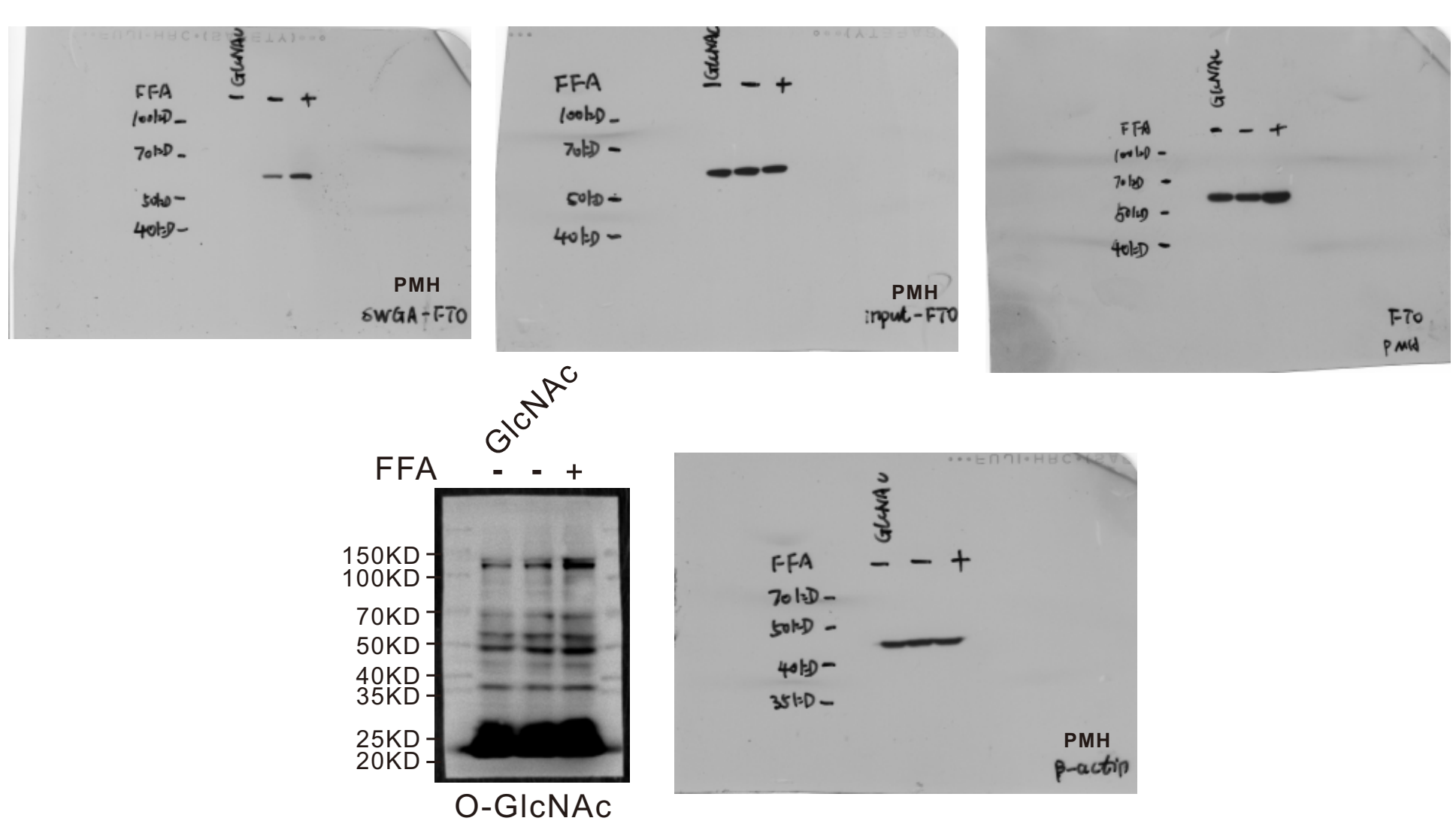

Figure S9G

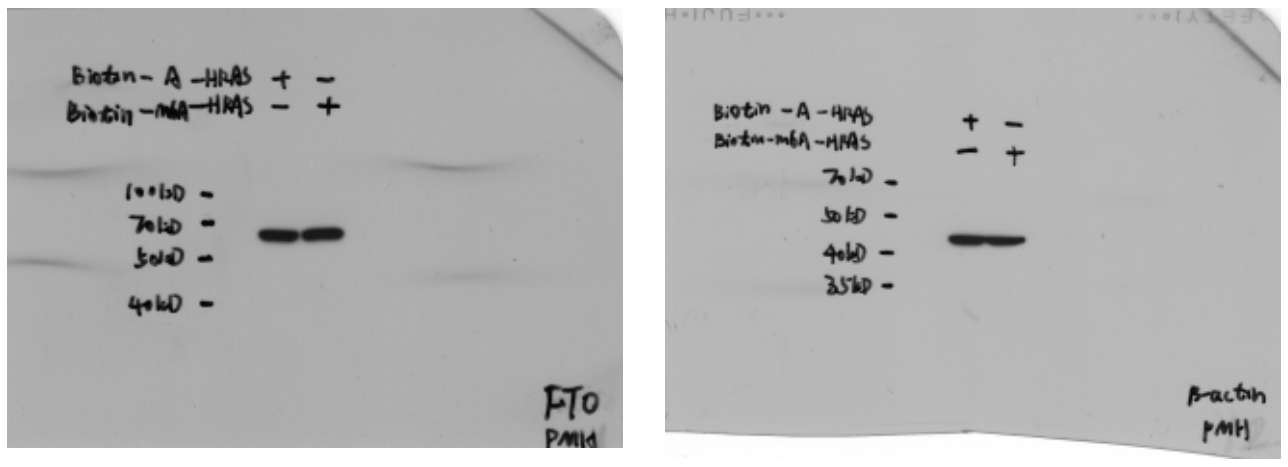

Figure S10A

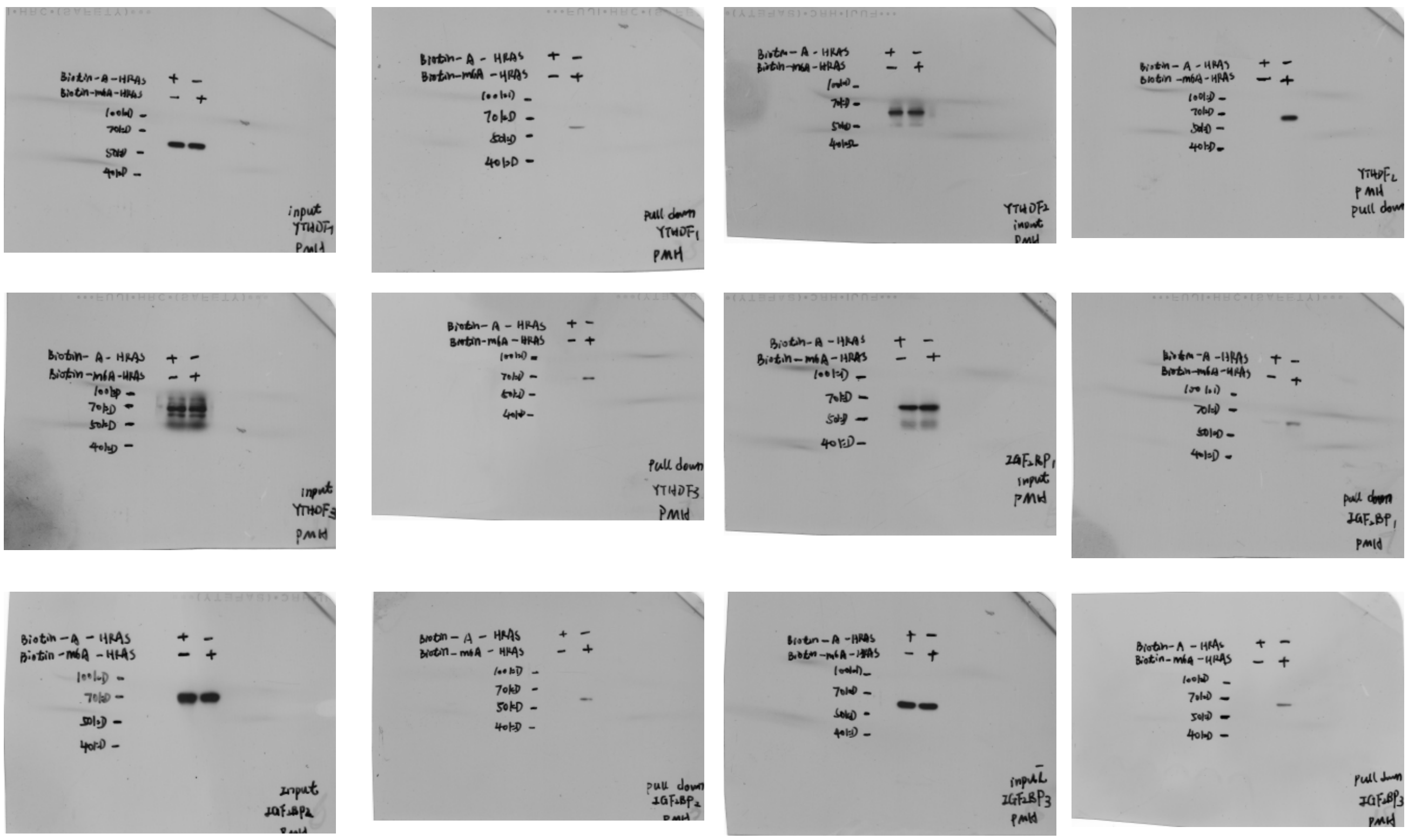

Figure S10C

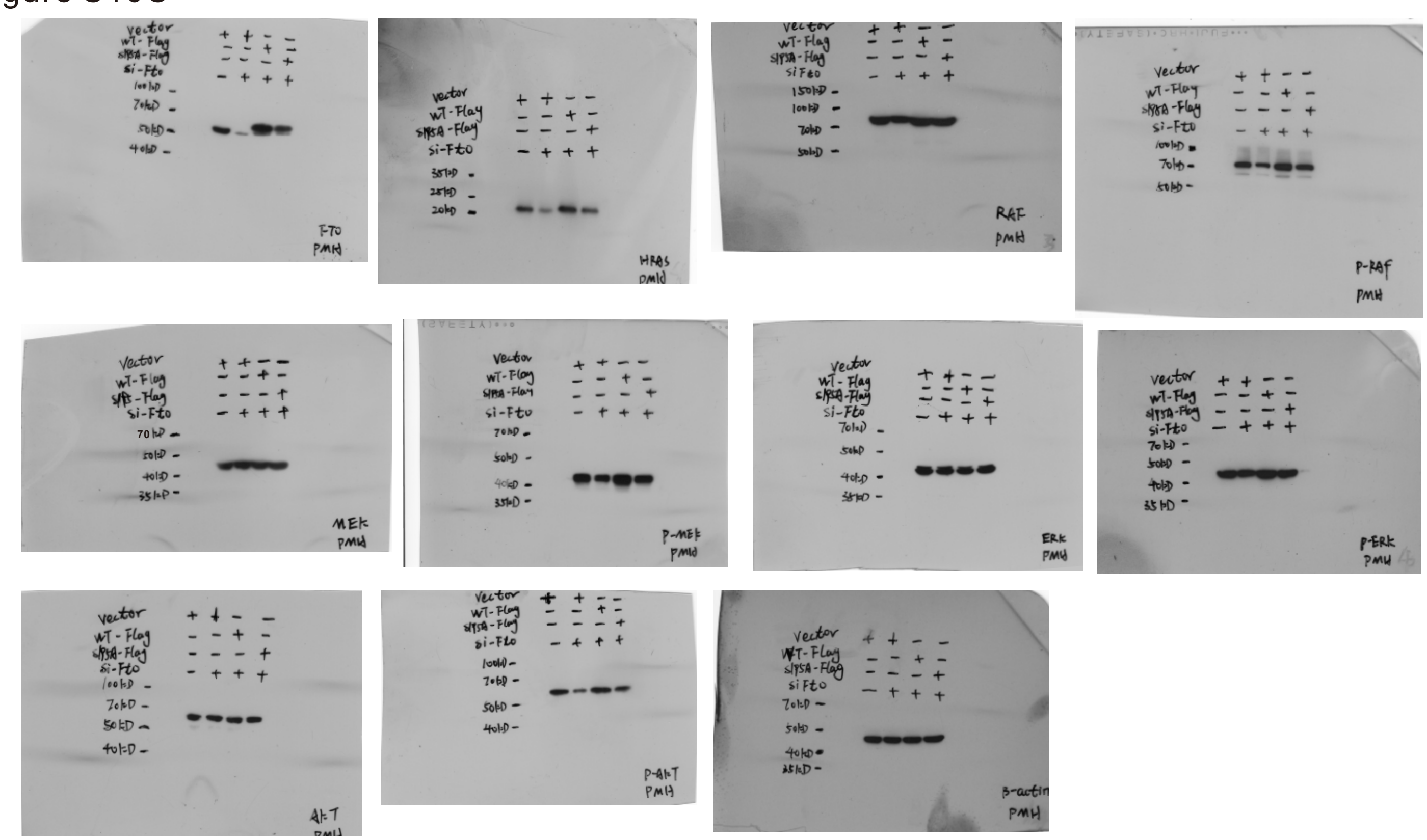

Figure S12B

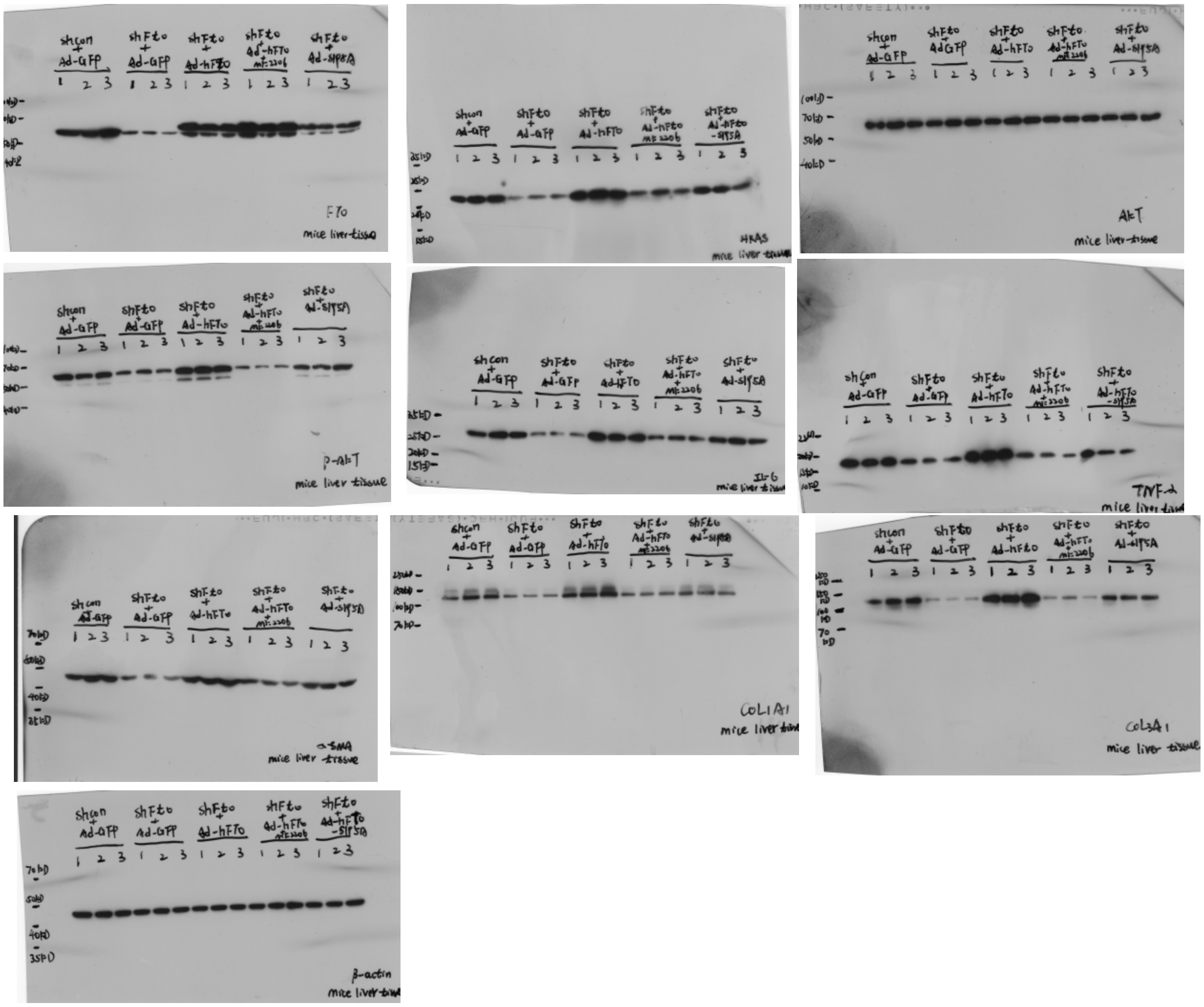

Figure S14A

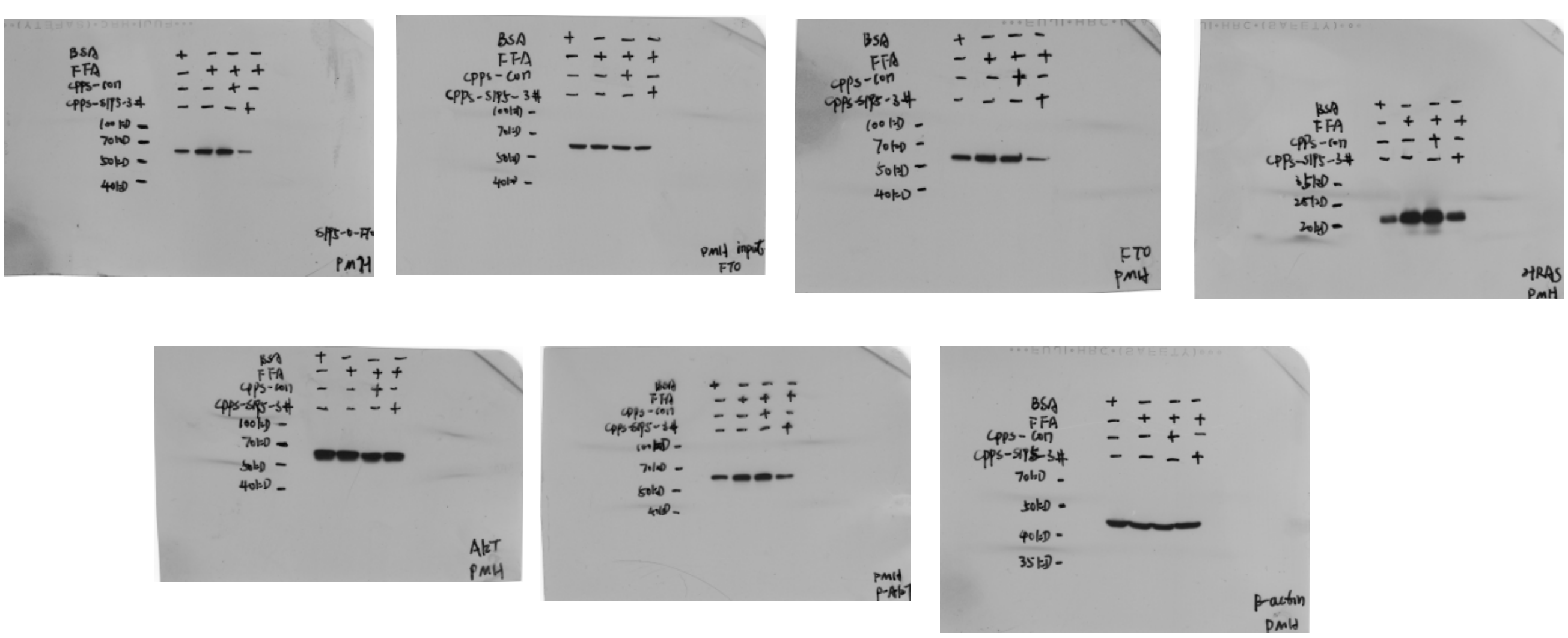

Figure S16B

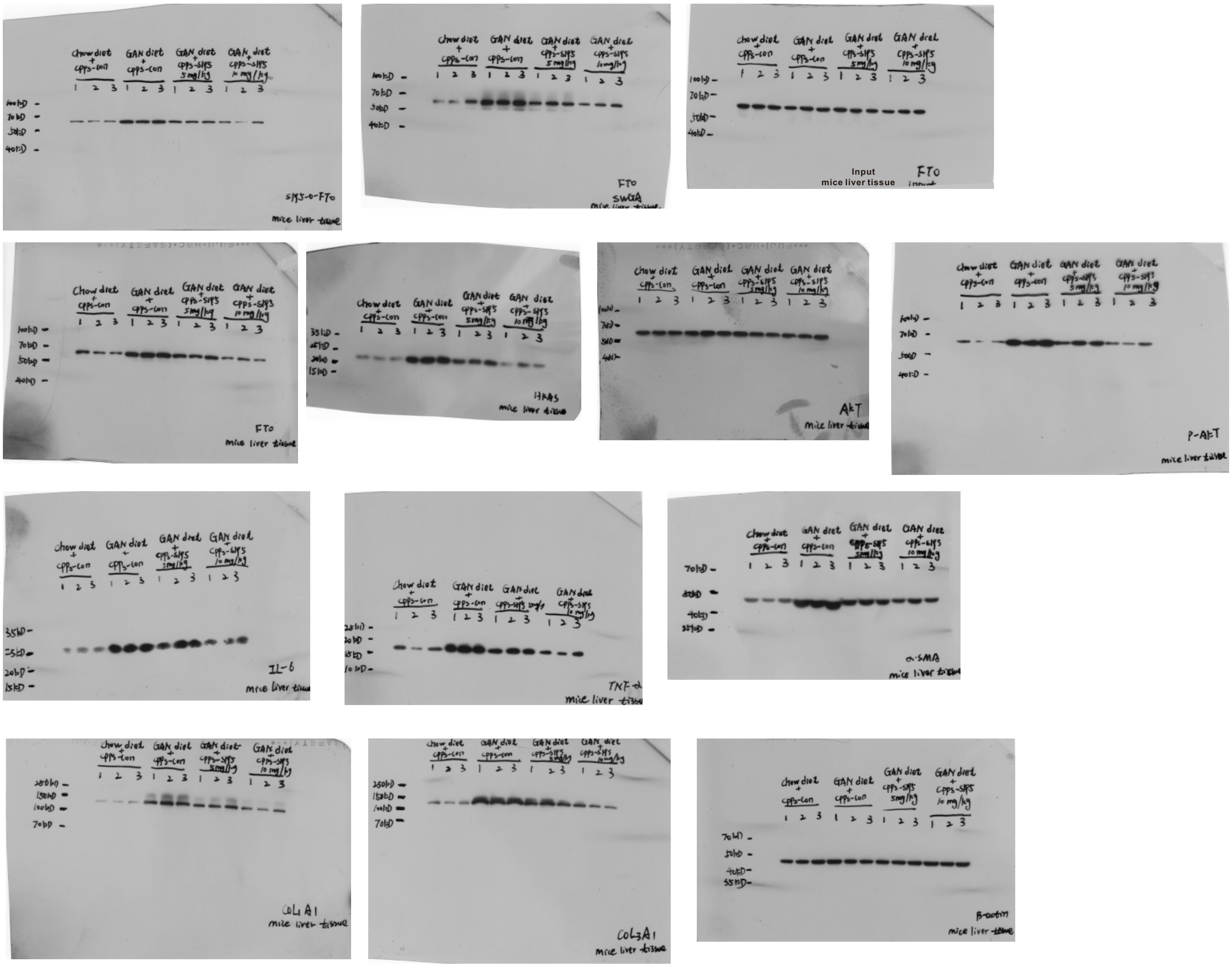
Input
mice liver tissue
FTO
HRAS
AKT
P-AKT
IL-6
α-SMA
COL1A1
COL3A1
β-actin
