# Supplementary material for: Hepatic Lipid Stress Drives Metabolic Dysfunction‐Associated Steatohepatitis Progression through FTO O‐GlcNAcylation‐Induced m6A Demethylation of HRAS
Source: Adv Sci (Weinh). 2026 Oct 11:e78218. Online ahead of print. doi: 10.1002/advs.78218 (PMC13655264; doi:10.1002/advs.78218)
Supplement: Supplementary file 2 [file ADVS-9999-e78218-s001.docx]

**Hepatic Lipid Stress Drives Metabolic Dysfunction-Associated Steatohepatitis Progression Through FTO *O*-GlcNAcylation-Induced m6A Demethylation of *HRAS***

Jia-Xin Yin^1^, Yan-Lai Zhang^1^, Qun Liu^1^, Rui Liu^1^, Hai-Yang Yuan^2,3^, Sui-Dan Chen^4^, Ming-Hua Zheng^2,3, *^, Kai Wang^1, *^, Ni Tang^1, *^, Ai-Long Huang^1, *^

Table of contents

Supplementary Materials and Methods ..........................................................2

Supplementary Figures ...................................................................................9

Supplementary Tables ....................................................................................33

Supplementary References .............................................................................43

**Supplementary Materials and Methods**

**Animal Studies**

Male C57BL/6 mice (6–8 weeks old) were obtained from Beijing Weitong Lihua Experimental Technology Co., Ltd. (Beijing, China). *Pten*^flox/flox^; Albumin-Cre mice (*Pten* L-KO mice) were kindly provided by Prof. Yujun Shi (Sichuan University, Chengdu, China). Genotyping was performed by PCR using tail genomic DNA with the primers listed in Table S1 and 2× Taq Master Mix (E005-01A, Novoprotein, China), followed by agarose gel electrophoresis to confirm the floxed *Pten* allele and Albumin-Cre transgene.

To establish a diet-induced MASH model, C57BL/6 mice were fed a GAN diet (Research Diets, D09100310, 40% fat, 22% fructose and 2% cholesterol) with drinking water containing 23.1 g/L fructose and 18.9 g/L glucose for 24 weeks. Control mice were fed a normal chow diet (Research Diets, D09100304) with normal water. Additionally, 40-week-old *Pten* L-KO mice, which spontaneously develop MASH-like pathology, were used as a genetic MASH model as previously reported.^[1]^

For CPPs treatment, male C57BL/6 mice on the GAN diet received intraperitoneal injections of CPPs (5 or 10 mg/kg in PBS) or vehicle control twice per week from week 14 to week 28 of dietary intervention.

**Cell Culture and Treatment**

The human hepatocyte cell line MIHA, kindly provided by Dr Ben C.B. Ko (The Hong Kong Polytechnic University, Hong Kong, China), was cultured in RPMI-1640 medium (11875093, Gibco, CA, USA) supplemented with 10% FBS (10091148, Gibco, MA, USA) and 1% penicillin/streptomycin (HY-K1006, MCE, NJ, USA) at 37°C in 5% CO_2_. Cells were treated with varying concentrations of a free fatty acid (FFA) mixture (OA:PA ratio, 2:1, Sigma-Aldrich, MO, USA) for 24 h; 25 µM TMG (T6056, TargetMol, MA, USA) or 20 µM OSMI-1 (T16409, TargetMol) for 12 h; 10 µM MK2206 (T1952, TargetMol) for 24 h; 10 µM FB23-2 (T7138, TargetMol) for 24 h.

**Isolation and Culture of Primary Mouse Hepatocytes**

Primary hepatocytes were isolated and cultured from the livers of 8- to 10-week-old male C57BL/6 mice as described previously^[2]^. Briefly, mice livers were perfused with D-Hank’s buffer (14170112, Gibco) followed by digestion buffer (0.55 mg/mL collagenase, V900893, Sigma-Aldrich). Then, the digested mouse liver was mechanically disrupted to release the hepatocytes, which were filtered, washed, and cultured with DMEM (L170KJ, BasalMedia, Shanghai, China) supplemented with 10% FBS, 1% penicillin/ streptomycin at 37 °C in 5% CO_2_.

**Construction of Plasmid, ShRNA, Lentivirus, Adenovirus**

Full-length cDNAs of *FTO, YTHDF2, HRAS, OGT, SPOP, CDH1, STRAP, MIB1, USP18* and *FBXW7* were amplified using PCR. The *FTO* cDNA was inserted into the pAdTrack-3Flag-TO4 (gift from Dr. Tong-Chuan He, University of Chicago, USA) and pSEB-3Flag vectors; *YTHDF2* cDNA was inserted into the pSEB-3Flag vector; *HRAS*, *OGT*, *SPOP*, *CDH1*, *STRAP*, *MIB1*, *USP18* and FBXW7 cDNAs were inserted into pBudCE-3HA vectors. The plasmids of pCMV-USP7-3HA-Neo (Cat: P53761, MIAOLING BIOLOGY, Wuhan, China) and pLVX-Puro-HA-USP20 (Cat: P20842, MIAOLING BIOLOGY, Wuhan, China) were purchased from commercial sources.

For RNA interference, small hairpin RNAs (shRNAs) targeting *OGT*, *YTHDF2*, and *HRAS* were designed and inserted into the pLL3.7 lentiviral vector (gift from Prof. Bing Sun, Chinese Academy of Sciences, Shanghai, China). Lentiviral particles were generated by co-transfecting HEK293T cells with shRNA constructs, psPAX2, pMD2.G and Lipofectamine 8000 (C0533, Beyotime, Shanghai, China). As previously described,^[3]^ an FTO-knockout lentivirus was constructed using the CRISPR/Cas9 system (gift from Dr. Ding Xue, Tsinghua University, Beijing). Supernatants containing viral particles were collected at 48 h post-transfection, filtered, and used to infect MIHA cells in the presence of 3 μg/mL polybrene (107689, Sigma-Aldrich).

Recombinant adenoviruses expressing wild-type FTO (AdFTO-WT) and the S195A mutant (AdFTO-S195A) were generated using the AdEasy system as described previously.^[3]^ An adenovirus expressing GFP (AdGFP) served as a control. All relative primer sequences are listed in Table S1.

**Glucose and Insulin Tolerance Tests**

For glucose tolerance tests (GTT), mice were fasted for 16 h and then administered glucose (2 g/kg body weight) via intraperitoneal injection. For insulin tolerance tests (ITT), mice were fasted for 6 h before receiving intraperitoneal insulin (0.75 U/kg body weight). Blood glucose levels were measured at 0, 30, 60, 90 and 120 minutes post-injection using a glucose meter (Accu-Chek Active, Roche, Basel, Switzerland).

**Metabolite Extraction and Detection**

Liver tissues were snap-frozen in liquid nitrogen and stored at -80°C before analysis. For metabolite extraction, 30 mg of liver tissue was homogenized in 400 μL pre-cooled methanol/acetonitrile solution (1:1, v/v), followed by vortexing, sonication for 30 min at 4°C, and centrifugation at 14000 g at 4°C for 20 min. The supernatant (100 μL) was subjected to liquid chromatography–mass spectrometry (LC-MS).

Untargeted metabolomics profiling of liver samples from GAN diet-induced MASH and control mice was performed by Shanghai Applied Protein Technology (Shanghai, China) using ultra high-performance liquid chromatography (Agilent 1290 Infinity LC) coupled with quadrupole time-of-flight mass spectrometry (UHPLC-QTOF/MS).

For targeted quantification of UDP-GlcNAc, metabolites from mouse liver tissues or cell samples were extracted as described above. The supernatant (100 μL) was separated using an Agilent 1290 Infinity LC ultra-high-performance liquid chromatography (UHPLC) HILIC column (Agilent Technologies, USA) and analyzed using an AB TripleTOF 6600 mass spectrometer (AB SCIEX, USA).

***O*-GlcNAcylation 4D-Label Free Quantitative Proteomic**

Liver tissues from MASH and control mice were sent to Shanghai Applied Protein Technology (Shanghai, China) for analysis. Briefly, proteins were extracted, digested into peptides with trypsin, and incubated overnight at 4°C with anti-GlcNAc-S/T antibody beads (PTMScan® *O*-GlcNAc [GlcNAc-S/T] Motif Kit, Cell Signaling Technology). The beads were washed with chilled IAP and water for 3 times, and desalted using Zip Tips. Finally, the samples were separated and identified by LC-MS/MS analysis.

**Ubiquitination Assay**

MIHA cells were co-transfected with HA-Ub and the Flag-tagged FTO (WT or S195A) plasmids. To inhibit proteasomal degradation, cells were treated with 15 μM MG132 (T2154, TargetMol) for 10 h before harvesting. Cells were lysed in SDS lysis buffer (50 mM Tris-HCl; 150 mM NaCl; 1% SDS; pH 7.5) and boiled to denature proteins. The supernatants were incubated with anti-Flag (F3165, Sigma-Aldrich) at 4°C overnight, followed by incubation with protein A/G magnetic beads (HY-K0202, MCE) for 4 hours. The immunoprecipitated complexes were eluted, denatured, and analyzed by immunoblotting with the anti-HA antibody (26183, Invitrogen, CA, USA).

**Co-immunoprecipitation (co-IP)**

Cells were harvested with lysis buffer containing 1x protease inhibitor and 1x phosphatase inhibitor. After vortexing and centrifugation, the supernatants were incubated with the indicated antibodies and protein A/G magnetic beads (HY-K0202, MCE) overnight at 4°C. The bead-bound complexes were washed four times and eluted by 1x SDS-PAGE loading buffer. The immunoprecipitants were subjected to immunoblotting analysis.

**Click-iT™ *O*-GlcNAc Enzymatic Labeling Assay**

Cells were lysed and 100 μg of total protein was labeled with GalNAz and biotin alkyne using the Click-iT^TM^ *O*-GlcNAc Enzymatic Labeling System (C33368, Invitrogen) and the Click-iT^TM^ Glycoprotein detection kit (C33372, Invitrogen). After labeling, proteins were eluted in 1x SDS-PAGE loading buffer and analyzed using western blotting.

**sWGA Pull-down Assay**

Cells or liver tissues were lysed in lysis buffer (125 mM NaCl, 5 mM EDTA, 0.1% NP-40, 50 mM Tris, pH 7.4). After centrifugation, the supernatants were denatured and digested with PNGase F (QPF-003-A-PKG, Adamas life, China) to remove N-linked glycoproteins. Precleared lysates were incubated with sWGA-conjugated agarose beads (AL-1023S, Vector Laboratories, Burlingame, USA) overnight at 4°C. To confirm binding specificity, parallel samples were co‑incubated with 0.5 M GlcNAc. The beads-bound complexes were washed and eluted for immunoblotting.

**FTO Protein Stability Assay**

Cells were treated with 300 μM cycloheximide (CHX, AC466, Glenview, Beijing, China) to inhibit protein synthesis and harvested at 0, 12, 24 and 36 h post-treatment. The cells were lysed and centrifuged at 14000 g at 4°C for 20 min. The supernatant protein was quantified using a BCA Protein Assay Kit. Equal amounts of proteins were subjected to immunoblotting as described before. The blotting signals were quantified by using ImageJ (NIH, USA) and normalized against the 0 h time point.

**mRNA Stability Assay**

Cells were treated with 5 μg/mL actinomycin D (S8964, Selleck, TX, USA) and harvested at 0, 4, 8 and 12 h. Total RNAs were extracted, reverse-transcribed, and analyzed by RT-qPCR. Within each group, the mRNA abundance at the 0 h time point was set to 100%. The mRNA half‑life was calculated using the equation (*t*_1/2_ = ln 2/*k*_decay_).

**Immunofluorescence and Nile Red Staining**

For immunofluorescence, cells were fixed with 4% paraformaldehyde (PFA) for 30 min, permeabilized with 0.1% Triton X-100 for 20 min, and incubated with indicated primary antibody overnight at 4°C, followed by fluorescence-labeled secondary antibody for 1 h at room temperature. Nuclei were stained with 1 μg/mL DAPI (0236276001, Roche, Mannheim, Germany).

For Nile Red staining, cells were fixed with 4% PFA and incubated with 1 μM Nile Red (HY-D0718, MCE) for 20 min at room temperature, followed by 1 μg/mL DAPI staining. All images were acquired with a Leica confocal microscope (Leica TCS SP8, Leica Microsystems, Wetzlar, Germany).

**Histology and Immunohistochemistry**

Liver tissues were fixed in 4% PFA and subsequently sent to Lilai Biotechnology (Chengdu, China) for paraffin embedding, serial sectioning, and hematoxylin and eosin (H&E) staining. Sirius Red staining (G1473, Solarbio, Beijing, China) was performed to assess collagen deposition and fibrosis, while Oil Red O (ORO) staining (G1260, Solarbio) was used to evaluate neutral lipid accumulation, both following the manufacturer's protocols.

For immunohistochemical staining, serial sections were blocked with normal goat serum (ZLI-9022, ZSGB-BIO, Beijing, China) for 1 h, followed by overnight incubation at 4°C with primary antibodies against FTO, *O*-GlcNAc, α-SMA, F4/80, p-AKT, HRAS, FTO Ser195-specific *O*-GlcNAc. Sections were then incubated with secondary antibodies (PV-9005, ZSGB-BIO, Beijing, China) for 30 min. All slides were scanned using the MoticEasyScanner (Motic, Hong Kong, China) and visualized with CaseViewer software (3DHISTECH, Budapest, Hungary).

For pathological evaluation, human and murine liver specimens were independently scored by two experienced pathologists according to the NAFLD activity score (NAS), which comprises steatosis (0–3), lobular inflammation (0–3), and hepatocellular ballooning (0–2), yielding a total score ranging from 0 to 8.^[4]^ ORO, Sirius Red, α-SMA and F4/80-positive areas were quantified using ImageJ software (version 1.6.0; NIH, MD, USA).Immunohistochemical staining was semi-quantitatively analyzed using the immunoreactive scoring system.^[5]^

**RNA Immunoprecipitation**

RNA immunoprecipitation (RIP) was performed using Magna RIP™ RNA-Binding Protein Immunoprecipitation Kit (17-700, Millipore). Following the manufacturer’s instructions, cells were washed, irradiated at 254 nm with 500 mJ/cm^2^ in a UV crosslinker for 10 minutes, and lysed in RIP lysis buffer. The supernatants were immunoprecipitated with protein A/G magnetic beads conjugated to anti-FTO or anti-YTHDF2 antibody for 4–6 h. RNA was extracted from the immunoprecipitates using TRIzol and analyzed by RT-qPCR with specific primers (Table S1). Relative RNA enrichment was normalized to input controls. Fold enrichment was normalized to the IgG control.

**RNA m6A Dot Blotting**

Total RNA was isolated, and mRNA was purified using the mRNA Purification Kit (NR105, TIANGEN, Beijing, China). Purified mRNA was denatured at 95°C for 5 min and immediately cooled on ice. Equal amounts of mRNA samples (100, 200, and 400 ng) were spotted onto Hybond-N+ membranes (11417240001, Roche) and UV-crosslinked (125 mJ/cm^2^). The membrane was blocked with 5% skim milk, incubated with the anti-m6A antibody overnight at 4°C, and subsequently detected by enhanced chemiluminescence (ECL). Methylene blue (G1301, Solarbio) staining was used to verify mRNA loading uniformity.

**RNA Pull-down Assay**

Biotin-labeled RNA oligonucleotides containing A or m6A were synthesized by Tsingke Biotechnology (Tsingke, Beijing, China). Briefly, Cell lysates were incubated with 1 nmol biotin-labeled oligos and 30 μL Streptavidin Agarose beads overnight at 4°C. After washing, the bead-bound complexes were eluted and analyzed by western blotting. The oligos sequences are provided in Table S1.

***In vitro* FTO Demethylation Activity Assay**

As previously described,^[6,7]^ the FTO demethylation activity assay was performed in 20 μL reaction buffer containing 5 μM A or m6A oligos, 500 ng Flag-tagged FTO protein (purified from MIHA cells), 1.6 U/μL RNasin (HY-K1033, MCE). After incubation at room temperature for 3 h, reactions were heat-denatured at 95°C for 5 min. Next, 2 μL of the reaction product was used for m6A dot blot. Sequences of all RNA oligos are provided in Table S1.

**RNA-seq and MeRIP-seq**

FFA-treated (OA:PA = 2:1) sg-Control and sg-FTO MIHA cells were sent to Cloud-seq Biotech (Shanghai, China) for RNA-seq and MeRIP-seq. Briefly, total RNA was extracted, ribosomal RNA was depleted, and libraries were constructed for sequencing on Illumina HiSeq platform (150 bp paired-ended mode). MeRIP-seq utilized an m6A antibody, and data were analyzed with Model-based Analysis of ChIP-Seq (MACS) software for m6A peaks and Hypergeometric Optimization of Motif EnRichment (HOMER) for consensus motifs enrichment analysis.

**Sex as A Biological Variable**

For animal studies, only male mice were employed to preclude confounding effects of estrogen. Female mice were excluded due to their greater interindividual variability than males in metabolic parameters, which stems from the critical role of estrogen and estrogen receptor signaling in modulating hepatic lipid metabolism—a core pathological feature of MASH. Human samples from both males and females were included in this study. Sex was not considered as a biological variable for human samples.

**Supplementary Figures**

**Figure S1. GAN diet induced hepatic steatohepatitis in mice.**

**(A-L)** Male C57BL/6 mice (n = 5) were fed a GAN diet for 24 weeks. Gross morphology of mice (A) and livers (B). Mice body weights were measured biweekly (C). The liver weights (D), the ratio of liver weight to body weight (E), GTT (F), ITT (G), serum AST (H), ALT (I), TC (J), TG (K), FFA (L) concentrations. **(M)** Relative mRNA levels of genes related to *de novo* lipogenesis, fatty acid transport, inflammation and fibrosis were quantified by RT-qPCR (n = 5, performed in triplicate). For (C-M), statistical data are presented as mean ± SD and performed two-tailed Student’s t-test. **p* < 0.05, ***p* < 0.01 and ****p* < 0.001.

**Figure S2. *Pten* liver-specific knockout mice developed hepatic steatohepatitis.**

**(A-L)** *Pten*^flox/flox^; Albumin-Cre mice (*Pten* L-KO mice) (n = 5) were fed for 40 weeks. The gross morphology of mice (A) and livers (B). Mice body weights were measured monthly (C). The liver weights (D), the ratio of liver weight to body weight (E), GTT (F), ITT (G), serum AST (H), ALT (I), TC (J), TG (K), FFA (L) concentrations. **(M)** Relative mRNA levels of genes related to *de novo* lipogenesis, fatty acid transport, inflammation and fibrosis were identified by RT-qPCR (n = 5, performed in triplicate). For (C-M), statistical data are presented as mean ± SD and performed two-tailed Student’s t-test. **p* < 0.05, ***p* < 0.01, ****p* < 0.001 and ns, not significant.

**Figure S3. Hepatic HBP pathway and relative metabolites were upregulated in MASH mice models.**

**(A)** Orthogonal partial least-square discriminant analysis (OPLS-DA) of untargeted metabolomics showing distinct clustering between Chow diet and GAN diet-fed mice livers. **(B)** The pie chart of hepatic identified metabolites classification. **(C, D)** MIHA (C) and PMH (D) were treated with increasing concentration FFA (20, 50, 100, 200, 400 μM) or BSA for 24 h. Cellular metabolites were extracted, and UDP-GlcNAc abundance was detected by LC-MS/MS. **(E)** Immunoblot analysis of *Pten* L-KO mice liver tissues. **(F, G)** Hepatic *Oga* and *Ogt* mRNA levels in GAN diet-fed mice (F) and *Pten* L-KO mice (G). For (C, D, F, G), statistical data are presented as mean ± SD. For (C, D), statistical analyses were performed using one-way ANOVA followed by Tukey’s test. For (F, G), statistical analyses were performed using two-tailed Student’s t-test. **p* < 0.05, ***p* < 0.01 and ns, not significant.

**Figure S4. Biological functions of *O*-GlcNAcylation 4D-Label free quantitative proteomics.**

**(A)** Subcellular localization of the identified *O*-GlcNAcylated proteins. **(B, C)** Gene Ontology analysis (B) and KEGG enrichment analysis (C) of *O*-GlcNAc-modified proteins. **(D)** Hepatic FTO protein levels and FTO *O*-GlcNAcylation intensity in *Pten* L-KO mice: immunoblot (top), IHC (bottom) and sWGA pull-down (top panels, with quantification at left), scale bar = 50 μm. For D, statistical data are presented as mean ± SD and performed two-tailed Student’s t-test. ***p* < 0.01.

**Figure S5. Ser195 is the major *O*-GlcNAcylation site of FTO.**

**(A)** The Co-IP of endogenous OGT and FTO in PMH cells. **(B)** Subcellular colocalization of endogenous OGT and FTO in PMH cells via immunofluorescence, scale bar = 20 μm. **(C, D)** MIHA cells were transfected with FTO-Flag plasmids and treated with DMSO, 25 μM TMG (C) or 40 μM OSMI-1 (D) for 12 h. FTO *O*-GlcNAcylation intensity was assessed by IP. **(E-I)** sWGA pull-down to detect endogenous (E, F, I) and exogenous (G, H) FTO *O*- GlcNAcylation intensity. **(J, K)** Click-iT™ *O*-GlcNAc Enzymatic Labeling assay was used to detect FTO *O*-GlcNAcylation intensity.

**Figure S6. *O*-GlcNAcylation of FTO suppresses ubiquitin-mediated proteasomal degradation.**

**(A-C)** Half-life and quantitative analysis of FTO in MIHA cells (A) and PMH cells (B) treated with DMSO, 25 μM TMG, 40 μM OSMI-1 or infected with shCon or shOGT lentivirus (C). Cells were treated with 300 μM cycloheximide (CHX) for the indicated times (n = 3, performed in triplicate). For each group, the blotting signals were quantified by using ImageJ and normalized against the 0 h time point. **(D)** MIHA cells were transfected with HA-Ub and FTO-WT plasmids and treated with DMSO, 25 μM TMG, or 40 μM OSMI-1 for 12 h. Before harvested, cells were treated with 15 μM MG132 for 6 h and were divided into two parts for sWGA pull-down and FTO ubiquitination assay. **(E)** Screening of ubiquitin ligases and deubiquitinases affecting FTO stability in MIHA cells. **(F, G)** MIHA cells were transfected with vector plasmids or increasing concentration (0.5, 1, 2, 4 μg) USP20-HA (F) or FBXW7-HA (G) plasmids and then harvested cells for immunoblot. For (A, B, C), statistical data are presented as mean ± SD. For (A, B), statistical analysis was performed using one-way ANOVA followed by Tukey’s test. For (C), statistical analysis was performed using two-tailed Student’s t-test. **p* < 0.05, ***p* < 0.01 and ****p* < 0.001.

**Figure S7. *O*-GlcNAcylation enhanced FTO enzymatic activity by stabilizing its protein abundance.**

**(A)** The workflow of FTO enzymatic activity assay. **(B)** MIHA cells were infected with sg-FTO viruses to knock down endogenous FTO protein and transfected with FTO-Flag plasmids to overexpress exogenous FTO protein, followed by culture with DMSO, 25 μM TMG or 40 μM OSMI-1 for 12 h. m6A dot blot was performed to test FTO enzymatic activity. Immunopurified FTO, purified by IP assay, was performed for *in vitro* FTO demethylation activity assay. **(C)** RNA pull-down assay was performed for FTO binding activity. **(D)** MIHA cells were infected with sg-FTO viruses to knock down endogenous FTO. Plasmid transfection was then adjusted to achieve comparable Flag-tagged FTO expression levels (verified by immunoblot). Performed the m6A dot blot to test FTO enzymatic activity. **(E, F)** *In vitro* FTO demethylation activity assay (E) and RNA pull-down (F) were performed to detect the enzyme catalytic efficiency of Flag-tagged FTO protein.

**Figure S8. FFA enhances FTO protein abundance through *O*-GlcNAcylation.**

**(A-F)** MIHA or PMH cells were cultured with increasing concentration free fatty acid (FFA; OA:PA = 2: 1) or vehicle (BSA) for 24 h and harvested for immunoblotting (A, D) and RNA extraction (B, E). Lipid droplet accumulation (Oil Red O; scale bar = 50 μm) and FTO *O*-GlcNAcylation (sWGA pull-down) in FFA-treated MIHA (C) and PMH (F) cells (200 μM, 24 h). For (B, E), statistical data are presented as mean ± SD and performed one-way ANOVA followed by Tukey’s test.

**Figure S9. *HRAS* mRNA is a downstream target of FTO.**

**(A)** m6A consensus sequence motifs in sg-Control and sg-FTO cells were identified by Discriminative Regular Expression Motif Elicitation (DREME). **(B)** GO enrichment of overlapped genes. **(C)** Multiple GSEA pathways analysis of RNA-Seq. **(D)** Relative mRNA levels of MAPK signaling pathway enriched genes in PMH cells identified by RT-qPCR (n = 3, performed in triplicate). **(E)** MeRIP-qPCR to detect m6A intensity of *Hras* mRNA in PMH cells. **(F)** RIP-qPCR using anti-FTO antibody in PMH cells. **(G)** RNA pull-down in PMH cells. Statistical data are presented as mean ± SD. For (F), statistical analysis was performed using two-tailed Student’s t-test. For (D, E), statistical analysis was performed using one-way ANOVA followed by Tukey’s test. * *p* < 0.05, ***p* < 0.01 and ****p* < 0.001.

**Figure S10. FTO promotes intracellular lipid accumulation via HRAS/PI3K/AKT axis.**

**(A)** Immunoblot analysis detecting the enrichment of m6A readers (YTHDF1/2/3, IGF2BP1/2/3) in RNA pull-down assay using A or m6A HRAS probe in PMH cells. **(B)** RIP-qPCR using anti-YTHDF2 antibody in PMH cells. (C) Immunoblotting analysis of protein levels in PMH cells. **(D-F)** Sg-Control MIHA cells infected with shControl or shHRAS viruses. Sg-FTO MIHA cells transfected with HRAS-HA or vector plasmids. Intracellular TC (D) and TG (E) levels were measured and normalized to the control group. Lipid droplets were visualized by Oil Red O (top, scale bar = 50 μm) and Nile Red (bottom, scale bar = 20 μm) staining (F). **(G, H)** After infected with sg-Control or sg-FTO viruses, MIHA cells were transfected with Flag-tagged plasmids and cultured with DMSO or 10 μM MK2206 (AKT inhibitor) for 24 h, then measured the intracellular TC (G) and TG (H) levels. Statistical data are presented as mean ± SD. For (B), statistical analysis was performed using two-tailed Student’s t-test. For (D, E, G, H), statistical analysis was performed using one-way ANOVA followed by Tukey’s test. **p* < 0.05, ***p* < 0.01 and ****p* < 0.001.

**Figure S11. Targeting FTO *O*-GlcNAcylation and AKT alleviates MASH *in vivo*.**

**(A-J)** Gross morphology of mice (A) and livers (B). Mice body weight curves (C). Liver TNF-α (D) and IL-6 (E) levels. Serum TC (F), TG (G), and FFA (H) concentrations. **(I, J)** The semi-quantitative analyses of immunohistochemistry data of mouse liver tissues. For (C-J), statistical data are presented as mean ± SD and performed using one-way ANOVA followed by Tukey’s test. **p* < 0.05, ***p* < 0.01 and ****p* < 0.001.

**Figure S12. AKT inhibitor MK2206 blocks MASH progression driven by FTO *O*-GlcNAcylation.**

**(A)** Relative mRNA levels of genes related to *de novo* lipogenesis, fatty acid transport, inflammation and fibrosis were quantified by RT-qPCR (n = 5, performed in triplicate). **(B)** Immunoblot analysis of protein expression in mouse liver tissues. For (A), statistical data are presented as mean ± SD and performed using one-way ANOVA followed by Tukey’s test. **p* < 0.05, ***p* < 0.01 and ****p* < 0.001.

**Figure S13. Cellular uptake and *in vivo* biodistribution of CPPs.**

**(A, B)** Cytotoxicity of CPPs was assessed in MIHA (A) and PMH (B) cells. **(C)** Schematic diagram illustrating the construction of CY5-labeled CPPs. **(D, E)** Time-dependent cellular uptake of 20 μM CY5-CPPs in MIHA (D) and PMH (E) cells at 0, 3, 6, and 12 h. Scale bar = 100 μm. **(F)** *In vivo* fluorescence imaging and *ex vivo* organ imaging of C57BL/6J mice at 12 h after intraperitoneal injection of CY5-CPPs (5 mg/kg).

**Figure S14. CPPs-mediated inhibition of FTO Ser195 *O*-GlcNAcylation attenuates intracellular lipid accumulation *in vitro*.**

**(A-D)** PMH cells pretreated with 200 μM FFA or BSA control, followed by CPPs-S195-3# or CPPs-Con (20 μM). Immunoblotting(A). Lipid droplets were visualized by Oil Red O (top, scale bar = 50 μm) and Nile Red (bottom, scale bar = 20 μm) staining (B). Total cholesterol (C) and triglyceride (D) levels were measured and normalized to the BSA control group. For (C, D), statistical data are presented as mean ± SD and performed using one-way ANOVA followed by Tukey’s test. ****p* < 0.001.

**Figure S15. CPPs-mediated inhibition of FTO Ser195 *O*-GlcNAcylation alleviates MASH progression *in vivo*.**

**(A-N)** Gross morphology of mice (A) and livers (B). Mice body weights were measured biweekly (C). GTT (D), ITT (E). The liver weights (F) and the ratio of liver weight to body weight (G). Liver TNF-α (H) and IL-6 (I) levels. Serum AST (J), ALT (K), TC (L), TG (M), and FFA (N) concentrations. **(O-R)** The quantitative and semi-quantitative analyses of immunohistochemistry data of mouse liver tissues. For (C-R), statistical data are presented as mean ± SD and performed using one-way ANOVA followed by Tukey’s test. **p* < 0.05, ***p* < 0.01, ****p* < 0.001 and ns, not significant.

**Figure S16. *In vivo* safety evaluation of CPPs-treated mice.**

**(A)** Relative mRNA levels of genes related to *de novo* lipogenesis, fatty acid transport, inflammation and fibrosis were quantified by RT-qPCR (n = 5, performed in triplicate). **(B)** Immunoblot analysis of CPPs-treated mice liver tissues. **(C-F)** Serum levels of renal function markers (urea (UREA, C) and creatinine (CREA, D)) and cardiac function markers (creatine kinase (CK, E) and α-hydroxybutyrate dehydrogenase (α-HBDH, F)) in CPPs-treated mice. **(G)** Representative hematoxylin and eosin (H&E) staining of heart, spleen, lung and kidney sections from CPPs-treated mice for histological safety evaluation. Scale bar = 50 μm. For (A, C-F), statistical data are presented as mean ± SD and performed using one-way ANOVA followed by Tukey’s test. **p* < 0.05, ***p* < 0.01, ****p* < 0.001 and ns, not significant.

**Figure S17. The FTO/HRAS/PI3K/AKT signaling axis is significantly upregulated in GEO database.**

**(A)** *FTO* mRNA expression levels in GEO datasets. **(B)** GSEA analysis in GSE130970. **(C, D)** Correlation analysis of FTO and HRAS (C) or p-AKT (D) protein expression in clinical MASH samples. Statistical data are presented as mean ± SD. For A (left), statistical analysis was performed using two-tailed Student’s t-test. For A (right), statistical analysis was performed using one-way ANOVA followed by Tukey’s test. ns, not significant.

**Supplementary tables**

**Table S1 Primer sequences used in this study.**

| qRT-PCR Primers | | | |
| --- | --- | --- | --- |
| Species | Gene | Forward (5’-3’) | Reverse (5’-3’) |
| mouse | β-actin | CGTTCAATACCCCAGCCATG | GACCCCGTCACCAGAGTCC |
| mouse | Acly | CTCATTGAACCCTTCGTCCC | CCTCGGTATTCAGCTTTTCGT |
| mouse | Fasn | CTGCCTCCGTGGACCTTATC | GCACAGACACCTTCCCGTCA |
| mouse | Acaca | ATTGCCTATGAACTCAACAGCG | TGACAAGGTGGCGTGAAGG |
| mouse | Scd1 | TTCCTTATCATTGCCAACACCA | TTCCTTATCATTGCCAACACCA |
| mouse | Mlxipl | GCTGCGGGATGAAATAGAGG | TCAAATAAAGGTCGGATGAGGA |
| mouse | Srebf1 | ACAGACAAACTGCCCATCCA | CCTCCTCCACTGCCACAAG |
| mouse | Fabp1 | CCAGGAGAACTTTGAGCCATT | GTCACCTTCCAGCTTGACGA |
| mouse | Cd36 | ACTGTGGGCTCATTGCTGG | TGATTTTGCTGCTGTTCTTTGC |
| mouse | Slc27a1 | GCTCCTGCGGCTTCAACA | GCGCTATCGCCCTTTCG |
| mouse | Il-6 | GTTGTGCAATGGCAATTCTGA | AAGGACTCTGGCTTTGTCTTTCT |
| mouse | Il-10 | GGTTGCCAAGCCTTATCGG | ATTTTCACAGGGGAGAAATCG |
| mouse | Il-1b | AAGCCTCGTGCTGTCGGA | CCATCTTCTTCTTTGGGTATTGC |
| mouse | Tnf-α | CCTGCCCCAAGGACACC | AGAGCAATGACTCCAAAGTAGACC |
| mouse | Ccl2 | TGTGCTGACCCCAAGAAGG | GGTGGTTGTGGAAAAGGTAGTG |
| mouse | Acta2 | CCCTGAAGAGCATCCGACA | CATCTCCAGAGTCCAGCACAA |
| mouse | Col1a1 | ACCCTGCCCGCACATG | CCCTCGCTTCCGTACTCG |
| mouse | Timp1 | GTCCCAGAACCGCAGTGAA | TGGCAGGCAAGCAAAGTG |
| mouse | Col3a1 | GCCCACAGCCTTCTACACCT | TCCCGGATAGCCACCCA |
| mouse | Oga | CTTTTGTGCAGTGGTTAGGGT | AAACTTTGGAGGTTGGAGTCAG |
| mouse | Ogt | ACTTCGGGAATCACCCTACTT | CATCTTGGTGGATGCGGTC |
| mouse | Fto | CAATGCCACCCACCAGC | GCCTCAGCCACTCAAACTCC |
| mouse | Hras | GTATTTGCCATCAACAACACCA | CTGCCGAATCTCACGGACTA |
| mouse | Egfr | GCGTTGGAGGAAAAGAAAGTC | GGTGTTGAGGGCAATGAGG |
| mouse | Cacna1h | AGAGCCGTTGGCGTAAGAA | GTAATGGTGGTGATGGTGGTG |
| mouse | Map3k13 | CGGCATCCCAGACTCAATG | CAGCCCCAGGGTCCAACT |
| human | β-actin | AGGCCAACCGCGAGAAGATGACC | GAAGTCCAGGGCGACGTAGCAC |
| human | OGA | AGCAGCCTTGAGTGGTGAGC | TGGGGATTTTGATTCAGCTATG |
| human | OGT | AGAGGCAGTTCGCTTGTATCG | AGTAGGCATCAGCAAAGGTAGG |
| human | FTO | CCCCGTGGAACAAAGGAA | AGGTCAAACGGCAGAGGC |
| human | EGFR | GATGGCATTGGAATCAATTTTAC | CCCTGAATGACAAGGTAGCG |
| human | HRAS | GGAATCTCGGCAGGCTCA | GGATCTCACGCACCAACGT |
| human | CACNA1H | GAGCACTATAACCAACCCAAGTC | ACGGGCAATGCGAAGC |
| human | MAP3K13 | AGATGTCATTTGCTGGCACG | AACTGGAAGGTGGAGGCTGT |
| human | SCD1 | CCCCTCTACTTGGAAGACGAC | TCATTCTGGAATGCCATTGTG |
| human | ACACA | ACCCGAACAGTAGAACTAAGTATCC | CATCCACAATGTAAGCACCAAA |
| human | FASN | GCTGGACTACTTTGTGGTCTTCTC | ATCGTGTCGTTGGTGCTCAT |
| human | ChREBP | CGCCTGAACAACGCCATC | CCGCATCACCACCTCGA |
| human | CD36 | CATCGCTGGGGCTGTCA | GTTGCTGCTGTTCATCATCACT |
| human | PPARA | CCCTCCTCGGTGACTTATCC | ACTGGCATTTGTTTCTGTTCTTT |
| human | CCL2 | TCATAGCAGCCACCTTCATTC | AAGTCTTCGGAGTTTGGGTTT |
| human | IL-6 | TGTGAAAGCAGCAAAGAGGC | TGGGTCAGGGGTGGTTATT |
| Mice Genotyping PCR Primers | | | |
| Species | Name | Forward (5’-3’) | Reverse (5’-3’) |
| mouse | *Pten*^f/f^ | ATCCCCACCAATGAACAAAC | CTCCTCTACTCCATTCTTCCC |
| mouse | Cre typing | CACCCTGTTACGTATAGCCG | GAGTCATCCTTAGCGCCGTA |
| Molecular Cloning Primers | | | |
| Species | Name | Forward (5’-3’) | Reverse (5’-3’) |
| human | pSEB-3Flag-FTO | TCCAAGCTTACCATGGGCAAGCGCACCCCGACTG | CGCGTCGACGGGTTTTGCTTCCAGAAGCTG |
| human | pSEB-3Flag-FTO-ΔC | TCCAAGCTTACCATGGGCAAGCGCACCCCGACTG | CGCGTCGACTGAGCACTCTGCCACTCGG |
| human | pSEB-3Flag-FTO-ΔN | TCCAAGCTTACCATGGGCTCAACAGGAACCTTGGATTAT | CGCGTCGACGGGTTTTGCTTCCAGAAGCTG |
| human | pSEB-3Flag-YTHDF2 | ATAGCGGCCGCACCATGGGCATGTCGGCCAGCAGCCTCTTG | CGCGGATCCTTTCCCACGACCTTGACGTTC |
| human | pAdTrack-3Flag-TO4-FTO | TGGGGTACCATGGGCAAGCGCACCCCGACTG | CCCAAGCTTTTAAATCTTATCGTCGTCATCCT |
| human | pAdTrack-3Flag-TO4-FTO-S195A | AGTGGACATTAAGGCCAGAGCAGCATACAA | GTATGCTGCTCTGGCCTTAATGTCCACTTC |
| human | pAdTrack-3Flag-TO4-FTO- H231A/ D233A/ R316Q/ R322Q | H231A/ D233A: GAAAATGGCAGTGAGCTGGGCTCATGCTGAAAATCTGGTGGACAGG  R316Q/ R322Q: GCCGGTTCACAACCTCAGTTTAGTTCCACCCACCAAGTGGCAGAGTGCTC | H231A/ D233A: CCTGTCCACCAGATTTTCAGCATGAGCCCAGCTCACTGCCATTTTC  R316Q/ R322Q: GAGCACTCTGCCACTTGGTGGGTGGAACTAAACTGAGGTTGTGAACCGGC |
| human | pBudCE-3HA-HRAS | TATGGTACCATGGGCACGGAATATAAGCTGGTG | GGAAGATCTCTGGAGAGCACACACTTGCAG |
| human | pBudCE-3HA-OGT | ATAGCGGCCGCACCATGGCGTCTTCCGTGGGC | CGCGGATCCTGCTGACTCAGTGACTTCAACAGG |
| human | pBudCE-3HA-SPOP | TGGGGTACCATGGGCTCAAGGGTTCCAAGTCCTC | CCGCTCGAGTCGGATTGCTTCAGGCGTTTG |
| human | pBudCE-3HA-CDH1 | TGGGGTACCATGGACCAGGACTATGAGCG | GGAGATATCCCGGATCCTGGTGAAGAG |
| human | pBudCE-3HA-STRAP | TGGGGTACCATGGCAATGAGACAGACG | GGAAGATCTCTGGCCTTAACATCAGGAGC |
| human | pBudCE-3HA-MIB1 | ATAGCGGCCGCACCATGAGTAACTCCCGGAATAA | CCGCTCGAGTCATACAAAAGAATCCTTCGTTC |
| human | pBudCE-3HA-USP18 | TGG GGTACC ATGAGCAAGGCGTTTGG | CCG CTCGAG TC GCACTCCATCTTCATGTAAACC |
| human | pBudCE-3HA-FBXW7 | TGGGGTACCATGGGCAATCAGGAACTGCTCTCTGTG | TGAAGATCTGACTTCATGTCCACATCAAAGTCC |
| Sequences of shRNAs, sgRNAs and siRNAs | | | |
| Species | Name | Forward (5’-3’) | Reverse (5’-3’) |
| human | shOGT#1 | TGCATGTTATTTGAAAGCAATTCAAGAGATTGCTTTCAAATAACATGCTTTTTTC | TCGAGAAAAAAGCATGTTATTTGAAAGCAATCTCTTGAATTGCTTTCAAATAACATGCA |
| human | shOGT#2 | TGCCCTAAGTTTGAGTCCAATTCAAGAGATTGGACTCAAACTTAGGGCTTTTTTC | TCGAGAAAAAAGCCCTAAGTTTGAGTCCAATCTCTTGAATTGGACTCAAACTTAGGGCA |
| human | shYTHDF2#1 | TGCAACGGTTGCATCTGCATATTTCAAGAGAATATGCAGATGCAACCGTTGCTTTTTTC | TCGAGAAAAAAGCAACGGTTGCATCTGCATATTCTCTTGAAATATGCAGATGCAACCGTTGCA |
| human | shYTHDF2#2 | TGCAGCAACGGTTGCATCTGCATTCAAGAGATGCAGATGCAACCGTTGCTGCTTTTTTC | TCGAGAAAAAAGCAGCAACGGTTGCATCTGCATCTCTTGAATGCAGATGCAACCGTTGCTGCA |
| human | shHRAS#1 | TGCTGCACGCACTGTGGAATCTTTCAAGAGAAGATTCCACAGTGCGTGCAGCTTTTTTC | TCGAGAAAAAAGCTGCACGCACTGTGGAATCTTCTCTTGAAAGATTCCACAGTGCGTGCAGCA |
| human | shHRAS#2 | TGCAGATCAAACGGGTGAAGGATTCAAGAGATCCTTCACCCGTTTGATCTGCTTTTTTC | TCGAGAAAAAAGCAGATCAAACGGGTGAAGGATCTCTTGAATCCTTCACCCGTTTGATCTGCA |
| human | shHRAS#3 | TGGAGTGGAGGATGCCTTCTACTTCAAGAGAGTAGAAGGCATCCTCCACTCCTTTTTTC | TCGAGAAAAAAGGAGTGGAGGATGCCTTCTACTCTCTTGAAGTAGAAGGCATCCTCCACTCCA |
| human | sgFTO#1 | CACCGTTCTGGCGTGCAGTGAGCG | AAACCGCTCACTGCACGCCAGAAC |
| human | sgFTO#2 | CACCGGCGTGCAGTGAGCGAGGCA | AAACTGCCTCGCTCACTGCACGCC |
| human | sgFTO#3 | CACCGATTTCATTCCTTTGTTCCA | AAACTGGAACAAAGGAATGAAATC |
| mouse | siFto#1 | CCAGGGAGACTGCTATTTCAT |  |
| mouse | siFto#2 | GTCTCGTTGAAATCCTTTGAT |  |
| mouse | siFto#3 | TAGTCTGACTTGGTGTTAAAT |  |
| Oligos sequences | | | |
| Species | Name | Forward (5’-3’) |  |
| human | A-oligos | AUUGUCACAGCAGCAG |  |
| human | m6A-oligos | AUUGUCA(m6A)CAGCAGCAG |  |
| human | Biotin-A-oligos | Biotin-CGUCUCGGACUCGGACUGCU |  |
| human | Biotin-m6A-oligos | Biotin-CGUCUCGGA(m6A)CUCGGA(m6A)CUGCU |  |
| human | A-HRAS-oligos | CCGUUCACAGGCGCGACUGCCC |  |
| human | m6A-HRAS-oligos | CCGUUCACAGGCGCGA(m6A)CUGCCC |  |
| human | Biotin-A-HRAS-oligos | Biotin-CCGUUCACAGGCGCGACUGCCC |  |
| human | Biotin-m6A-HRAS-oligos | Biotin-CCGUUCACAGGCGCGA(m6A)CUGCCC |  |

**Table S2 Antibodies used in this study.**

| Antibodies Name | Supplier | Cat no. | Clone no. |
| --- | --- | --- | --- |
| Goat Anti-Rabbit IgG | Bio-Rad | 1706515 | Polyclonal |
| Goat Anti-Mouse IgG | Bio-Rad | 1706516 | Polyclonal |
| Mouse IgG Control | Invitrogen | 10400C | Polyclonal |
| Rabbit IgG Control | Proteintech | 30000-0-AP | Polyclonal |
| Rabbit anti-OGT | Abcam | ab177941 | Clone  EPR12713 |
| Rabbit anti-OGA | Abcam | ab124807 | Clone  EPR7154(B) |
| Mouse anti-O-GlcNAc | Abcam | ab2739 | Clone  RL2 |
| Mouse anti-β-actin | ZSGB-BIO | TA-09 | Clone  OTI1 |
| Rabbit anti-FTO | Proteintech | 27226-1-AP | Polyclonal |
| Rabbit anti-FTO | Abcam | ab126605 | Clone  EPR6894 |
| Mouse anti-HA | Invitrogen | 26183 | Clone  2-2.2.14 |
| Mouse anti-Flag | Sigma | F3165 | Clone  M2 |
| Mouse anti-m6A | Abcam | ab208577 | Clone  17-3-4-1 |
| Rabbit anti-YTHDF1 | Huabio | HA721302 | Clone  PSH0-23 |
| Rabbit anti-YTHDF2 | Abcam | ab220163 | Clone  EPR20318 |
| Mouse anti-YTHDF3 | Santa Cruz Biotechnology | sc-377119 | Clone  F-2 |
| Mouse anti-IGF2BP1 | Santa Cruz Biotechnology | sc-166344 | Clone  D-9 |
| Rabbit anti-IGF2BP2 | Huabio | HA720003 | Clone  JE56-58 |
| Rabbit anti-IGF2BP3 | Proteintech | 81805-1-RR | Clone  1L11 |
| Rabbit anti-F4/80 | Huabio | HA721520 | Clone  PSH0-85 |
| Rabbit anti-α-SMA | Huabio | ET1607-53 | Clone  SY02-64 |
| Rabbit anti-HRAS | Proteintech | 18295-1-AP | Polyclonal |
| Rabbit anti-Raf1 | Zenbio | 347271 | Polyclonal |
| Rabbit anti- phospho-Raf1 (Ser259) | Zenbio | R25537 | Clone  R07-4J4 |
| Rabbit anti-MEK1/2 | Zenbio | 380797 | Polyclonal |
| Rabbit anti-Phospho-MEK1/2 (Ser217, 221) | SinoBiological | 110470-R0008 | Clone  0008 |
| Rabbit anti-ERK1/2 | Abcam | ab184699 | Clone  EPR17526 |
| Rabbit anti-phospho-ERK1/2 (Thr202, Tyr204) | SinoBiological | 110441-R0072 | Clone  0072 |
| Rabbit anti-AKT | Cell signaling technology | 4691 | Clone  C67E7 |
| Rabbit anti-Phospho-AKT (Ser473) | Cell signaling technology | 4060 | Clone  DE9 |
| Rabbit anti-S195-O-FTO | MabStar | Custom Antibody | Polyclonal |

**Table S3 Clinical information for patients with MASH**

|  | MASH (n = 15) |
| --- | --- |
| Sex, n (%) |  |
| Male | 10 (66.7) |
| Female | 5 (33.3) |
| Age (years), median (range) | 36 (25.1-47.1) |
| NAS score, median (range) | 6.1 (5.4-6.8) |
| Steatosis score, n (%) |  |
| 1 | 0 (0) |
| 2 | 6 (40) |
| 3 | 9 (60) |
| Hepatocyte ballooning score, n (%) |  |
| 1 | 8 (53.3) |
| 2 | 7 (46.7) |
| Lobular inflammation score, n (%) |  |
| 1 | 2 (13.3) |
| 2 | 11 (73.3) |
| 3 | 2 (13.4) |

Data are presented as the percentage or median (interquartile range).

**Supplementary references**

[1]	Y. Horie, A. Suzuki, E. Kataoka, T. Sasaki, K. Hamada, J. Sasaki, K. Mizuno, G. Hasegawa, H. Kishimoto, M. Iizuka, M. Naito, K. Enomoto, S. Watanabe, T. W. Mak, T. Nakano, *Journal of Clinical Investigation* **2004**, *113*, 1774.

[2]	M. I. Hernández-Alvarez, D. Sebastián, S. Vives, S. Ivanova, P. Bartoccioni, P. Kakimoto, N. Plana, S. R. Veiga, V. Hernández, N. Vasconcelos, G. Peddinti, A. Adrover, M. Jové, R. Pamplona, I. Gordaliza-Alaguero, E. Calvo, N. Cabré, R. Castro, A. Kuzmanic, M. Boutant, D. Sala, T. Hyotylainen, M. Orešič, J. Fort, E. Errasti-Murugarren, C. M. P. Rodrígues, M. Orozco, J. Joven, C. Cantó, M. Palacin, S. Fernández-Veledo, J. Vendrell, A. Zorzano, *Cell* **2019**, *177*, 881.

[3]	L. Tuo, J. Xiang, X. Pan, J. Hu, H. Tang, L. Liang, J. Xia, Y. Hu, W. Zhang, A. Huang, K. Wang, N. Tang, *Journal of Experimental & Clinical Cancer Research* **2019**, *38*, 50.

[4]	D. E. Kleiner, E. M. Brunt, M. Van Natta, C. Behling, M. J. Contos, O. W. Cummings, L. D. Ferrell, Y. Liu, M. S. Torbenson, A. Unalp-Arida, M. Yeh, A. J. Mccullough, A. J. Sanyal, S. C. R. N. Nonalcoholic, *Hepatology (Baltimore, Md.)* **2005**, *41*, 1313.

[5]	F. Yao, Y. Deng, Y. Zhao, Y. Mei, Y. Zhang, X. Liu, C. Martinez, X. Su, R. R. Rosato, H. Teng, Q. Hang, S. Yap, D. Chen, Y. Wang, M. M. Chen, M. Zhang, H. Liang, D. Xie, X. Chen, H. Zhu, J. C. Chang, M. J. You, Y. Sun, B. Gan, L. Ma, *Nature Communications* **2021**, *12*, 7333.

[6]	J. Wei, F. Liu, Z. Lu, Q. Fei, Y. Ai, P. C. He, H. Shi, X. Cui, R. Su, A. Klungland, G. Jia, J. Chen, C. He, *Molecular Cell* **2018**, *71*, 973.

[7]	X. Zhang, X. Chen, B. Xu, M. Chen, S. Zhu, N. Meng, J. Wang, H. Zhu, D. Chen, J. Liu, G. Yan, *Nature Communications* **2023**, *14*, 3815.
